# Connectivity mapping-based identification of pharmacological inhibitor targeting HDAC6 in aggressive pancreatic ductal adenocarcinoma

**DOI:** 10.1038/s41698-024-00562-5

**Published:** 2024-03-07

**Authors:** Pranita Atri, Ashu Shah, Gopalakrishnan Natarajan, Satyanarayana Rachagani, Sanchita Rauth, Koelina Ganguly, Joseph Carmicheal, Dario Ghersi, Jesse L. Cox, Lynette M. Smith, Maneesh Jain, Sushil Kumar, Moorthy P. Ponnusamy, Parthasarathy Seshacharyulu, Surinder K. Batra

**Affiliations:** 1 Department of Biochemistry and Molecular Biology, University of Nebraska Medical Center, Omaha, NE USA; 2 School of Interdisciplinary Informatics, College of Information Science and Technology, University of Nebraska at Omaha, Omaha, NE USA; 3 Department of Pathology and Microbiology, University of Nebraska Medical Center, Omaha, NE USA; 4 Department of Biostatistics, College of Public Health, University of Nebraska Medical Center, Omaha, NE USA; 5 Eppley Institute for Research in Cancer and Allied Diseases, University of Nebraska Medical Center, Omaha, NE USA; 6 Fred and Pamela Buffett Cancer Center, University of Nebraska Medical Center, Omaha, NE USA

**Keywords:** Targeted therapies, Pancreatic cancer

## Abstract

Pancreatic ductal adenocarcinoma (PDAC) remains highly lethal due to limited therapeutic options and expensive/burdensome drug discovery processes. Utilizing genomic-data-driven Connectivity Mapping (CMAP) to identify a drug closer to real-world PC targeting may improve pancreatic cancer (PC) patient outcomes. Initially, we mapped CMAP data to gene expression from 106 PC patients, identifying nine negatively connected drugs. These drugs were further narrowed down using a similar analysis for PC cell lines, human tumoroids, and patient-derived xenografts datasets, where ISOX emerged as the most potent agent to target PC. We used human and mouse syngeneic PC cells, human and mouse tumoroids, and in vivo mice to assess the ability of ISOX alone and in combination with 5FU to inhibit tumor growth. Global transcriptomic and pathway analysis of the ISOX-LINCS signature identified HDAC 6/cMyc as the target axis for ISOX. Specifically, we discovered that genetic and pharmacological targeting of HDAC 6 affected non-histone protein cMyc acetylation, leading to cMyc instability, thereby disrupting PC growth and metastasis by affecting cancer stemness. Finally, Kras^G12D^ harboring tumoroids and mice responded effectively against ISOX and 5FU treatment by enhancing survival and controlling metastasis incidence. Overall, our data validate ISOX as a new drug to treat advanced PC patients without toxicity to normal cells. Our study supports the clinical utility of ISOX along with 5FU in future PC clinical trials.

## Introduction

Pancreatic ductal adenocarcinoma (PDAC) is the most prevalent type (90%) of pancreatic cancer (PC), with the highest clinical trial failures and projected second position in increased incidence and death rate in 2030^1^. The current standard of care, gemcitabine, capecitabine, 5-fluorouracil (5FU), and FOLFIRINOX, provides a symptomatic improvement in <25% of patients with a dismal five-year survival rate of 12%. Furthermore, the Pancreatic Cancer Action Network (PanCAN) explicitly declares the lack of promising therapeutic modalities as a major confounder for this appalling survival^2^. The current options, including surgery, radiation therapy, chemotherapy, targeted therapy, and immunotherapy, have major limitations. While surgery is the most effective curative option, only 20% of patients are rendered suitable for surgical interventions^3^. The initial response of FOLFIRINOX (oxaliplatin, irinotecan, leucovorin, and 5FU) compared to GEM alone was dramatic with a 21.6-month MOS; it was limited by the high toxicity^4^. Across studies, FOLFIRINOX and Abraxane (ABX), in combination with GEM, have only marginally improved the survival rates by 11 months and 9 months, respectively^5–7^. Further, considering that only 25% of patients respond to GEM and the high degree of toxicities associated with FOLFIRINOX, there is a compelling need to identify new potent therapeutics^8^. While currently, there are 18 monotherapies and 4 combination therapies that the USFDA has approved for use in PC. Furthermore, this aggressive malignancy is resistant to chemotherapy^9^; hence, better molecular-targeted agents are highly needed.

Various computational methods have been established to identify new therapeutics, including the BROAD institute tool, CMAP (https://www.broadinstitute.org/cmap/).CMAP is a big repository of gene expression data compiled from the effects rendered or the transcription readout of treatment by various FDA-approved as well as pre-clinical small molecule inhibitors. Effectively, a positive connection between the disease signature and the gene expression from a drug would mean that a similar signature got affected, i.e., upregulated and downregulated across drug and disease, a neutral connection means no connection, but most importantly, a negative connection would mean a reversal of the user-defined gene expression by the drug. These negative connections would be useful to researchers seeking to identify promising therapeutics that will reverse the gene expression from the biological disease of interest^10,11^.

In the present study, we utilized the CMAP data for screening drugs negatively connected across differentially expressed genes from a range of PDAC microarray datasets and identified nine potential FDA-approved drugs. Among these, CAY10603 (N-[4-[3-[[[7-(hydroxyamino)-7-oxoheptyl] amino] carbonyl]-5-isoxazolyl] phenyl]-1, 1-dimethylethyl ester, carbamic acid), generic name ISOX) was observed to have a most negative connection (−92.3, most significantly targeting the altered gene signature observed across PDAC datasets) for the PDAC gene signature. ISOX is a histone deacetylase (HDAC) inhibitor with the highest efficacy towards HDAC 6, with a potent effect on HDAC3 and HDAC10^12,13^. HDACs expression plays a critical role in pancreatic cancer initiation, progression, and metastasis^14,15^. Among HDACs, HDAC 6 is the only member involved in histone deacetylation and acetylation events and affects nonhistone proteins such as tubulin, p53, cortactin, and heat shock protein 90 (HSP90)^16–18^. We first evaluated the therapeutic effects of ISOX across a spectrum of PDAC cell lines, human PDAC and mouse KPC (Kras^G12D^; p53^R172H^; Pdx-1-Cre^+^) organoids and in an orthotopic pancreatic mouse model. To decipher the mechanism of action of ISOX, we performed RNA-sequencing analysis on ISOX-treated and untreated PC cells, followed by a comprehensive pathway analysis using ingenuity pathway analysis (IPA), a two-way transcription factor enrichment analysis using TFactS (http://www.tfacts.org/) and ENCODE. Our mechanistic findings revealed that ISOX treatment downregulates HDAC 6 with simultaneous enhancement of cMyc acetylation, leading to decreased cMyc protein expression and function. Overall, our studies established multiple in vitro and in vivo functional effects of ISOX with a new mechanism (s) of action via cMyc modulation, affecting apoptosis-related proteins such as activation of caspase3, caspase7, PARP, claspin, and decrease in BCL-X and BCL-2, and.affecting enrichment of pancreatic cancer stem cells (PCSCs) by reducing the expression of PCSC markers.

## Results

### Connectivity mapping analysis identifies ISOX as a potential therapeutic agent for PC

**Fig. 1:**
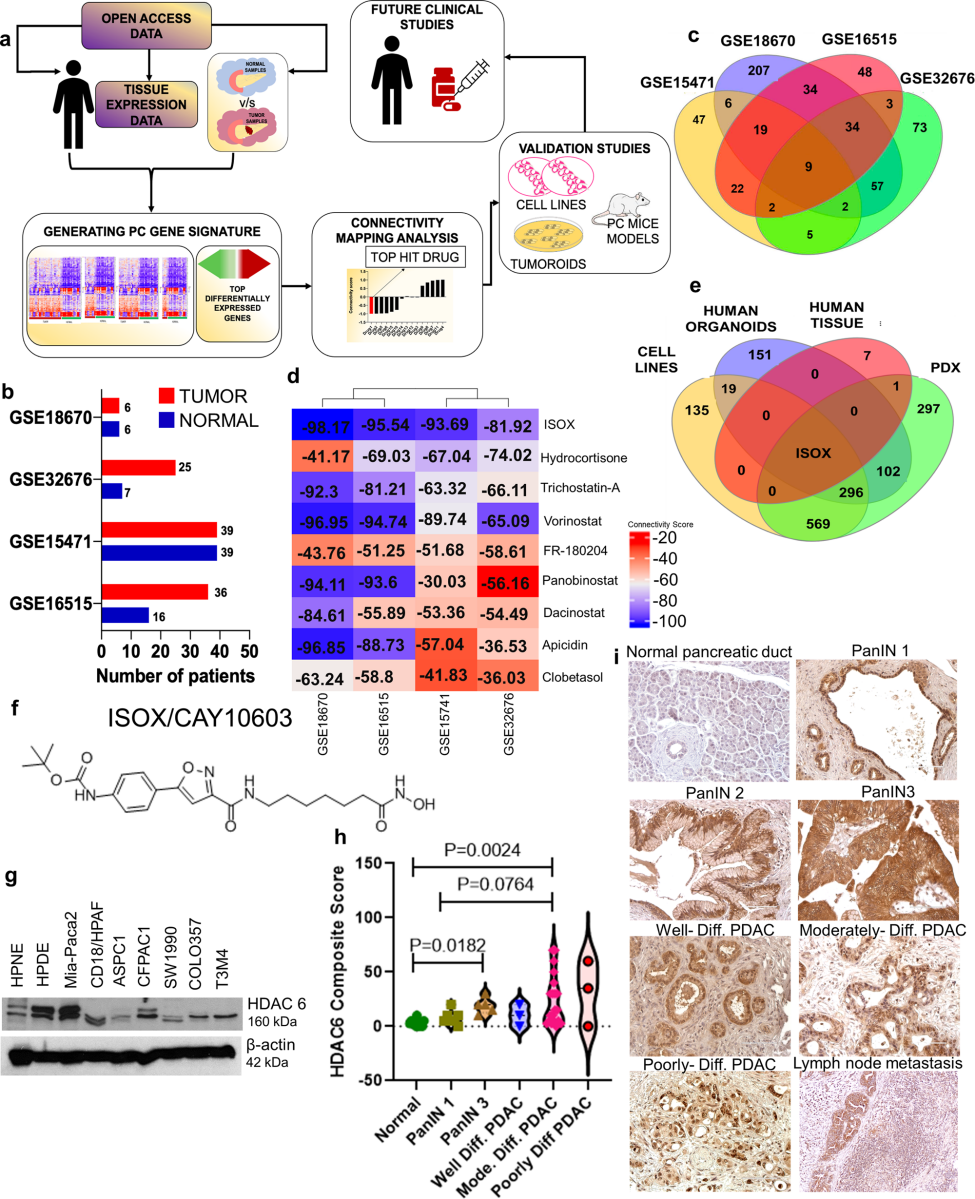
In-silico identification of ISOX as a highly specific therapeutic agent targeting HDAC6 in pancreatic cancer. Connectivity mapping was used to identify negatively connected drugs specific to PC datasets. **a** Schematic representation of overall study design was followed to identify and validate specific therapeutic for PC. **b** The bar graph represents a number of tumors and normal samples within the four microarray datasets GSE18670 (24 samples), GSE32676 (32 samples), GSE15471 (78 samples), and GSE16515 (52 samples). A differential gene expression was carried out using the limma package from R bioconductor to identify top differentially expressed genes between normal and tumor samples. **c** Venn diagrams represent negatively connected drugs across the four datasets. Nine common drugs were identified as being common between all the datasets. **d** Heat maps represent the connectivity scores of all 9 commonly negatively connected drugs. **e** Venn diagram represents a comparison of highly specific drugs identified for PC with PC cell lines, human PC tumoroids, human tissue, and patient-derived xenografts (PDX) datasets. The negatively connected drugs were compared to the nine drugs identified from the human tissue samples. ISOX was identified as the only drug common between all these four models. **f** The structure of ISOX (CAY 10603) as obtained for CMAP shows a clear HDACi moiety. ISOX target HDAC 6 is highly expressed in PC cells and tissues. **g** Immunoblot of HDAC 6 in a panel of normal immortalized pancreatic cells and PC cell lines with β-actin as an internal loading control. **h**, **i** Whipple surgery resected PC tissues were stained with HDAC 6 specific antibody and counterstained with hematoxylin and eosin stains. Representative images were taken using the objective lens of 20× and eyepieces with 10× magnifications. Total magnification of the images is 200× (objective lens (20×) × eyepieces (10×)). Data represented as Dot and violin plot demonstrating the protein expression level of HDAC 6 in near-by normal, PanINs, well, moderately, and poorly differentiated stages of PC (in whole tissue). **h** Linear mixed model show HDAC 6 composite score is significant (*p* = 0.013) between two groups (moderate and normal adjacent to tumor, *p* = 0.011). Representative images of immunohistochemical staining of HDAC 6 in human Whipple surgery resected PC samples with varied adjacent normal, PanINs, and various differentiated stages of PC (Magnification in 200×).

CMAP, a big data repository from the BROAD Institute, collects gene expression data from cell lines treated with a large set of diverse inhibitors. We queried the CMAP database for the differential gene signatures from four (GSE16515, GSE15471, GSE32676, and GSE18670) PC datasets (Fig. 1a–c, and Supplementary Fig. 1a–d). We then assessed the drugs negatively connected to these PC signatures due to their propensity to reverse the disease state’s gene signature. The top 150 up-regulated and down-regulated genes from the differential gene expression were put into a connectivity map to identify negatively connected drugs for each dataset separately. Nine common drugs with a significant negative score (−30 or above), including ISOX, Trichostatin A, Vorinostat, Apicidin, Panobinostat, Hydrocortisone, Dacinostat, FR-180204, and Clobetasol were identified (Fig. 1d). Interestingly, ISOX was the top negative scoring drug across all datasets (Fig. 1e, f). However, the scoring showed a slight variability across the datasets, with drugs like panobinostat showing a high score in two datasets (−94.11 and −93.6) but lower in the other two. Considering this, we next performed a CMAP analysis in datasets from PC cell lines (GSE45757), human tumoroids (GSE107610), and patient-derived xenografts (PDX) (GSE46385) to identify a highly specific agent for PC. The negatively connected drugs across these datasets were compared to the nine commonly identified drugs, and ISOX was the only drug commonly found across all PC datasets (Fig. 1e). Our in-silico analysis suggests that ISOX (CAY10603) would be a novel potential therapeutic agent for PC therapies (Fig. 1f).

### HDACs expression in PDAC cell lines and tissues

Initially, we surveyed the expression pattern of HDAC 6 among a panel of PDAC cell lines and normal immortalized human pancreatic cells. Our results demonstrated that HDAC 6 was highly expressed in the majority of PDAC cells compared to normal epithelial PDAC cells (Fig. 1g). To correlate the expression pattern of HDAC 6 with pre-neoplastic and neoplastic differentiation types of PDAC, we used immunohistochemistry in PDAC tissues obtained from Whipple surgery. Compared to normal pancreas adjacent to tumor (NAT), PanIN1, PanIN3, and PDAC tissues revealed a significantly enhanced cytoplasmic expression in all the differentiation types of PDAC (well, moderate, and poor). Specifically, PDAC with moderate differentiation status showed a dramatic rise in HDAC 6 expression relative to NAT and PanIN3. A linear mixed model was used to compare composite scores between types. A square root transformation was applied before analysis to meet model assumptions. A random effect was included for each subject to account for correlation within the subject. Multiple comparison adjustment was made for pairwise comparisons using the method of Westfall et al.^19^, (Fig. 1h, i, and Supplementary Table 1). Next, we modeled HDAC 6 composite score by type using a linear mixed model. Well and Poor groups were excluded because of a very small number of samples in each of these groups. A square root transformation was applied to normalize the distribution. The overall test was significant (*p* = 0.013), indicating that at least 2 of the groups are different. Pairwise comparisons are shown in Supplementary Table 2. After adjustment for multiple comparisons, there is a significant difference between Moderate and NAT (*p* = 0.011) and a marginal difference between PanIN3 and NAT (*p* = 0.064) (Supplementary Table 2). If we combine the Well, Moderate, and Poorly differentiated tumors into one group (PDAC) for comparison. The overall difference is significant (*p* = 0.018), and NAT is different from PDAC (Supplementary Table 3a and 3b). Further, our pairwise comparisons could not be able to detect the difference in the expression of HDAC 6 between moderate, well, and poor differentiation types due to low sample numbers (Supplementary Table 4). HDAC 6 expression is considerably higher in PanIN3 and moderately differentiated PDAC tissues.

### ISOX inhibits the proliferation of PC cell lines

To validate our in-silico findings, we evaluated the therapeutic efficacy of ISOX on tumor cell proliferation in a panel of PC cell lines, including AsPC1, MiaPaCa2, and CD18/HPAF. The impact of ISOX as monotherapy was first evaluated on cellular proliferation using MTT assay as described in our earlier publications^20^. ISOX was found to be extremely potent in reducing the growth of various PC lines in both dose and time-dependent manner and exhibited IC_50_ values of 0.14–3.80 µM (Supplementary Fig. 2a–c). To understand the maximum and minimum biological effects of ISOX, we applied IC_50_ values of appropriate cell lines with respective time-point along with a lower and higher concentration for in vitro functional and biochemical assays. Interestingly, the varied response of ISOX was observed across the PC cell line panel, further suggesting the disease’s inherent heterogeneity and variable therapy response. Further, to examine the synergy/additive effect of ISOX and 5FU in PC cells, we treated the PC cells with three different doses of 5FU and ISOX alone and a fixed dosage of 5FU with various concentrations of ISOX in combination. We observed that MiaPaCa2 and CD18/HPAF exhibited synergistic cell-killing effects when treated with ISOX (at all doses) in combination with 5FU (Supplementary Fig. 3a–d). To identify the HDAC dependency of ISOX in its mechanism of action, a head-to-head comparison was carried out between ISOX, tubastatin A, and riclinostat. Reassuringly, ISOX performed better in reducing the proliferation of all PC cell lines, while tubastatin A and ricolinostat were unable to induce reduction even at 1–10 µM concentrations, supporting the efficacy of the method and the unique potential of ISOX as a PC therapeutic (Supplementary Fig. 4a–f). Additionally, the toxicity of ISOX on normal pancreatic cells was determined using normal human pancreatic immortalized cell line HPNE at different concentrations from 10 nM to 100 µM^21^. ISOX had minimal effect on HPNE cells (Supplementary Fig. 2d).

### ISOX induces apoptosis, G0/G1, and G2/M arrest in human and mouse syngeneic PC cells and reduces PC cell invasion/migration abilities

**Fig. 2:**
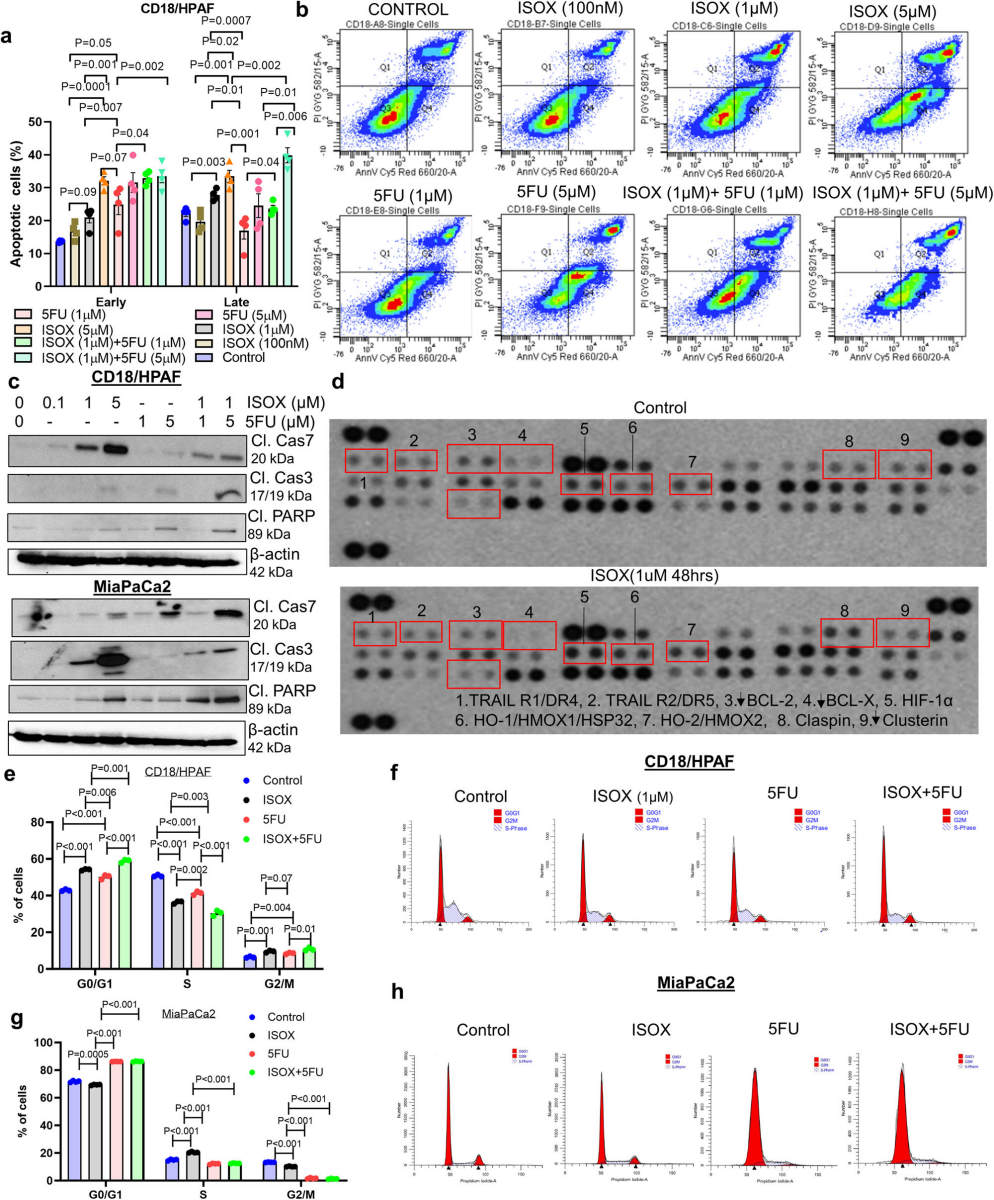
ISOX inhibits PC cell growth by inducing apoptosis and blocking G0/G1 and G2/M phase cell cycle. PC (CD18/HPAF) cells were exposed to varied concentrations of ISOX (100 nM, 1 µM, and 5 µM) alone and in combination with 5 FU (1 µM and 5 µM) (*N* = 4) for 48 h, followed by Annexin V and PI staining. **a** Bar graph represents data on the percentage of PC cell apoptosis induced upon different concentrations of ISOX, 5FU, and a combination of both. Data expressed as mean ± SEM. **b** Representative flow cytometric dot-plot plotting side versus forward-scattered apoptotic cell populations. **c** PC cells (CD18/HPAF and MiaPaCa-2) were treated with ISOX alone and 5FU combination with indicated concentrations, cells were harvested, and lysates were analyzed for apoptotic proteins cleaved caspase 7, cleaved caspase 3, and cleaved PARP by immunoblotting analysis. Beta-actin served as an internal control to ensure equal protein loading. **d** PC cells were treated with ISOX (1 µM) for 48 h, and lysates were analyzed through high-throughput profiling using a human apoptosis array. Representative images of apoptosis array treated with vehicle control (top) and ISOX (bottom). **e**–**h** Both CD18/HPAF and MiaPaCa-2 cells were serum starved to induce cell cycle synchronization for 12 h and treated with indicated concentrations of ISOX and 5FU for a further 48 h with 10% FBS supplemented DMEM media. The response or DNA content distribution in each cell cycle phase was estimated using flow cytometry data. **e**, **g** bar graph with scattered dot plot showing the PC cell populations (%) in each cell cycle phase. **f**, **h** Histogram showing DNA content distribution (in each cell cycle phase) of PC cells stained with propidium iodide.

To further investigate the anti-tumor potential of ISOX, we determined its ability to induce cancer cell death by apoptosis. The apoptotic index was measured using Annexin V and PI staining with flow cytometry. ISOX dose-dependently enhanced both early and late apoptosis alone and in combination with 5FU in CD18/HPAF PC cells (Fig. 2a, b). ISOX treatment resulted in significant induction of late apoptosis in MiaPaCa-2 cells, whereas untreated control cells showed negligible apoptosis (Supplementary Fig. 5a, b). Similarly, ISOX in combination with 5FU significantly increased both early and late apoptosis relative to ISOX and 5FU alone (Supplementary Fig. 5c, d). Supportively, murine syngeneic PC cells harboring Kras^G12D^ and Trp53^R172H^ mutations showed similar apoptosis effects upon ISOX and 5FU treatment with lower concentrations (IC_25_) of drugs. Specifically, we observed a significant increase in early apoptosis and necrosis in combination therapy relative to single drug treatment (Supplementary Fig. 6a–d). Treatment of PC cells with various doses of ISOX and 5FU combination also showed a similar trend in a decrease in proliferation (Supplementary Fig. 7a, b). To better understand the mechanism of action for ISOX-inducing apoptosis, we examined its effect on activating apoptosis pre-requisite markers. We found an activation of Caspase 3 and cleavage of its substrate PARP1 in response to dose-dependent treatment of ISOX alone (Supplementary Fig. 5e) and in combination with 5FU in four different PC cells (Fig. 2c and Supplementary Fig. 5f). Further, to determine the relative level of apoptosis-associated proteins upon ISOX treatment, we treated MiaPaCa-2 cells with/without ISOX, and cell lysates were incubated with nitrocellulose membrane containing 35 different apoptosis-related antibodies. Our results demonstrate that ISOX downregulates Bad, Bax, Bcl-2, Bcl-x, and clusterin while upregulating claspin, Hif-1α, HO-1/HMOX1, HO-2/HMOX2, p27, and phosphoRad17 (Ser635) (Fig. 2d). An initial synchronization followed by propidium iodide (PI) based FACS analysis was performed to assess the impact of ISOX on the cell cycle in PC cells. We observed that both CD18/HPAF and MiaPaCa2 PC cells show significant accumulation of cells in G0/G1 and G2/M (CD18/HPAF) phase arrest upon ISOX alone and in combination with 5FU treatment relative to 5FU alone (Fig. 2e–h). This change was most prominently observed at 48 h post-treatment in additional PC cell lines (AsPC-1 and CFPAC1), with a significant reduction of cells accumulation in the S phase of cell cycle and a statistically significant increase in the G0/G1 phase (Supplementary Fig. 8a–d). Interestingly, CD18/HPAF and MiaPaCa2 showed a G2/M arrest in combination treatment suggesting heterogeneity within the cell lines (Fig. 2e–h). Considering that invasion and migration are hallmarks of cancer cell aggressiveness and that chemotherapeutic agents often fail to inhibit these processes effectively, we next performed matrigel invasion and wound-healing assays using various concentrations of ISOX in PC cells. Interestingly, ISOX reduced the invasion of both cell lines in a dose-dependent manner (Supplementary Fig. 9a–c). Even the lowest concentration of 100 nM of ISOX inhibited the migration of PC cells compared with the untreated control. Taken together, these results suggest the tumor-growth controlling potential of ISOX is through cell cycle arrest, apoptosis, and migration inhibition when assessed as monotherapy and in combination with 5FU.

### ISOX and 5FU combination prevents colony forming ability of human and murine PC cells

Colony formation assay is regarded as the gold standard to measure in vitro tumorigenicity and the ability of a single cell to expand/respond against drug treatment. We treated both human (CD18/HPAF and MiaPaCa2) and mouse (KPC3248) PC cells with ISOX (IC_50_) and 5FU (IC_25_) for 48 h. After treatment withdrawal, cells were incubated for 10 days with a complete medium. Treatment with both ISOX and 5FU significantly reduced the expansion of single cells into colonies. Though we observed few colonies with single drug treatment, ISOX and 5FU combination completely suppressed the colony growth implicating the tumorigenic and in vitro colony growth abrogating effects of ISOX and 5FU (Supplementary Fig. 10a–f).

### ISOX alone and in combination with 5FU reduces the growth and metastasis of PC orthotropic tumors

**Fig. 3:**
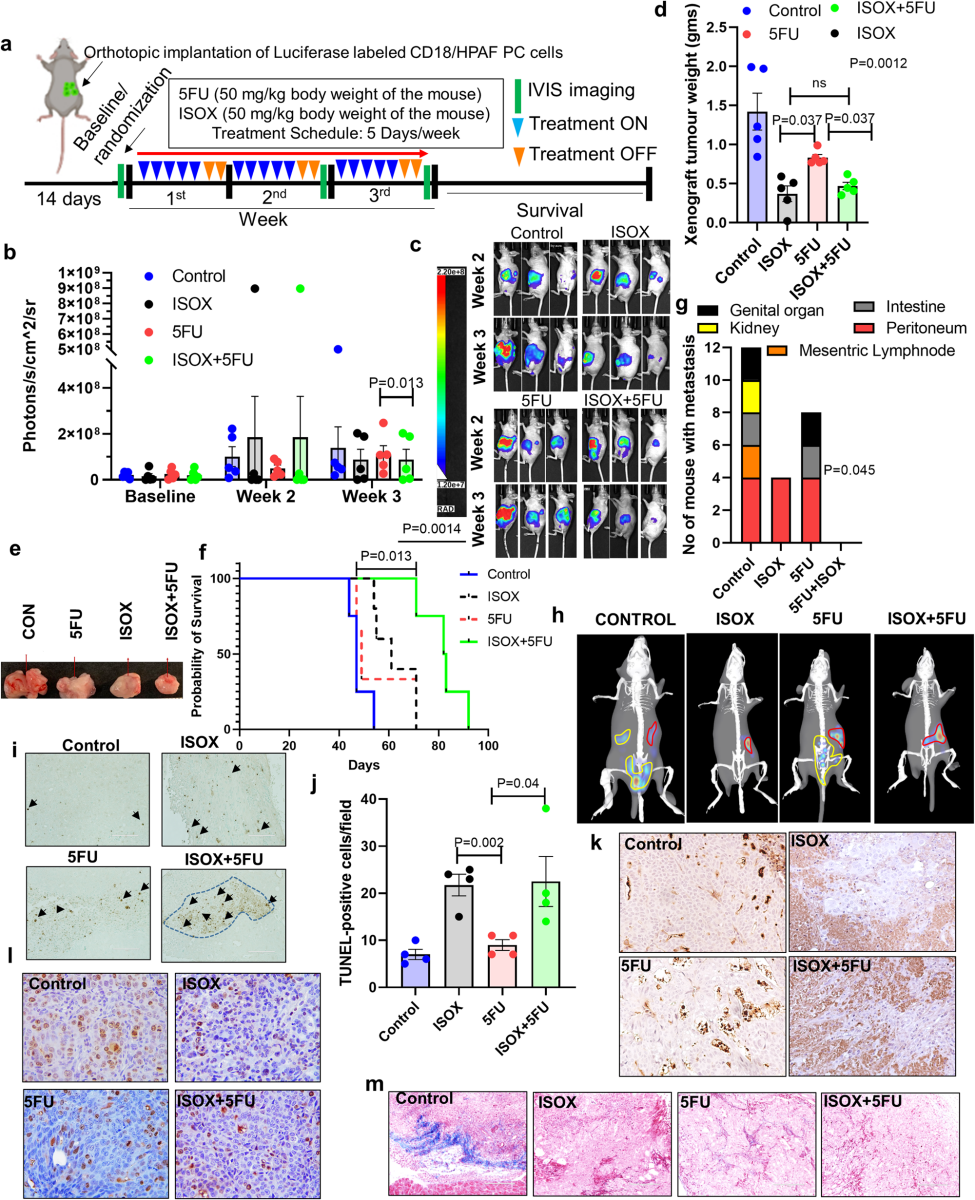
Treatment of ISOX enhances the therapeutic efficacy of 5FU in the immunodeficient mice model. Luciferase-labeled CD18/HPAF PC cells were orthotopically implanted into the head of the pancreas of athymic nude mice. After confirmation of tumor formation through luciferase activity measurement through bioluminescence (BLI), the mice were randomly divided into four groups: control mice (administered with PBS), 5FU alone (50 mg/kg), ISOX alone (50 mg/kg), and both drug combination. Mice were treated for 15 days (3 cycles of 5 days treatment with 2 days off) and monitored through BLI at the beginning, middle (day 10), and end of the treatment (day 15). At the end of day 15, half the mice were sacrificed for tumor weight and metastasis incidence evaluation, and the other half were followed for survival analysis. **a** Scheme showing the treatment and IVIS spectrum imaging schedule. **b** The dot and bar graph represent the luciferase activity of xenograft tumors as evaluated by BLI (Photons/sec/cm^2^/sr), (*N* = 5, *P* = 0.013, Student t-test). Data are expressed as mean ± SEM. **c** Representative tumor response monitoring images against ISOX/5FU and combination at indicated time point of treatment schedule as BLI detection by IVIS machine. **d** Positive correlation of reduction of xenograft weight during the sacrifice. The dot and bar graph represent each treatment group’s tumor weight (gms). Data represents a statistically significant reduction in tumor weight in both ISOX alone and combination compared with 5FU alone group (*N* = 5, *P* = 0.037 (ISOX *vs* 5FU) and *P* = 0.037 (5FU *vs* Combo), Kruskal-Wallis test). Data are expressed as mean ± SEM. **e** Assessment of xenograft size during the post-mortem of all 4 groups. **f** Kaplan-Meier survival curve was constructed based on the survival of mice in all four treatment groups. Assessment of time (days) of death was determined by monitoring each mouse until its death (its own) after post-orthotopic implantation and treatment or as per the recommendation of the in-house veterinarian to sacrifice the mouse due to tumor burden, Overall survival is significant (*P* = 0.0014), and median survival of mice treated with 5FU alone and the combination is better than ISOX alone and control group, Log-rank test (*P* = 0.013). **g** The stacked bar graph represents the number of mice with metastatic spots in each treatment group (*N* = 5, Kruskal-Wallis test, *P* = 0.045 (Mesenteric lymph node Mets.). **h** Representative BLI images with 3-D reconstructions show the high metastasis incidence in the control and 5FU mice but no metastasis in the ISOX alone treated and combination group mice. **i**, **j** TUNEL staining of PC xenograft tissues exposed to drug treatments. Representative light microscopic images of TUNEL positive staining in PC xenograft cells in the 4 treatment groups and the magnification bar in the images represents 100 micrometer (μm) **i**. Dot and bar graph displaying quantitative TUNEL positive results in all the 4 groups (*N* = 3, Students t-test, *P* = 0.002 (5FU *vs* ISOX), *P* = 0.04 (5FU *vs* ISOX + 5FU)), error bars represent standard deviation (sd) **j**. **k**–**m** ISOX and 5FU combinatorial effects on apoptosis, proliferation, and fibrosis. Panels of light microscopic images illustrating the combinatorial and single-drug treatment effects of ISOX and 5FU in xenograft tumors. CD18/HPAF xenograft tumors exposed to ISOX and/or 5FU were stained for Caspase 3 **k** (Total magnification of images is 200×, objective lens (20×) × eyepieces (10×)), and Ki67 (Total magnification of images is 400×, objective lens (40×) × eyepieces (10×)) **l**, and Trichrome mason **m** staining. Magnification bar in representative images of trichrome staining represents 200 micrometer (μm).

Based on the statistically significant therapeutic efficacy of ISOX in 2D cell lines, we further assess the potential of ISOX in the orthotopic xenograft model of PC. Luciferase-labeled CD18/HPAF cells were orthotopically implanted into the pancreas of athymic nude mice. The tumors were allowed to grow for two weeks, and animals were randomized into four treatment groups based on tumor bioluminescence. The control group was given intraperitoneal (i/p) injections of PBS, while the treatment groups were administered with 50 mg/kg 5FU, 50 mg/kg ISOX alone, and a combination of both drugs. All treatments were carried out for 5 days, followed by a 2-day interval, and this routine was followed for 3 cycles (Fig. 3a). The mice were subjected to IVIS imaging on day 0 (baseline), day 10 (week 2), and day 15 (week 3) of treatment regimen. Interestingly, treatment with ISOX alone or in combination with 5FU led to a significant reduction in the size of tumors observed at the end of days 10 (week 2) and 15 (week 3) of treatment schedule, with minimal IVIS signal across multiple mice in the treatment group (Fig. 3b, c). At the end of the experiment, a comparison of tumor weight between untreated and treated mice showed a statistically significant difference between the groups (*p* = 0.0012) (Supplementary Table 5a). The control group has significantly higher tumor weight and size than 5FU (*p* = 0.028), ISOX (*p* = 0.0002), and ISOX + 5FU (*p* = 0.0006) (Supplementary Table 5b, Fig. 3d, e). In addition, overall survival analysis of the mice (day of death defined as a natural death or if suggested by the veterinarian) using the pairwise comparisons shows that the combination treatment (median survival 83 days) has significantly better survival than the control group (*P* = 0.0010) (Supplementary Table 6a–c, and Fig. 3f). Furthermore, these mice were assessed for the total number of metastases as well as the number of metastases at a specific site (peritoneum, mesenteric lymph node, intestine, genital organ, and kidney) were compared between the treatment groups. None differed significantly between the treatment groups with the Kruskal-Wallis test (Supplementary Table 7). However, when we compare specific metastatic spots in various organs, we observed a marginal difference in incidence in “Mesenteric lymph node metastasis”, where 60% of controls have metastasis at that location, 50% of 5FU and 0% of combination and 0% of ISOX group have no metastasis (Supplementary Table 8 and Fig. 3g). Images of these mice at day 10 post-treatment using the 3D-BLIT settings within the IVIS system shows distant metastasis in control and 5FU treated mice whereas, no metastasis was observed in the ISOX alone and its combination with 5-FU treated mice groups (Fig. 3g, h). Similar to the tumoroids, the animal tumors were subjected to tunnel-positive and Caspase 3 staining, which showed an elevated level of apoptosis in the animal group treated with ISOX alone and its combination with 5FU (Fig. 3i–k). This was further corroborated with Ki67 staining, which showed a decrease in the proliferation of PC cells in the combination group compared with single drug treatment (Fig. 3l). Masson’s trichrome staining indicates a decrease in connective fibrous tissues (blue) of ISOX and 5FU alone and combination groups than the control mice group (Fig. 3m).

### ISOX is highly efficacious in inducing growth inhibition in mice and human-derived tumoroids

**Fig. 4:**
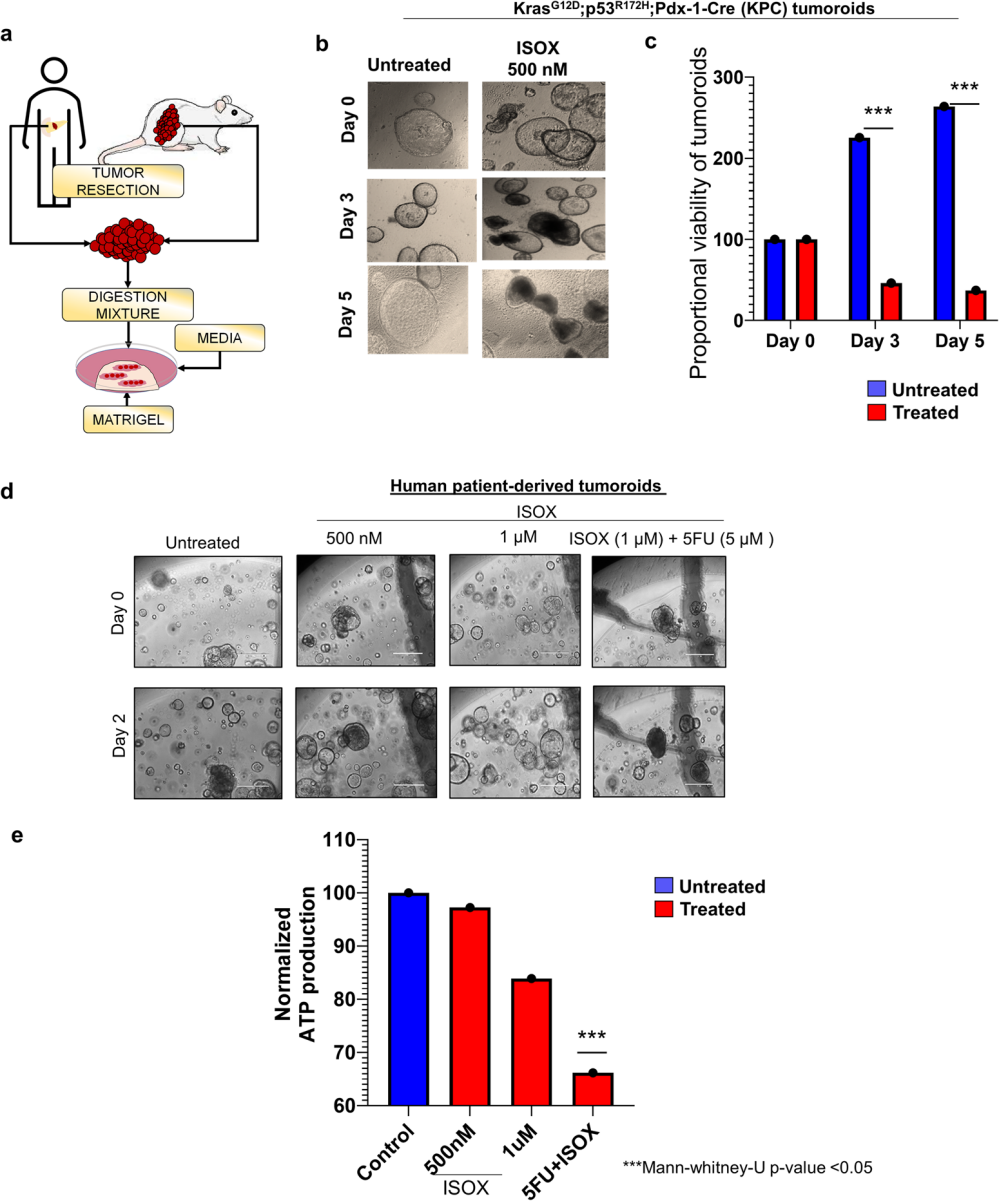
ISOX is highly effective in inhibiting the growth of mice and human-derived tumoroids. To validate the effect of ISOX and/or 5FU observed in PC cells in vitro and in vivo and for clinical translational 3D models are employed. **a** Schematic representation of protocol used for establishing tumoroids from mice and human tumors. Tumors resected from KRAS^G12D^; p53^R172H^; Pdx1- Cre^+^ (KPC) mice and human PC patient donors were incubated with a digestion cocktail of enzymes using a specific protocol and embedded in matrigel. These tumoroids were then used to assess the efficacy of ISOX and /or 5FU. **b** Representative images of ISOX-treated and untreated KPC mouse tumoroids. KPC tumoroids were untreated/treated with 500 nM of ISOX and followed for 5 days. Images were taken at 100× magnification. **c** Bar graph represents the proportional viability of KPC tumoroids in each group across the 5 days. Positive correlation between untreated and ISOX-treated tumoroids obtained through evaluation of tumoroid size on days 0, 3, and 5, ***P < 0.05 (Mann-Whitney-U-Test). **d** Efficacy of ISOX and 5 FU combination in human PC tumoroids. Representative images (The bar represents 5 μm) depicting the dose-dependent effect of ISOX alone and in combination with 5FU (5 µM) in PC patient tumor-derived tumoroids. **e** The efficacy of the ISOX treatment on tumoroids was measured using a CellTiter-Glo® 3D cell Viability assay. Bar graph represents the quantification of ATP production as a consequence of ISOX alone and 5FU combination on Day 2 after drug treatment initiation, ***P < 0.05 (Mann-Whitney-U-Test).

The anti-cancer effects of cancer therapeutics observed in pre-clinical models often cannot be mirrored in clinical models, mainly due to the discordance between the biology of these monolayer cultures and the complex tumor microenvironment of most cancers and specifically complex diseases like PC. In this regard, the 3D cancer tumoroids offer a near-native structure and provide great promise and applicability in drug efficacy studies. The efficacy of ISOX was evaluated in tumoroids-derived from the most commonly used mouse model of PC; Kras^G12D/+^; p53^R172H/+^; Pdx1- Cre^+^ (KPC) mouse that recapitulates human PDAC biology and human PC patient-derived tumoroids (Fig. 4a). The KPC tumoroids were treated with 500 nM of ISOX and followed for 5 days through imaging. A significant reduction in the viability with morphological changes in the tumoroids with a significant reduction in size and visible darkening of the structures (Fig. 4b) were observed across drug-treated groups. The proportional viability of tumoroids was assessed, where the total number of live tumoroids at day 0 was considered as the baseline. Interestingly, the proportional viability reduced significantly by 60% within 3 days of 500 nM of ISOX treatment (Fig. 4c). A similar assessment was conducted in a set of human patient-derived tumoroids using a range of ISOX concentrations (100 nM, 500 nM, 1 µM, and 5 µM) and a combination of ISOX (1 µM) with 5 µM of 5FU. Of note, the significant increase in organoid death (measured as darkness) was observed with ISOX at all concentrations and its combination with 5FU (Fig. 4d, Supplementary Fig. 11a). We performed a 3D cell viability assay to assess the ATP production in these human patient tumoroids as a measure of organoid viability. Dose-dependent reduction of ATP production was observed upon ISOX treatment at 1 µM, which was drastically reduced in tumoroids treated with the combination of ISOX and 5FU. Thus, highly efficacious ISOX is killing human tumoroids (Fig. 4e). These drugs treated human tumoroids were sectioned at the end of the 48-h treatment. H&E staining of these sections supported the initial loss of viability, wherein a loss of structure could be observed with an increase in ISOX concentrations. A tunnel assay (Fig. 3i) in xenograft tissues and caspase 3 staining also supported this observation with the maximum cleaved caspase 3 staining observed in the ISOX and 5FU drug combination group in xenograft tissues and tumoroids (Fig. 3k, Supplementary Fig. 11a, b).

### Mechanism of ISOX action on PC cells

**Fig. 5:**
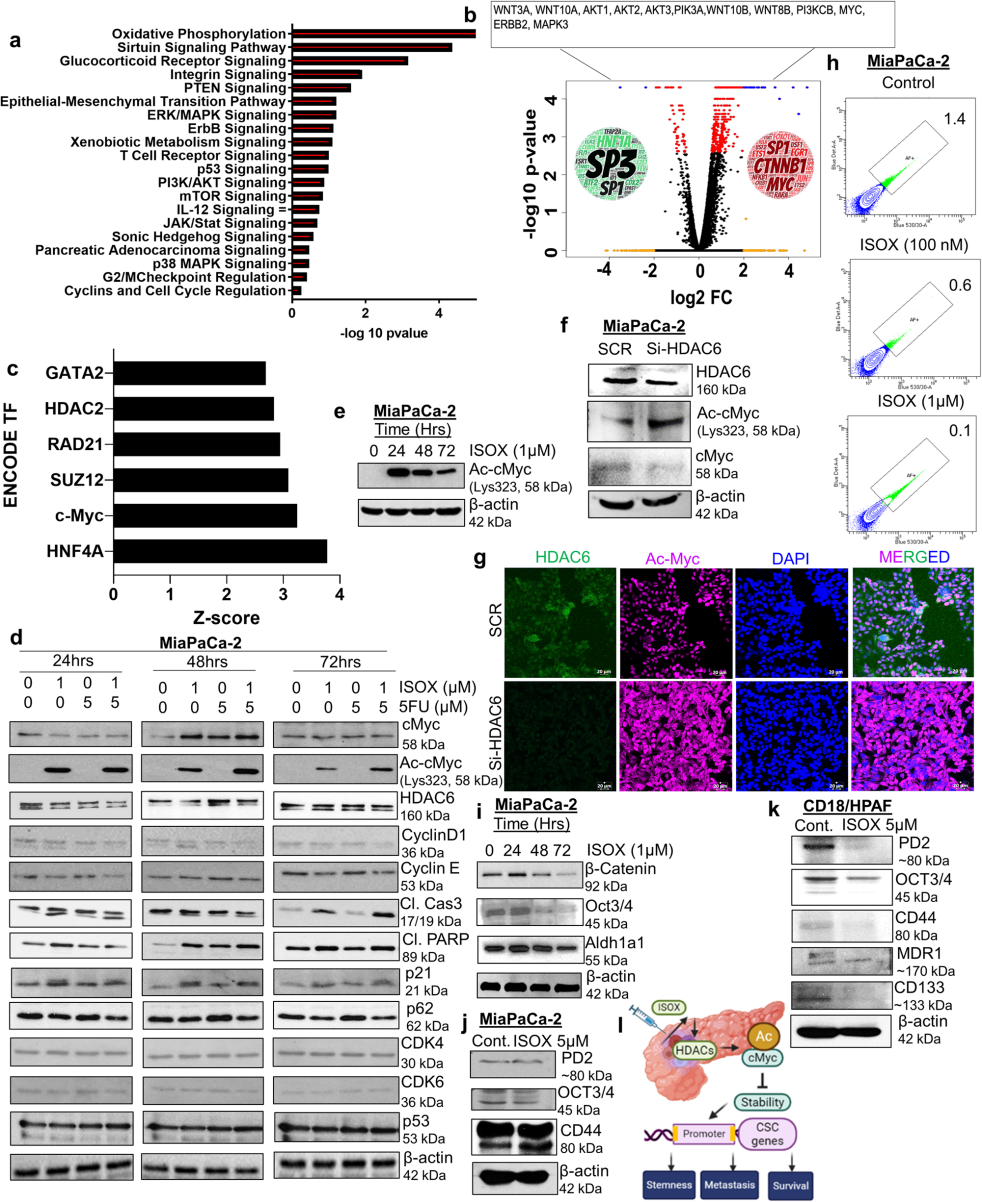
Molecular mechanism(s) of ISOX inhibiting HDAC6 and enhancing cMyc acetylation affecting PC stemness. PC cells (CD18/HPAF) were subjected to ISOX (1uM) treatment for 48 h and processed for transcriptomic analysis. **a** The top differentially responded genes against ISOX were analyzed using ingenuity pathway analysis (IPA). The bar graph represents the enrichment of genes related to oxidative phosphorylation, sirtuins, integrins, EMT, and ERK/MAPK pathways affected by ISOX as observed through IPA. **b** Volcano plot represents RNA-seq analysis of ISOX-treated CD18/HPAF cells. The word cloud within the volcano plot represents transcription factors regulating the genes differentially expressed between untreated and treated samples. **c** Bar graph representation of z-scores from encoding transcription factor analysis of the genes downregulated between untreated and treated samples. Genes downregulated by the treatment were subjected to a transcription factor analysis using the iLINCS (http://www.ilincs.org/ilincs/). Important transcription factors affected by ISOX treatment include HDAC 2, GATA2, and cMyc relative to untreated controls. **d** Immunoblot analysis of molecules associated with pathways identified through RNA seq analysis in ISOX-treated cell lines. PC cells were treated with the indicated concentration of ISOX and/or 5FU for 24, 48, and 72 h. Whole-cell lysates were analyzed for cMyc, acetylated cMyc, HDAC 6, CyclinD1, Cyclin E, Cleaved caspases 3, Cleaved PARP, p21, p62, CDK4, CDK6, and p53. Beta actin served as loading control for all time points. **e** Immunoblot analysis of levels of acetylated cMyc protein in MiaPaCa-2 PC cells treated with ISOX for 24, 48, and 72 h. **f**, **g** Change in levels of acetylated cMyc in MiaPaCa-2 cells with HDAC6 knockdown compared to scramble (SCR) control siRNA. MiaPaCa-2 cells were treated with si-HDAC 6 for 72 h and analyzed for acetylated cMyc, HDAC 6, and -β-actin protein expression using western blot analysis **f** and confocal analysis (The bar in the representative images represents 20 μm) **g**. **h**–**k** ISOX treatment affects cancer stemness-related markers. MiaPaCa-2 cells were treated with indicated concentrations and analyzed for the expression of side population and cancer stemness markers. Representative flow cytometry analyzed side-scatter dot plots of autofluorescence-based side population images of control and ISOX treatment (100 nM, 1 µM) **i**–**k**. Western blot of β-catenin, Oct3/4, ALDH1A1, PD2, and CD44 stemness protein in PC cells (MiaPaCa-2 **i**, **j** and CD18/HPAF **k**) treated with/without ISOX for indicated time points. **l** Overall schema illustrating the mechanistic role of HDAC inhibitor ISOX modulating cMyc acetylation and its epigenetic regulation to influence PC cancer stemness.

To gain an in-depth understanding of the global effects of ISOX, RNA-sequencing analysis of untreated (CD18/HPAF) and treated cells (CD18/HPAF; 1 µM; 48 h) was performed, followed by differential expression analysis. The RNA-sequencing data were further analyzed for pathway analysis of the RNA-seq data showed a high enrichment of sirtuin signaling, epithelial-mesenchymal transition, ERK/MAPK, PI3K-mTOR-AKT, and sonic hedgehog pathway in the treatment group in comparison to the untreated samples (Fig. 5a). Further, gene enrichment analysis revealed that genes associated with WNT (WNT3A, WNT 10 A, WNT 8B, WNT 10B), PI3K (AKT1, AKT2, AKT3, PIK3A, PI3KCB), MYC, ERBB2, and MAPK3 were affected with ISOX treatment compared with untreated controls (Fig. 5b and Supplementary Fig. 12). Further, transcription factor analysis using TFacts and ENCODE tools to assess the transcription factors modulated by ISOX treatment showed MYC as a regulator of the differentially expressed gene. (Fig. 5c). Intriguingly, the interplay between HDACi and acetylation-based regulation of cMyc has been explored in various other cancer settings. Notably, it has been shown that HDACi mediated cMyc acetylation and downregulation, leading to TRAIL-induced apoptosis in acute myeloid leukemia^22^. Interestingly, western blot analyses of ISOX-treated cells showed a significant increase in the acetylated cMyc, α-tubulin, and histone3 (Fig. 5d, Supplementary Fig. 13a, b). Moreover, ISOX treatment alone or in combination with 5-FU resulted in increased cMyc (K323) acetylation and its transient modulation of HDAC 6 key downstream targets such as p21, p62, Cyclin D1, Cyclin E, and CDK6, with no change in CDK2, CDK4, p53, phospho P38, phospho mTOR and total mTOR protein levels. Moreover, an increase in cleaved caspase 3 and cleaved PARP forms was also observed (Fig. 5d, e, and Supplementary Fig. 13a). Our data analyzing the phosphorylation status of cMyc upon ISOX treatment also showed a decrease in threonine58 phosphorylation of cMyc in PC cells (Supplementary Fig. 13c). Additionally, the western blot and confocal results of HDAC 6-specific siRNA inhibition also validated the increase in acetylated cMyc compared to the control group (Fig. 5f, g). We performed Si-RNA-mediated knockdown of HDAC6 to determine whether genetic inhibition of HDAC6 had an inhibitory effect on PC cell growth. As shown in Supplementary Fig. 14a–d, treatment of PC cells (MiaPaCa2 and CD18/HPAF) with HDAC 6 targeting siRNA significantly reduced PC cell proliferation/growth, whereas no such inhibitory effect was observed in scramble vector transfected PC cells. Thus, ISOX inhibition of PC cell growth might be due to its inhibitory potential over HDAC 6 activity. Moreover, to study the global impact of ISOX treatment on PC, a library of integrated network-based cellular signatures (LINCS) was queried for the ISOX signature using the iLINCS (http://www.ilincs.org/ilincs/) web tool. Specifically, the cancer therapeutics response signature from ISOX/CAY10603 was studied for downstream effectors and similarity with other drug signatures, which led to the identification of multiple PC-related genes as ENO2, HOXB6, and CDX2 (Supplementary Fig. 15a, b). Since, ISOX emerged as a candidate from the analysis of inversely regulated genes associated with pancreatic cancer and responding to ISOX treatment, we wanted to investigate further downregulated genes or responsive genes against ISOX other than HDAC6. We extracted the dataset from iLINCS (CT signature) and transcriptomic data from CD18/HPAF and MiaPaCa2 (our RNA sequencing data). We observed that there exist 23 common genes (ID3, KRT15, GSN, HOOK2, DBP, LSR, EPS8L2, RPS6KA1, PTK6, DDIT4, IFITM1, BMP1, SLC29A3, FDXR, RGL3, ID1, ACSF2, LPAR2, CRIP2, IER5L, SERTAD1, BAG1, PSD4) between CT signature and MiaPaCa2, 8 common genes (AGR2, RARRES1, CTSH, LY6D, CXCL5, SLC6A8, ANXA9, CD82) between CT signature and CD18/HPAF, 6 common genes (PFKFB4, MIR210HG, LPIN3, FGFR3, and SH3D21) between CD18/HPAF and MiaPaCa 2, and 1 common gene (GATA2) among CT signature, CD18/HPAF and MiaPaCa2. Next, we performed qPCR analysis on 9 common genes among CT signature, CD18/HPAF and MiaPaCa2 PC cells (Supplementary Fig. 16a). We observed a significant decrease in the transcript level of SLC6A8, CXCL5 and GATA2 along with HDAC 6 in both human PC cells (MiaPaCa2 and CD18/HPAF) (Supplementary Fig. 16b, c). Upon validation in murine KPC 3248 cells, we observed that the common transcription factor GATA2 (among CT signature, CD18/HPAF, and MiaPaCa2) was significantly downregulated by ISOX treatment relative to untreated control (Supplementary Fig. 16d). Overall, our data suggest that ISOX can downregulate pancreatic cancer-specific genes along with HDAC 6 and cMyc acetylation and regulation.

### ISOX treatment affects cancer stem cells through the cMyc pathway

PC cells treated with dose-dependent ISOX showed enhanced acetylation of cMyc (LY323), one of the targets of WNT signaling. Thus, acetylation of cMyc affects its protein stability; subsequently, it will affect will Myc regulated gene expression. On the other hand, Myc and WNT pathways were demonstrated to be involved in cancer stem cells’ self-renewal and chemoresistance nature. We tested the ability of ISOX to characterize its effect on PCSCs PC (MiaPaCa-2) cells treated with a varied dose of ISOX, followed by analysis for autofluorescence positive (AF^+^) staining. Our results demonstrated that ISOX reduced the number of AF^+^ cancer stem cells (Fig. 5h). We also validated this result using Hoechst dye staining in CD18/HPAF PC cells (Supplementary Fig. 13d). Further, to investigate whether ISOX is effective in inhibiting tumor-initiating cell growth, we performed a tumorsphere assay in the Incucyte live imaging system. Dose-dependent treatment of ISOX exhibited significantly reduced MiaPaCa2 and CD18/HPAF-derived tumorsphere relative to vehicle (DMSO) treated controls. Specifically, in MiaPaCa2, all the doses of ISOX are effective and significantly reduced tumorsphere from days 2 to 6. Whereas in CD18/HPAF, only ISOX at 1 μM on days 2–5 seems to be significantly effective (Supplementary Fig. 17a–d). In addition, the treatment effect of ISOX upon key PCSCs markers was also analyzed. As shown in Fig. 5i, time-dependent treatment of ISOX in PC cells reduced PSCSC markers expression (OCT3/4, Aldh1a1, β-catenin) at both 48 and 72 h. Similarly, ISOX alone treatment in PC cells also reduced CSC proteins related to drug resistance, such as PD2, OCT3/4, CD44, MDR1, and CD133 (Fig. 5j, k). To validate our claim that ISOX reduces cancer stemness by affecting downstream of WNT-β-catenin signaling, we performed a WNT- β-catenin reporter assay to observe the modulatory effect of ISOX on WNT- β-catenin signaling. We found that ISOX (500 nM) and HDAC 6 siRNA treatment decreased reporter activity relative to vehicle and scramble control treatment in both human (MiaPaCa2) and mouse syngeneic (KPC3248) PC cells (Supplementary Fig. 18a, b). These results suggest that the anticancer and anti-metastasis effects of ISOX might be due to the inhibition of PCSCs by abrogating of the WNT-β-catenin/cMyc/HDAC 6 axis (Fig. 5l).

## Discussion

Targeting PC has been a challenge to both researchers and clinicians. While various drugs and combinations have been assessed in clinical trials, we have not been able to either achieve promising survival or improve the quality of life of these patients. The failure of these therapies can be attributed to the failure of the “one target at a time” approach, and better methods with multiple gene targeting approaches can prove beneficial. Recent advances in molecular profiling of tumors have yielded distinct molecular signatures amongst patients, which has led to efforts directed toward establishing targeted therapies or devising precision medicine strategies. Efforts have been made to develop tailored treatments for a subset of the patients with one or multiple actionable mutations; the response, however, remains minuscule. The current study uses an in-silico approach to target PC’s whole gene expression profile.

Our global in-silico assessment identified a highly specific and novel therapeutic for PC; ISOX. Through our in-depth analysis using PC cell lines, tumoroid, and orthotopic mice models, we successfully demonstrated the potential of ISOX as a promising therapeutic for PC. ISOX is a histone deacetylase (HDAC) inhibitor with the highest efficacy towards HDAC 6, with a potent effect on HDAC 3 and HDAC 10^13,23^. A comparison of ISOX efficacy with other HDAC inhibitors showed that ISOX is better than other HDAC inhibitors in inhibiting PC cell proliferation. This observation, in conjunction with the initial identification of ISOX based on the global signature of PC, suggested multiple effects of ISOX in PC in addition to HDAC inhibition.

While this background was strong enough to support the exploration of ISOX as a potential therapeutic, our RNA-sequencing analysis helped us to gain insight into the differences that make ISOX more efficacious than the other HDAC inhibitors. In conjunction with a two-way enrichment study for the regulating transcription factors and pathway analysis, the RNA-sequencing analysis helped establish various important pathways like PTEN signaling, ERK signaling, PI3K/AKT/mTOR signaling, etc., as targets for ISOX^24,25^. Furthermore, a combination of studying the ISOX signature from LINCS and our RNA-seq data paved the path to identifying MYC and related pathways as the direct downstream effectors of ISOX action. MYC and its related signaling pathways, like EGFR and PI3K-AKT-MTOR signaling, have been established to have critical roles in PC. Interestingly, studies have established MYC to be regulated by HDAC-dependent acetylation, suggesting a direct downstream effect of the HDAC family proteins^22,26^. This suggests that the direct effect of ISOX on HDACs leads to the direct regulation of cMyc and its downstream pathways. Interestingly, studies have established a multiple-target approach as having great potential in cancer stem cell therapeutics, paving the way for it in a therapeutic targeting approach in metastatic PC^27^.

In summary, this study successfully establishes ISOX as a new therapeutic for PC. ISOX proved to be highly efficacious in PC cell lines, tumoroids, and mice models. More interestingly, through an HDAC-driven mechanism, ISOX effectively targets multiple pathways known to be important in PC. While years of research have gone into identifying drugs affecting single targets, this approach has not been successful in PC owing to its late diagnosis, high complexity, early metastasis, and dense stroma. We have successfully demonstrated the impact of ISOX on master regulators through our study targeting multiple pathways, including HDAC 6, 3, and 10 and cMyc. Owing to the low dose of ISOX action, low toxicity in normal cell lines, and multiple pathways targeting established, it is a unique and highly potent therapy for PC. This comprehensive pre-clinical assessment has led to the identification of ISOX as a potential therapeutic for PC. Few limitations need to be addressed as we move forward. First, the variable effect of ISOX in various cell lines must be studied for specific mechanism differences. Second, studies are required to understand ISOX better in terms of pharmacokinetics/pharmacodynamics and various toxicity assays. Finally, assessment of ISOX in PC progression mouse models and clinical trials will help us establish its use in the clinics and eventually have direct implications for better patient care. Previously, HDAC inhibitors were shown to synergize with anti-PD-L1 checkpoint inhibitors by influencing tumor immunogenicity in ovarian cancer and melanoma^28,29^. In this context, we can test the efficacy of ISOX in combination with 5FU in KPC mouse or syngeneic xenograft models to observe its effect on increased infiltration of CD4^+^ and CD8^+^T cells and decrease of pro-tumorigenic M2-type macrophages resulting in enhanced efficacy of anti-PD-1 or PD-L1 checkpoint inhibitor thereby converting immunologically cold PC to hot tumors. Specifically, we can test whether HDAC 6 inhibitor modulates the transcript of CD274 or CD279, or CD28 genes encoding PD-L1, PD-1, and CTLA-4, respectively, as described previously^28–31^. We believe that this pipeline and ISOX will prove to be beneficial for PC patients.

## Methods

### Patients datasets

Gene expression omnibus (GEO) datasets were queried for datasets containing PC and normal samples. The first step filter used was the keywords “tissue” and “homo sapiens” which identified over 245 datasets. Further, these datasets were filtered on a multifold criterion-the normal and tumor samples within the same dataset, no pre-treatment, and no inherent bias in sample selection, which helped us identify four datasets GSE32676 (25 tumors, 7 normal), GSE15471 (6 tumors, 16 normal), GSE16515 (36 tumors, 16 normal) and GSE18670 (6 tumors, 6 normal). Further, GEO was queried to identify datasets with PC cell lines (GSE45757), tumor xenografts (GSE46385), and human patient-derived tumoroids (GSE107610)^32–38^.

### In silico analysis

The CEL files (Affymetrix raw data files) from each of the identified datasets were downloaded and processed using the “affy” (46) package from R Bioconductor (version 3.6). The expression was assessed using a robust multi-array average (RMA) from the “affy” package. The array probes were converted to gene names using the hgu133plus2.db library. A linear model was fit (using limma) across individual datasets to identify the most differentially expressed genes in tumors in comparison to normal ones. The genes were then arranged according to log_2_ fold changes, and the top 150 upregulated and downregulated genes were assessed for the CMAP analysis.

### Differentially regulated gene validation in PDAC tissues

The top 150 upregulated and top 150 downregulated genes from individual datasets were queried into the connectivity map tool (https://clue.io/). Negatively connected drugs (connectivity score -30 and higher) were compared across datasets to identify the most common drugs across datasets. Differentially expressed gene signatures from GEO of human PC cell lines, PC patient-derived xenografts, and PC tumoroids were queried for CMAP to evaluate the specificity of PC’s identified drug spectrum of PC. The 9 commonly negatively connected drugs from the previous analysis were then compared to negatively connected drugs in each of these studies, and ISOX was identified as the single common drug and hence was chosen for further analysis.

### Cell lines, cell culture, and reagents

Human pancreatic cell lines (AsPC1, MiaPaCa2, CD18/HPAF, and CFPAC1) were obtained from ATCC and routinely confirmed and authenticated using short tandem repeat (STR) profiling during the experiments. Mouse syngeneic KPC3248 was derived from KPC mouse tumor and validated for Kras^G12D^; Trp53^R172H^; Pdx1- Cre^+^ using PCR genotyping using specific primers detecting hot spot mutations. The cell lines were chosen based on the varied genetic background, differentiation status (AsPC1 and MiaPaCa-2; poorly differentiated, CD18/HPAF; well differentiated), and tumor source (MiaPaCa-2; primary tumor, and CD18/HPAF AsPC1; ascitic fluid). Normal immortalized pancreatic nestin-positive epithelial cells (HPNE) were routinely maintained in our lab^39^ The cells were cultured in 10% FBS-supplemented DMEM or RPMI medium supplemented with glutamine and penicillin as suggested and maintained in a cell culture incubator at 5% CO_2_ and 37 °C. All cell lines were periodically tested for mycoplasma contamination by direct staining of mycoplasma DNA using the DAPI and Hoechst dyes staining method. For drug studies, ISOX (CAY10603) was obtained from Cayman Chemicals (CAS number 1045792-66-2), while tubastatin A (CAS number 1252003-15-8) and riclinostat (ACY1215) were purchased from MedChemExpress.

### Cancer cell viability, combination index, and 3D cell viability assay

The viability of human and mouse PC cells against ISOX was tested using dose and time-dependent conditions as described previously^40,41^. The 3D organoids were treated with the indicated concentration of ISOX and/or 5FU and 3D tumoroid cytotoxicity is determined by CellTiter-Glo® 3D cell Viability assay kit and as per manufactures protocol (Promega, Catalog # G9681). The synergy and additive effects of ISOX and 5FU were determined using a combination index. The obtained values were plotted to determine the combination index using CompuSyn software, as done previously. A combination index <1 indicates synergy between two drugs, whereas values > 1 imply the antagonistic action between two drug modes of action^42–44^.

### Matrigel invasion and scratch assay

ISOX effect on inhibition of migration of PC cells was conducted using Matrigel invasion and scratch wound-healing assays^45,46^.

### Immunohistochemistry

Immunohistochemistry (IHC) was performed in the PC sections obtained from PC patients who underwent Whipple surgery at UNMC and xenograft tissues (Caspase 3 and Ki67, Trichrome staining) and organoid sections (Caspase 3 staining) treated with/without ISOX alone and/or 5FU were processed for immunohistochemistry as described previously^45^ and supplementary materials and methods. Briefly, tissue (5 µM thickness) sections were baked overnight at 56 °C followed by deparaffinization using 3 xylene washes, followed by rehydration using a series of ethanol. Endogenous peroxidases were blocked using 3% H_2_O_2_ for 1 h, followed by antigen retrieval in citrate buffer (pH 6) for 15 min. The slides were then blocked using normal horse serum (Vector Laboratories) and incubated overnight with the various primary antibodies. Universal secondary antibodies (Vector Laboratories) were used for 1 h, and the slides were developed using a DAB substrate kit (Vector Laboratories). Hematoxylin was used for nuclear counterstain. Tissues were dehydrated and mounted using permount. All IHC-processed tissues were scored by a pathologist at UNMC based on the previously published composite score criteria^45^.

### Immunoblot analysis

PC cells were treated with ISOX alone and/or 5FU in dose and time-dependent (24, 42, 72 h) conditions. Similarly, protein lysates from si-HDAC 6 transfected cells were also processed. Harvested cells were prepared as protein lysate using RIPA buffer, followed by removing the cell debris by centrifuging at 13,000 rpm at 4 °C. Protein concentrations were measured DC Bio-Rad protein assay kit. Equal concentrations of protein were loaded for the untreated and treated samples^40^. The primary antibodies used for the western blot are detailed in Supplementary Table 9. All the western blots along with molecular marker/size have been provided as uncropped scanned full images in the supplementary files (Supplementary Figs. 19–30).

### Apoptosis assay, cell cycle analysis, and colony formation assay

Flow cytometry-based apoptosis and cell cycle analysis are performed as described previously^45^. Briefly, human (MiaPaCa2, CD18/HPAF) and mouse (KPC3248) PC cells were treated with ISOX alone, 5FU alone, or ISOX plus 5FU combination for 48 h. Further, cells were harvested and processed to detect apoptosis using annexin V (30 min) and propidium iodide staining^47^. The number of PC cells (MiaPaCa2, CD18/HPAF, AsPC-1, CFPAC1) arrested in each cell cycle phase upon ISOX and /or 5FU treatment (after 48 h) was analyzed by flow cytometry-based analysis, as described previously^45^. For colony formation assay, human (MiaPaCa2, CD18/HPAF) and mouse (KPC3248) PC cells were seeded at 250 cells/well in 6 well plates. After 24 h of incubation, ISOX (IC_50_) and 5FU (IC_25_) were added to the PC cells and further incubated for 48 h. After 10–14 days of treatment, the colonies were fixed using methanol, stained with 0.5% crystal violet in methanol, and incubated in the shaker at room temperature for 4 h, followed by rinsing in tap water to remove crystal violet. The number of colonies with each treatment was counted (a colony of 50 cells was considered a colony), and values were plotted using GraphPad Prism software.

### Proteome profiler analysis and TUNEL assay

A human proteome profiler array was purchased (USA R&D Systems Inc., Catalogue # ARY009, Minneapolis, MS, USA) to detect specific molecules involved in inducing the apoptosis effect by ISOX in PC cells. Briefly, PC whole cell lysates were harvested from ISOX-treated and untreated cells. The lysates were incubated on the array of nitrocellulose membranes coated with multiple apoptosis-related antibodies. The analytes in the lysates were detected with streptavidin-HRP conjugated solution followed by visualization using ECL-chemiluminescent reagent. The spotted array intensity was developed by exposing the membrane to X-ray film. TUNEL assay in xenograft tissues and organoids was conducted according to the manual of the in situ Apoptosis detection kit (Abcam, ab206386).

### Transfection, confocal microscopy, and cell proliferation/growth assay

Human siRNA specific for HDAC 6 and control siRNA were purchased from ThermoFisher Scientific (Catalogue # AM51333). MiaPaCa-2 cells were seeded in 6 well plates (5 × 10^5^ seeding density) and transfected with Lipofectamine^TM^ 2000 reagent along with control and HDAC 6-specific siRNA. After 72 h of siRNA treatment, siRNA efficiency and its effect on acetylated MYC were detected by immunoblotting analysis^45,48,49^. Further, the HDAC 6 knockdown effect was validated using confocal microscopy (2.5 × 10^4^ seeding density) using the above-mentioned specific conditions^48^. Further, to functionally validate the impact of HDAC 6 gene disruption, siRNA for HDAC 6 and scramble were transfected in PC cells (MiaPaCa2 and CD18/HPAF) and monitored for growth inhibition from Day 0 to Day 4 (72 h after transfection) using Incucyte Live cell imaging system.

### Side population analysis by autofluorescence and Hoechst dye assays

PC cells (MiaPaCa-2 and CD18/HPAF) were treated with varied concentrations of ISOX. After 48 h, cells were harvested and incubated with Riboflavin (30 mM, Sigma-Aldrich, St. Louis, MO) and DAPI and analyzed for fluorescence excited (AF^+^) cells as side population cells or cancer stem cells using FACS analysis. Similarly, PC cells treated with ISOX were incubated with Hoechst Dye and analyzed for side population using FACS LSR II Green Calibur system (BD Biosciences)^49^.

### Tumorsphere assay

The spheroid formation ability of CD18/HPAF and MiaPaCa 2 cells were evaluated in the presence/absence of ISOX: The cells were suspended in stem cell-specific media and seeded in ultra-low attachment 96-well microplates at 5000 cells per well. After 24 h, the cells were treated with ISOX at 100 nM, 500 nM, and 1 μM doses and further incubated for 5–6 days. The spheroid growth kinetics were monitored in real-time on Incucyte live cell analysis and imaging system, SX3. The images were captured every 4 h for 5–6 days, and each condition was run in sextuples. The images were analyzed using essence software, and the data was generated using virtual masks to surround spheroids. The spheroid growth was calculated and plotted as total area(μm2/image).

### Orthotopic mice model

All animal experiments were approved by the UNMC Institutional Animal Care and Use Committee. Luciferase labeled CD18/HPAF (viability >95%) were orthotopically implanted into the pancreas of athymic nude mice (male and female (1:1)) at 2.5 × 10^5^ cells in 50 µL tissue culture grade PBS. Orthotopic surgery is a potentially painful procedure; hence, we used xylazine (5–16 mg/kg body weight of mouse) and ketamine (100–200 mg/kg body weight of mouse) as anesthesia through intraperitoneal (i.p.) injection mode of administration. We administered buprenorphine extended-release (ER) via subcutaneous injection as an analgesic to overcome postoperative pain in mouse. All the mice were killed as per UNMC IACUC guidelines at the given experimental endpoint using carbon dioxide (CO_2_) inhalation. The mice were then imaged using the small imaging IVIS system (i.p, injection of D-Luciferin) to monitor tumor formation. At the end of 2 weeks following implantation and confirmation of tumor formation, the mice were randomly distributed into four groups- control (PBS), ISOX (50 mg/kg), 5FU (50 mg/kg), and combination (ISOX and 5FU together). The treatment was carried out for 15 days (3 cycles of 5 days continuous followed by 2 days break) with imaging at day 0 (baseline), week s 2 and 3 or days 10 and day 15 of treatment scedule. Half of the mice from every group were sacrificed, and the other half followed for survival. The tumor weight was then assessed for each mouse and compared using a Mann-Whitney U test across groups. For the survival analysis, the day of death was measured either as a natural death or the veterinarian suggested euthanasia. Survival across groups was compared using a log-rank test. The experiment was repeated twice. Once the experiment reached the endpoint, we sacrificed mice, tumors, and other vital organs excised and collected in buffered formalin and liquid nitrogen for further protein and RNA analysis.

### Mouse and human tumoroids treatment

Both KPC mouse and human patient-derived tumoroids were grown and maintained in tumoroid-specific reagents, as discussed in previous lab publications^45,48^. Human PDAC-patient-derived tumoroids were developed from fresh de-identified pancreatic tumors obtained during surgery and procured through the UNMC research tissue bank facility under approved protocol (IRB 440-16-EP UNMC) from the Institutional Review Board (IRB) of the University of Nebraska Medical Center. We have conducted and processed fresh PDAC tissues for human PDAC-tumor organoid generation in compliance with all relevant ethical regulations of UNMC-IRB and the Declaration of Helsinki. The UNMC tissue bank obtains written informed consent from all the human participants as part of the registry/consent process. Briefly, murine and human PDAC tumoroids were treated with ISOX alone or in combination with 5FU for 5 days and processed for H&E, Caspase3, and TUNEL staining^45,48,49^.

### Next-generation sequencing, bioinformatics analysis, and qPCR analysis

RNA from treated (1 µM ISOX, 48 h) and untreated CD18/HPAF were assessed using Illumina TrueSeq (mid-output 75 paired-end) RNA sequencing (RNA seq). The differentially expressed genes from cuffdiff were subjected to a pathway assessment using ingenuity pathway analysis (IPA v01-12) and gene set enrichment analysis (GSEA). Furthermore, the RNA-seq data was used for a transcription factor analysis using the online tool TFacts (http://www.tfacts.org/) and a more detailed analysis using the ENCODE transcription factor tool within the web tool iLINCS (http://www.ilincs.org/ilincs/). We also analyzed the CTRS (v2020) dataset using iLINCS to develop a consensus “CT signature” for ISOX (CAY10603) and related drugs, specifically in the pancreas cancer setting, and further compared it to the genes downregulated by ISOX treatment (1 μM) in our RNA-seq data obtained from CD18/HPAF and MiaPaCa2 PC cells to identify commonly affected genes in all the settings. Further, the common genes identified through CT signature, transcriptomic analysis of CD18/HPAF and MiaPaCa2 PC cells were validated for their expression through qPCR upon ISOX treatment in human and mouse PC cells. The primers used in the study are listed as Supplementary Table 10.

### TCF/LEF luciferase reporter assay

The Wnt/β-catenin signaling activity was measured by using a TCF/LEF reporter kit (BPS Biosciences, San Diego, CA). Both human (MiaPaCa2) and mouse syngeneic (KPC3248) PC cells were seeded at 10,000 cells per well in a 96-well clear bottom plate. The next day, the cells were transfected with TCF/LEF luciferase reporter vector followed by the addition of ISOX (500 nM), scrambled, and HDAC 6-si-RNA (20 nM). After 24 h, the cells were lysed and analyzed for luciferase activity using a Dual-luciferase reporter assay kit (Promega). The manufacturer supplied constitutively expressing the Renilla luciferase vector, which was used as a negative control.

### Statistical analysis

All statistical analyses were conducted using SAS software version 9.4 was used for analysis (SAS Institute Inc., Cary, NC). For IHC analysis, statistics are provided for the multiplication of staining intensity and percentage, resulting in a composite score. A linear mixed model was used to compare composite scores between types. A square root transformation was applied before analysis to meet model assumptions. A random effect was included for each subject to account for correlation within the subject. Multiple comparison adjustment was made for pairwise comparisons using the method of Westfall (1997)^19^. For the in vivo model, the Kruskal-Wallis test was used to compare tumor weight between the treatment groups, the Wilcoxon rank sum test for pairwise comparisons, using Hochberg’s method to control the familywise error rate under independence. The Kaplan-Meier method was used to estimate overall survival distributions, and log-rank was used to compare between groups, with pairwise comparisons adjustment using Tukey’s method. Fisher’s exact test was used to compare the incidence of metastases at various organ sites. The number of metastases was compared between groups with the Kruskal-Wallis test. As published previously, Student’s t-test was used for all in vitro assays^45^. Cell growth analysis upon HDAC 6 SiRNA and scramble vector treatment in PC cells were calculated using the Incucyte live cell imaging values. 2-way ANOVA was used with Sidak multiple Comparisons test with a single-fold variance.

### Reporting summary

Further information on research design is available in the Nature Research Reporting Summary linked to this article.

### Supplementary information

Reporting Summary

Supplementary information

## Data Availability

All transcriptomic data associated with this research paper are available from the corresponding author upon reasonable request. RNA sequencing data sets are deposited at the GEO repository under the accession number GSE240597. We used ”R” programming for data analysis and data visualization.
