# Supplementary material for: Connectivity mapping-based identification of pharmacological inhibitor targeting HDAC6 in aggressive pancreatic ductal adenocarcinoma
Source: NPJ Precis Oncol. 2024 Mar 7;8:66. doi: 10.1038/s41698-024-00562-5 (PMC10920818; doi:10.1038/s41698-024-00562-5)

Supplementary Information file for

Connectivity mapping-based identification of pharmacological inhibitor targeting HDAC6 in aggressive pancreatic ductal adenocarcinoma

Supplementary Table 1 Summary statistics for HDAC6 expression in human pancreatic cancer tissues.

Label	Type	N	Mean	SD	Median	Minimum	Maximum
Composite score	NAT	11	3.64	3.03	5	0	10
	PANIN1	7	7.86	6.36	5	0	20
	PANIN3	5	19	7.42	20	10	30
	Well	3	10	10	10	0	20
	Moderate	20	24.13	23.17	15	0	70
	Poor	3	31.67	30.14	35	0	60
Intensity	NAT	11	0.36	0.23	0.5	0	0.5
	PANIN1	7	0.5	0.29	0.5	0	1
	PANIN3	5	0.8	0.27	1	0.5	1
	Well	3	0.5	0.5	0.5	0	1
	Moderate	20	0.68	0.29	0.5	0	1
	Poor	3	0.5	0.5	0.5	0	1
percent staining	NAT	11	7.27	6.07	10	0	20
	PANIN1	7	14.29	12.72	10	0	40
	PANIN3	5	24	5.48	20	20	30
	Well	3	13.33	11.55	20	0	20
	Moderate	20	29.25	22.55	30	0	70
	Poor	3	43.33	37.86	60	0	70

Supplementary Table 2. HDAC6 composite score comparison between groups by Pairwise comparisons

Type 1	Type 2	Unadjusted p-value	Adjusted p-value
Moderate	NAT	0.0024	0.0113
Moderate	PaniN3	0.9708	0.9708
Moderate	Panin1	0.0764	0.2075
NAT	PaniN3	0.0182	0.0642
NAT	Panin1	0.2331	0.4045
PaniN3	Panin1	0.1588	0.3402

Supplementary Table 3

Supplementary Table 3A. Summary of statistical analysis by group

Groups	N	Mean	Std Dev	Median	Minimum	Maximum
NAT	11	3.64	3.03	5.00	0.00	10.00
PANIN1	7	7.86	6.36	5.00	0.00	20.00
PANIN3	5	19.00	7.42	20.00	10.00	30.00
PDAC	26	23.37	22.78	15.00	0.00	70.00

Supplementary Table 3B. HDAC6 composite score comparison between groups by pairwise comparisons.

Type 1	Type 2	Unadjusted p-value	Adjusted p-value
NAT	PANIN1	0.3809	0.6095
NAT	PANIN3	0.0285	0.0972
NAT	PDAC	0.0039	0.0181
PANIN1	PANIN3	0.1618	0.3414
PANIN1	PDAC	0.0902	0.2376
PANIN3	PDAC	0.8536	0.8536

Supplementary Table 4. HDAC6 composite score comparison between well, moderate, and poor differentiation types of PDAC by pairwise comparisons

Type 1	Type 2	Unadjusted p-value	Adjusted p-value
Moderate	Poor	0.8893	0.8893
Moderate	Well	0.4854	0.6657
Poor	Well	0.524	0.6657

Supplementary Table 5

Supplementary Table 5A. Tumor weight comparison between groups

	**Group**	**N**	**Mean**	**Std Dev**	**Median**	**Minimum**	**Maximum**	**p-value**
Tumor weight	5FU	5	0.83	0.09	0.79	0.77	0.99	0.0012
	Control	5	1.42	0.53	1.23	0.84	1.99	
	ISOX	5	0.37	0.22	0.44	0.02	0.59	
	ISOX+5FU	5	0.47	0.11	0.45	0.35	0.62	

Supplementary Table 5B. Tumor weight comparison between groups – Pairwise comparisons

**Effect**	**Group**	**_Group**	**Raw p-value**	**Adjustment**	**Adjusted p-value**
Group	5FU	Control	0.0212	Hochberg	0.042
Group	5FU	ISOX	0.0119	Hochberg	0.037
Group	5FU	ISOX+5FU	0.0119	Hochberg	0.037
Group	Control	ISOX	0.0122	Hochberg	0.037
Group	Control	ISOX+5FU	0.0122	Hochberg	0.037
Group	ISOX	ISOX+5FU	0.5309	Hochberg	0.53

Supplementary Table 6

Supplementary Table 6A. Overall survival distributions among treatment groups

Test of Equality over Strata			
**Test**	**Chi-Square**	**DF**	**Pr > Chi-Square**
Log-Rank	15.6051	3	0.0014

Supplementary Table 6B. Pairwise comparison for survival among treatment groups

Adjustment for Multiple Comparisons for the Logrank Test				
**Strata Comparison**		**Chi-Square**	**p-Values**	
**group**	**group**		**Raw**	**Tukey-Kramer**
5FU	Comb	6.1086	0.0135	0.0644
5FU	Control	1.3450	0.2462	0.6523
5FU	ISOX	0.0904	0.7636	0.9906
Comb	Control	14.0584	0.0002	0.0010
Comb	ISOX	2.9603	0.0853	0.3129
Control	ISOX	1.5813	0.2086	0.5901

Supplementary Table 6C. Comparison for median of survival among treatment groups

Analysis Variable: Days				
**Group**	**N**	**Median**	**Minimum**	**Maximum**
5FU	3	49	47	71
Comb	4	83	71	92
Control	4	47	44	54
ISOX	5	61	54	71

Supplementary Table 7 Total number of metastases between groups.

Variable	Group	N	Mean	Std Dev	Median	Minimum	Maximum	p-value
Total number of Metastases (Mets.).	5FU	4	3	2.9	3	0	6	0.13
	Combo	5	0	0	0	0	0	
	Control	5	2.8	3.1	2	0	7	
	ISOX	5	1.6	1.8	1	0	4	
Peritoneum Mets.	5FU	4	1	0.8	1	0	2	0.14
	Combo	5	0	0	0	0	0	
	Control	5	0.8	0.8	1	0	2	
	ISOX	5	0.8	0.8	1	0	2	
Mesentric lymphnode_Mets.	5FU	4	0.5	0.6	0.5	0	1	0.064
	Combo	5	0	0	0	0	0	
	Control	5	0.8	0.8	1	0	2	
	ISOX	5	0	0	0	0	0	
Intestinal_Mets.	5FU	4	0.5	0.6	0.5	0	1	0.38
	Combo	5	0	0	0	0	0	
	Control	5	0.4	0.5	0	0	1	
	ISOX	5	0.4	0.5	0	0	1	
Kidney_Mets.	5FU	4	0.5	0.6	0.5	0	1	0.14
	Combo	5	0	0	0	0	0	
	Control	5	0.4	0.5	0	0	1	
	ISOX	5	0	0	0	0	0	
Genital organ Mets.	5FU	4	0.5	0.6	0.5	0	1	0.38
	Combo	5	0	0	0	0	0	
	Control	5	0.4	0.5	0	0	1	
	ISOX	5	0.4	0.5	0	0	1	

Supplementary Table 8. Comparison of incidence of metastases at various sites

		Groups				
**Metastasis incidence to specific site**	**Frequency Col Pct**	**5FU (n=4)**	**Comb (n=5)**	**Control (n=5)**	**ISOX (n=5)**	**P-value**
Peritoneum	No	1<br>25.00	5<br>100.00	2<br>40.00	2<br>40.00	0.1015
	Yes	3<br>75.00	0<br>0.00	3<br>60.00	3<br>60.00	
Mesenteric lymph node	No	2<br>50.00	5<br>100.00	2<br>40.00	5<br>100.00	0.0454
	Yes	2<br>50.00	0<br>0.00	3<br>60.00	0<br>0.00	
Intestine	No	2<br>50.00	5<br>100.00	3<br>60.00	3<br>60.00	0.4656
	Yes	2<br>50.00	0<br>0.00	2<br>40.00	2<br>40.00	
kidney	No	2<br>50.00	5<br>100.00	3<br>60.00	5<br>100.00	0.1744
	Yes	2<br>50.00	0<br>0.00	2<br>40.00	0<br>0.00	
Genital organ	No	2<br>50.00	5<br>100.00	3<br>60.00	3<br>60.00	0.4656
	Yes	2<br>50.00	0<br>0.00	2<br>40.00	2<br>40.00	

*If a mouse had at least 1 metastasis at a particular site, it was indicated as a yes.

Supplementary Table 9

List of antibodies used in western blot, immunohistochemistry, and confocal microscopy.

	Antibody	Company name, Clone # and/or catalog#	Dilution	Analysis performed
HDAC6	Mouse monoclonal anti-HDAC6	Santa Cruz Biotechnology, D-11, sc-28386	1:1000	IB
Cl. Cas3	Rabbit polyclonal anti-Cleaved Caspase 3	Cell Signaling Technology, D175, 9661S	1:1000	IB
Cl. Cas7	Rabbit polyclonal anti-Cleaved Caspase 7	Cell Signaling Technology, D198, 9491S	1:1000	IB
Cl. PARP	Rabbit monoclonal anti-Cleaved PARP (Asp214)	Cell Signaling Technology, D64E10,5625S	1:1000	IB
cMyc	Rabbit monoclonal anti-cMyc/N-Myc	Cell Signaling Technology, D3N8F, 13987	1:1000	IB, IHC, and ICC
Phospho-cMyc (T58)	Rabbit monoclonal anti-Phospho-cMyc (Thr58)	Cell Signaling Technology, E4Z2K, 46650	1:1000	IB
Ac-cMyc (Lys323)	Rabbit polyclonal anti-acetyl-c-Myc (Lys323)	Sigma-Aldrich, Cat. # ABE26	1:1000	IB/ICC
Acetyl-Histone H3	Rabbit polyclonal anti-acetyl-Histone H3 (Lys9/Lys14)	Cell Signaling Technology, #9677	1:1000	IB
Cyclin D1	Rabbit polyclonal anti-Cyclin D1	Cell Signaling Technology, #2922	1:1000	IB
Cyclin E	Mouse monoclonal anti-Cyclin E	Santa Cruz Biotechnology, E-4, SC377100	1:1000	IB
p21	Rabbit monoclonal anti-p21 (Waf1/Cip1)	Cell Signaling Technology, 12D1, 2947S	1:1000	IB
p62	Rabbit polyclonal anti-p62/SQSTM1	Cell Signaling Technology, #5114	1:1000	IB
p53	Mouse monoclonal anti-p53	Santa Cruz Biotechnology, SC-126, DO1	1:1000	IB
CDK4	Rabbit monoclonal anti-CDK4	Cell Signaling Technology, #12790, D9G3E	1:1000	IB
CDK6	Rabbit monoclonal anti-CDK6	Cell Signaling Technology, #13331, D4S8S	1:1000	IB
CDK2	Rabbit monoclonal anti-CDK4	Cell Signaling Technology, #2546, 78B2	1:1000	IB
β-Catenin	Rabbit monoclonal anti-β-Catenin	Cell Signaling Technology, D10A8, 8480S	1:1000	IB
Oct3/4	Mouse monoclonal anti-Oct3/4	Santa Cruz Biotechnology, C-10, SC-5279	1:1000	IB
PD2	Rabbit polyclonal anti-PD2	Bethyl Laboratories, A300-172A	1:5000	IB
MDR1	Rabbit monoclonal anti-MDR1	Cell Signaling Technology, E1Y7B, 13342	1:1000	IB
CD44	Mouse monoclonal anti-CD44	Cell Signaling Technology, 8E2, 5640	1:1000	IB
CD133	Rabbit polyclonal Anti-CD133	Abcam, ab19898	1:1000	IB
ALDH1A1	Rabbit polyclonal Anti-ALDH1A1	Cell Signaling Technology, #54135, D9Q8E	1:1000	IB
mTOR	Rabbit monoclonal anti-mTOR	Cell signaling Technology (7C10), Ab#2983	1:1000	IB
Phospho-mTOR	Mouse monoclonal anti-Phospho-mTOR (Ser 2448)	Cell Signaling Technology, #2971	1:1000	IB
Phospho-p38	Rabbit monoclonal anti-phospho p38	Cell signaling Technology (D3F9), #4511	1:1000	IB
β-actin	Mouse monoclonal anti-β-actin	Sigma-Aldrich, A1978, AC-15.	1:500	IB

Supplementary Table. 10

Human and mouse primers used in this study for qPCR analysis.

S.No	Gene	Forward primer	Reverse primer	Species
1	AGR2	AGCAGTTTGTCCTCCTCAATC	TCTTCCAGTGATATCGGCTCTA	Human
2	RARRES1	CGCATTCACTTGGTCTGGTA	CTTCTGGTGTCTGTAGCTCTTG	Human
3	CTSH	CACACCATCCCTTCCCTTATC	CTTGTTCTACAGACATCCCAGAG	Human
4	LY6D	CCAGCAACTGCAAGCATTC	CACAGTCCTTCTTCACCAGATT	Human
5	CXCL5	GGACGGTGGAAACAAGGAA	GGAATCCAGGAAGAAAGCTAACTA	Human
6	SLC6A8	TGCGTGAGTACGGAGAGTAT	GTGGTAAGGAGAGGATGGTTTG	Human
7	ANXA9	GGAAGTCCCTCTACTCTTCTCT	GTTCAGCCAAACACGGAAATC	Human
8	CD82	CTTGCCCATCCTGACTGAAA	AGTTGCTAAGAGAACCCTGATG	Human
9	GATA2	GACGACAACCACCACCTTAT	AGTCTGGATCCCTTCCTTCT	Human
10	HDAC6	GGAGGGTCCTTATCGTAGATTG	GTAGCGGTGGATGGAGAAATAG	Human
11	Agr2	GCTCATCTGGACTCAGACATAC	CATAGACCAGGTTGAGGAGAAC	Mouse
12	Rarres1	CTGCCGTATTCACTTGGTCT	CCATAGCTGATGCTTCCATAGT	Mouse
13	Ctsh	TGGACCTAGAATCGTCTCTCTT	CCAGGGAGGTATTTACTGTGTG	Mouse
14	Ly6d	TTATGGCTCTCTGCCTGTAATG	TGCCTCCATCATGGCTAAAG	Mouse
15	Cxcl5	TGAACTCCCTGCTTTGATGAG	CCGATAGTGTGACAGATAGGAAAG	Mouse
16	Slc6a8	TTCCTTCACATCCTCACCTTTC	GGAGACCATACAGAACATCCATAC	Mouse
17	Anxa9	TGAGGATGTAGCCTTGGAAATC	CCACCTGGAAGTCATGCTTAT	Mouse
18	Cd82	GTCTCCACCCTGAAGTCATATTG	GAAGAATTGCCTACCCTGTCTC	Mouse
19	Gata2	GGAGAAAGGAGTAGGCAAGAAG	CCCAAGAACACAAATAGCACAC	Mouse
20	Hdac6	CCCAATCTAGCGGAGGTAAAG	CACTCTTGTCTCAGGGTTCAG	Mouse

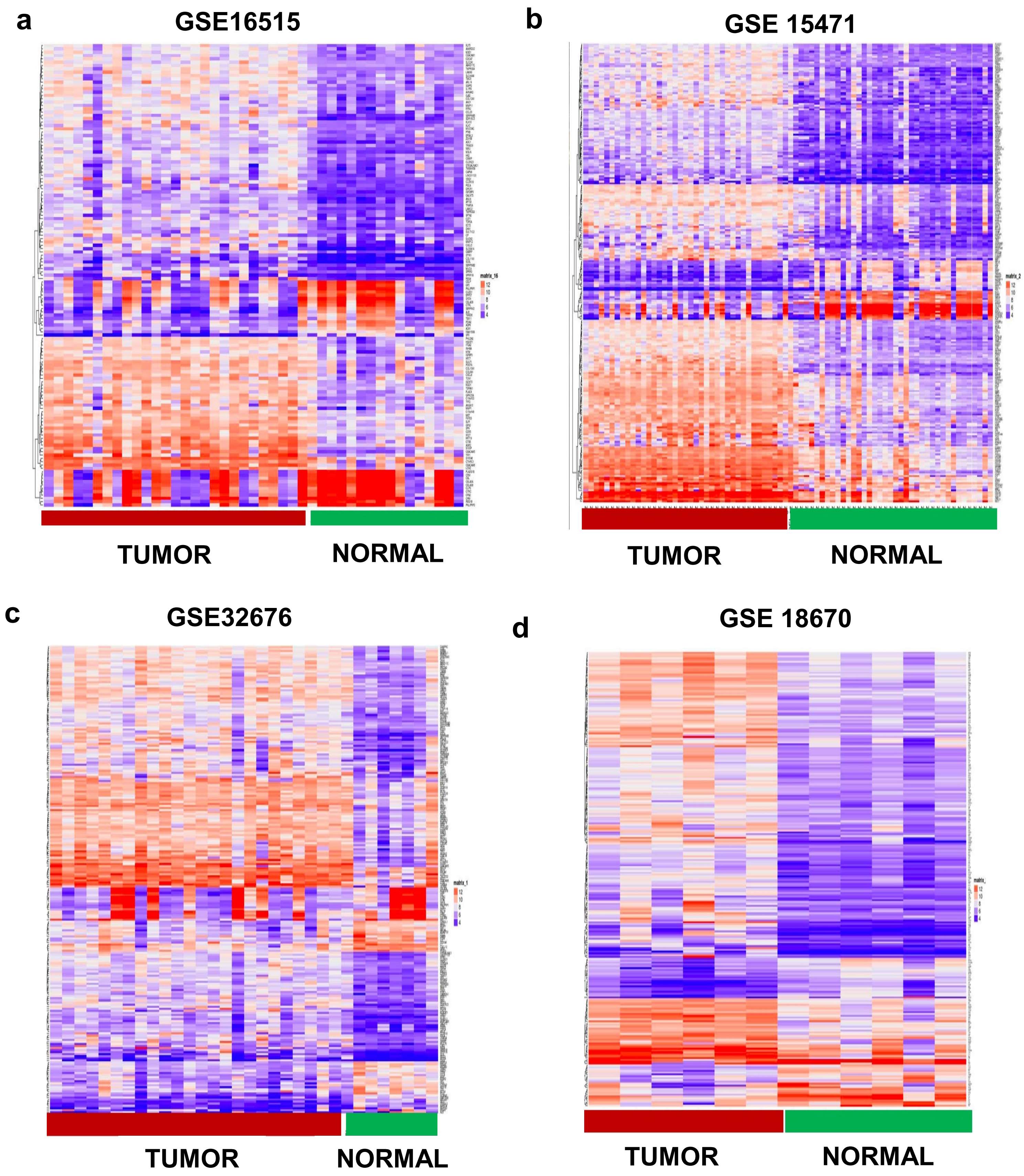

Supplementary Figure 1: *In silico*-based identification of USFDA drugs strongly associated with pancreatic cancer differential gene signatures. Heatmap showing the top 150 up-regulated and down-regulated genes that are differentially expressed in GSEs 16515 (Normal = 16 and Tumor = 36) (**A**), 15471 (Normal = 16 and Tumor = 6) (**B**), 32676 (Normal = 7 and Tumor = 25) (**C**), and 18670 (Normal = 6 and Tumor = 6) (**D**). The differentially expressed gene signatures were matched with the drug profile in the connectivity map to identify negatively connected drugs for each dataset separately.

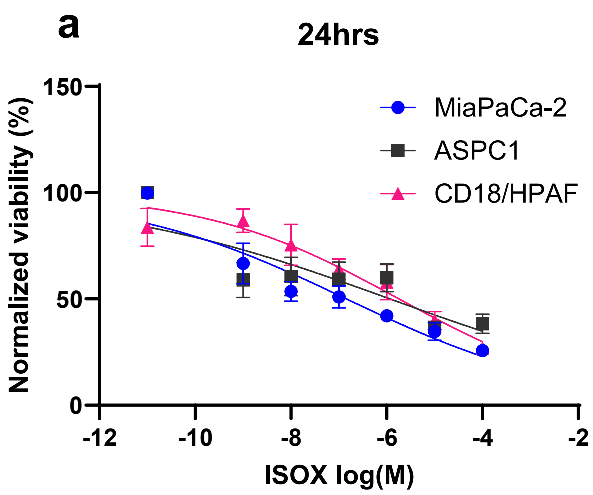

Cell Line	IC_{50} (μM)
MiaPaca-2	0.144
AsPC1	1.177
CD18/HPAF	1.815

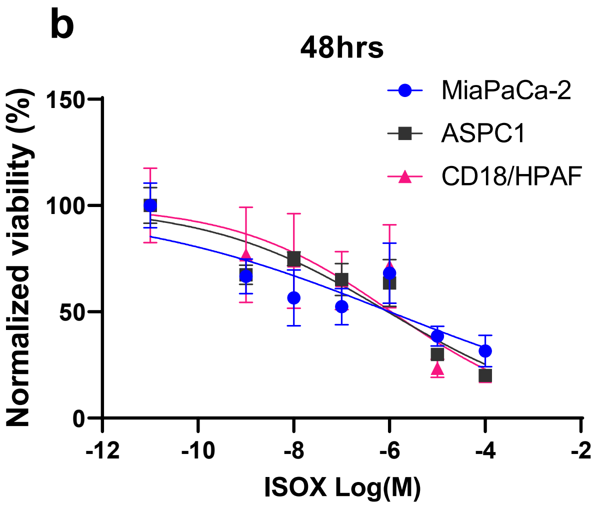

Cell Line	IC_{50} (μM)
MiaPaca-2	1.017
AsPC1	0.926
CD18/HPAF	1.096

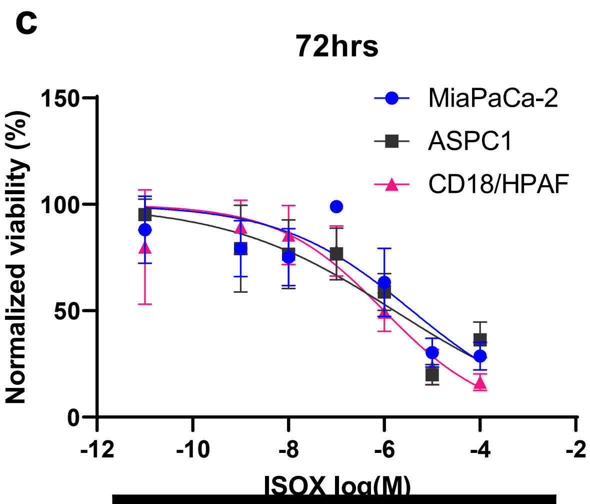

Cell Line	IC_{50} (μM)
MiaPaca-2	3.808
AsPC1	1.636
CD18/HPAF	0.989

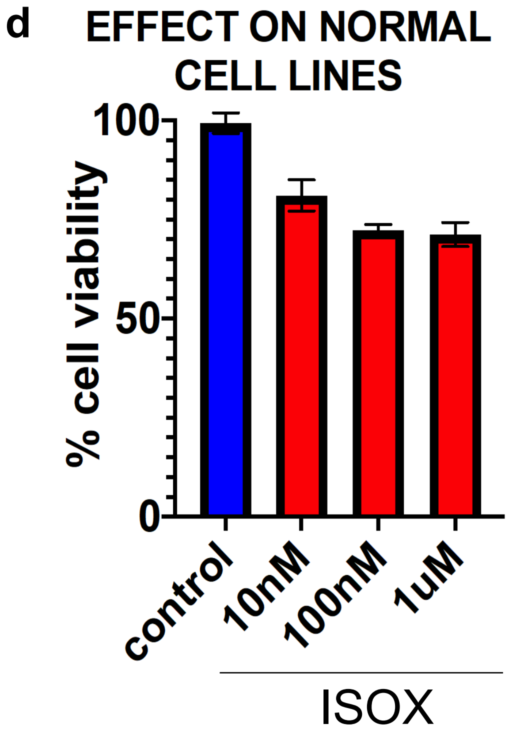

Supplementary Figure 2: Evaluation of ISOX therapeutic efficacy in pancreatic cancer and normal immortalized pancreatic cell lines. The effect of CMAP identified ISOX was evaluated for its sensitivity in MiaPaCa2, CD18/HPAF, and AsPC1 PC cell lines. All three PC cell lines were treated with 1nM to 100 uM concentrations (N=5) of ISOX for 24 (**A**), 48 (**B**) and 72 (**C**) hrs. (**D**) Normal immortalized pancreatic nestin-positive epithelial cells (HPNE) was treated with ISOX in a dose-dependent manner. Both PC and normal immortalized cells' response and viability against ISOX was determined using the MTT assay. Dose - response curves were generated using GraphPad Prizm. All error bars (**A-D**) are represented as mean± standard deviation (SD).

a

MiaPaCa-2

Combination index (CI) data for Non-constant Combination: 5FU+ISOX

Dose 5FU	Dose ISOX	Effect	CI
0.5	0.25	0.252	0.22854
0.5	0.5	0.32	0.13536
0.5	1.0	0.469	0.10297
0.5	1.25	0.478	0.12235
0.5	2.5	0.615	0.11269

b

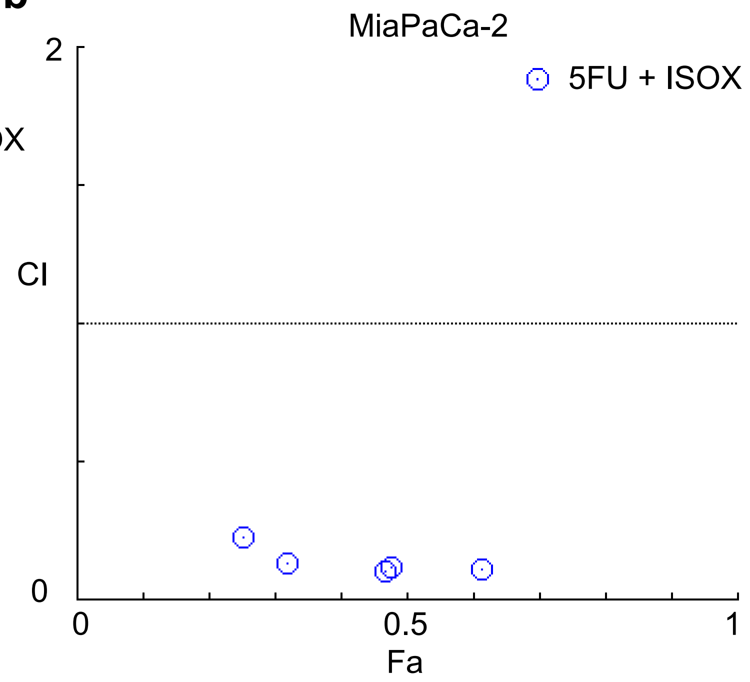

c

CD18/HPAF

Combination index (CI) data for Non-constant Combination: 5FU+ISOX

Dose 5FU	Dose ISOX	Effect	CI
0.5	0.25	0.568	0.24746
0.5	0.5	0.553	0.31496
0.5	0.75	0.54	0.39208
0.5	1.0	0.51	0.58110
0.5	2.5	0.51	0.87201

d

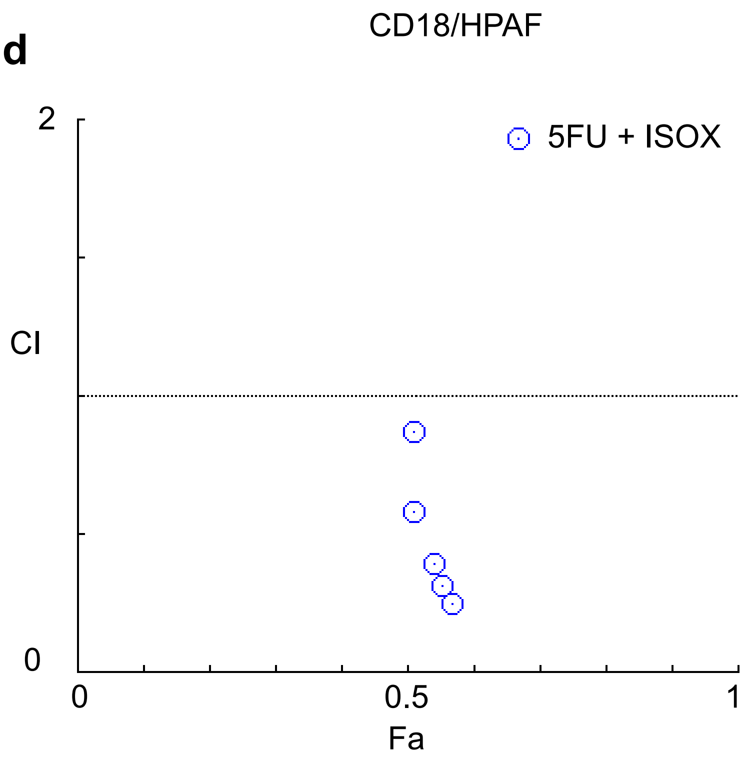

Supplementary Figure 3: ISOX exhibits a synergistic effect with 5FU in PC cells. The synergistic/additive effect of ISOX and 5FU was assessed with three different concentrations of ISOX and 5FU. The lowest concentration of 5FU (0.5 μM) was evaluated against various concentrations of ISOX (0.25-2.5 μM) in MiaPaCa2 and CD18/PAF PC cells. Values were plotted as the table for MiaPaCa2 (**A**) and CD18/HPAF (**C**), and a combination index plot (CompuSyn software) was obtained by providing input values from the MTT assay upon the drug treatment in the (**B**) MiaPaCa2 and (**D**) CD18/HPAF PC cells.

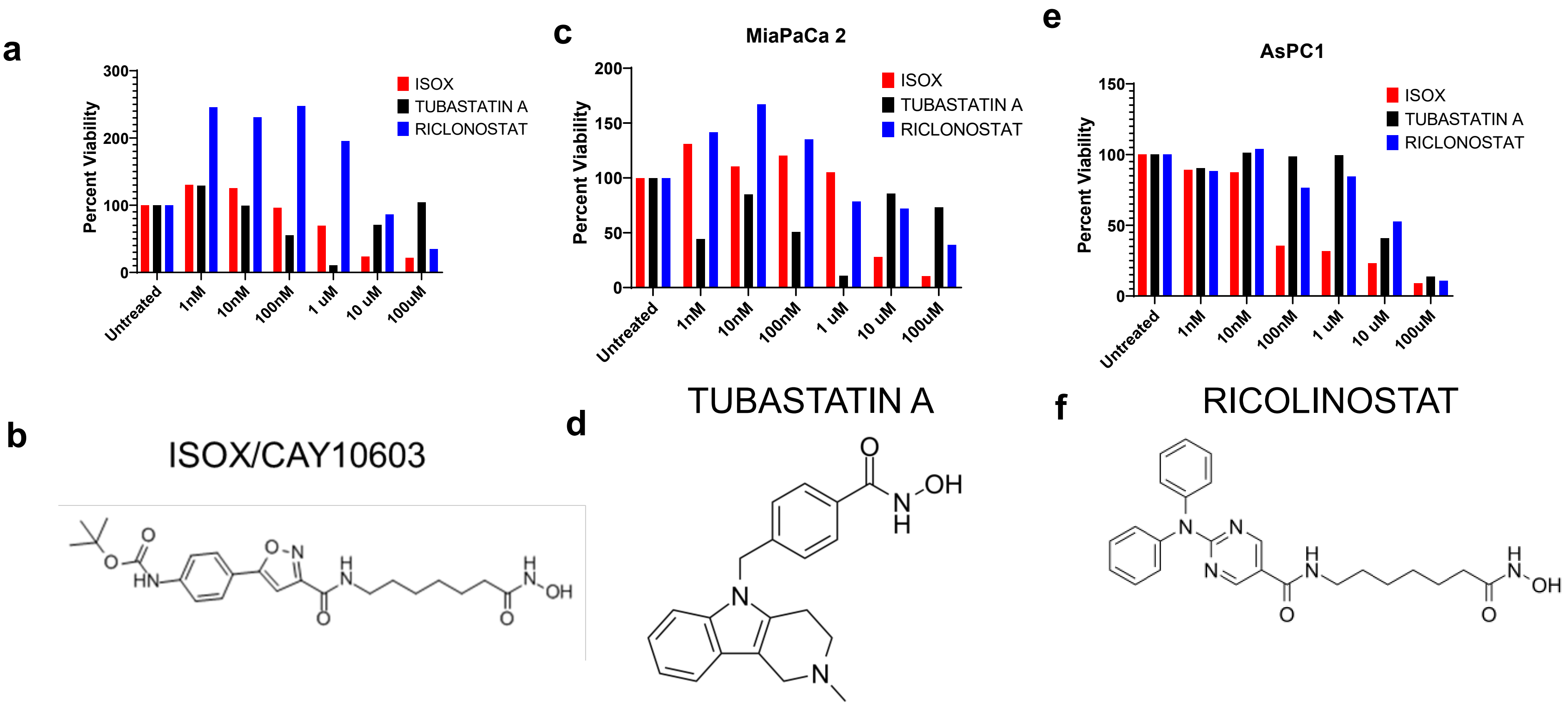

Supplementary Figure 4: ISOX inhibits the proliferation of PC cells better than other HDAC inhibitors. Head-to-head comparison of ISOX with other HDAC inhibitors. (**A-F**). The bar graph represents the percentage of viability of various PC cell lines with increasing doses (1 nM, 10 nM, 100 nM, 1 μM, 10 μM, and 100 μM) of ISOX (**A** and **B**) and two other HDAC inhibitors tubastatin A (**C** and **D**) and riclinostat (**E** and **F**). ISOX (red) was far better in reducing the viability of PC cell lines when compared to its counterparts. The viability percentage was calculated with respect to untreated to control for respective drugs.

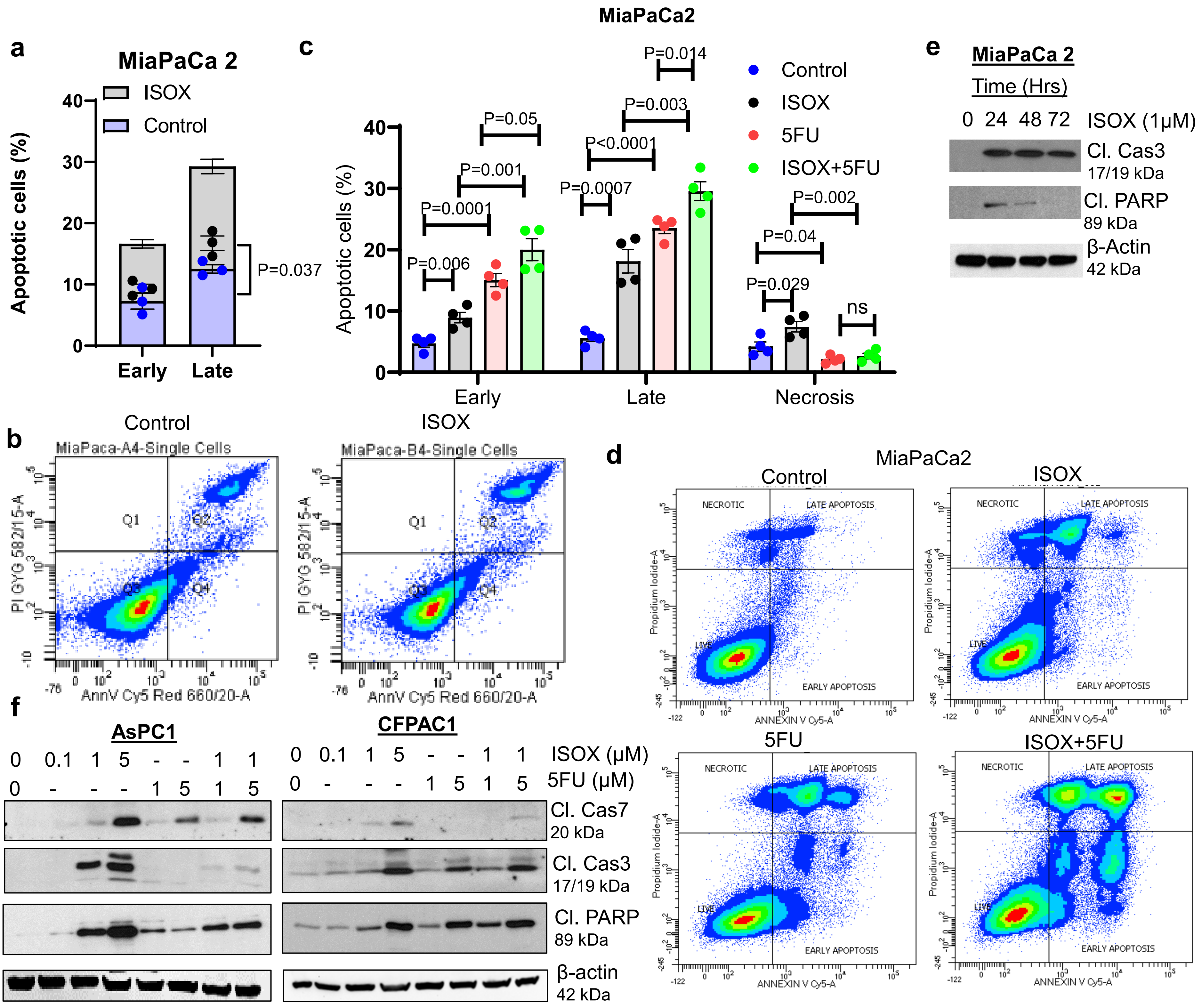

Supplementary Figure 5: ISOX induces apoptosis alone and in combination with 5FU in multiple PC cells. A and B. The dot combined bar graph, error bars represent standard deviation (s.d) of the data (**A**) and representative scatter plot from FACS (**B**) depict the number of PC cells that have undergone apoptosis under the influence of ISOX. (**C** and **D**). PC cells were serum starved for 12 hrs, followed by ISOX and 5FU treatment for 48 hrs**.** Cells were harvested and washed in PBS and stained using Annexin V-PI staining. Cells undergone apoptosis were determined using FACS analysis. ISOX and 5FU combination induces early and late apoptosis in MiaPaCa2 PC cells**.** Bar and scatter dot plots show the effect of apoptosis induction upon combination drug treatment (Error bars represent s.d) **(C).** The representative scatter plot shows the percentage of cells in each quadrant of early and late apoptosis and necrosis (**D**). (**E)** Western blotting analysis showing the effect of cleaved caspase 3 and PARP at 0, 24, 48, and 72 hrs post-ISOX treatment. (**F)** Immunoblotting analysis shows the drug treatment effects on pro-apoptotic markers Cleaved caspase 7, 3, and PARP in additional (AsPC-1 and CFPAC1) PC cells.

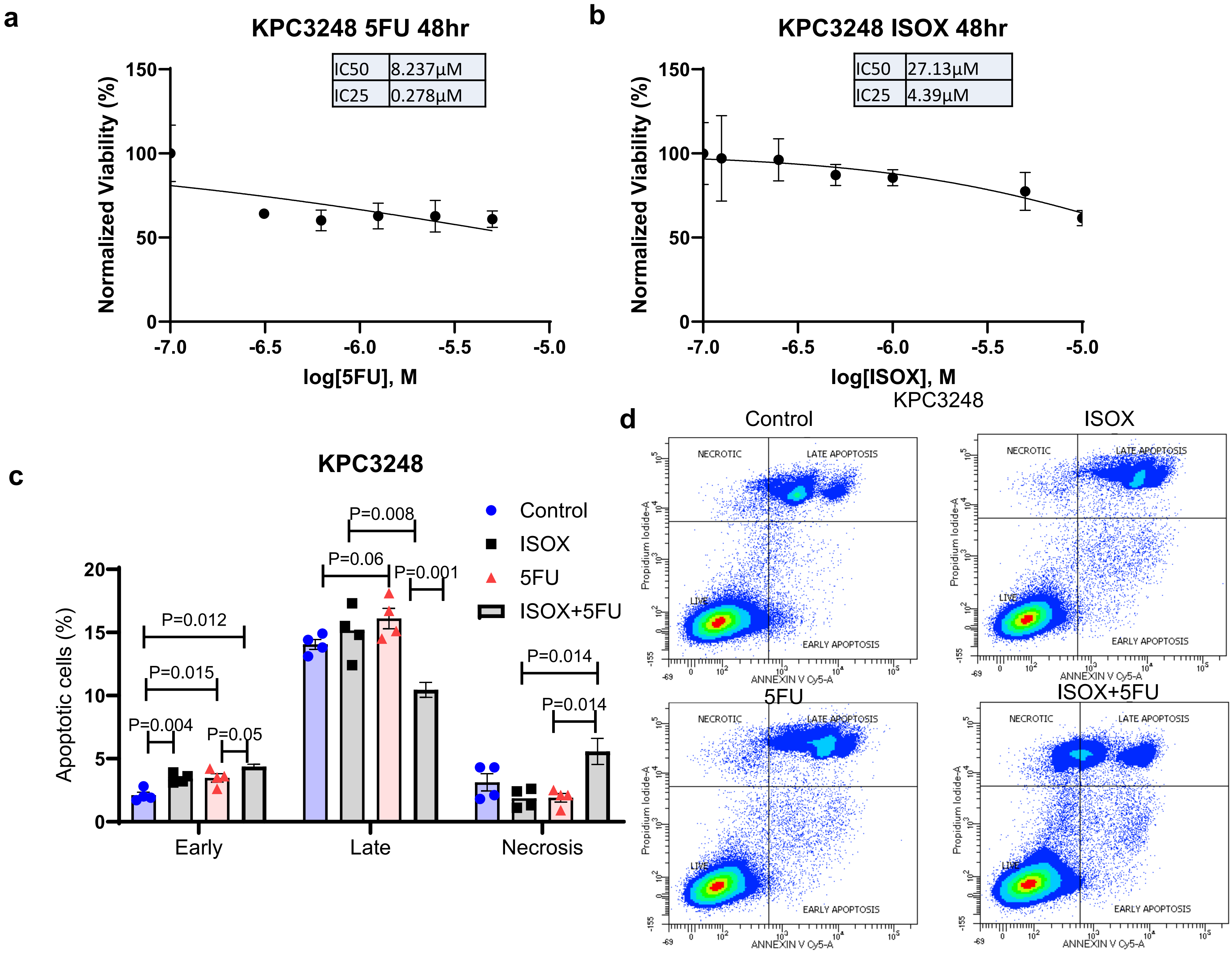

Supplementary Figure 6: ISOX and 5FU induce apoptosis in murine syngeneic cells. MTT assay was performed with various concentrations of 5FU and ISOX in KPC3248 mouse syngeneic PC cells. Line graphs show the normalized viability of KPC 3248 cells at different doses of 5FU and error bars represents s.d. (**A**) and ISOX (error bars represents s.d) (**B**). KPC 3248 cells were serum starved for 12 hrs, followed by ISOX (IC_{50}) and 5FU (IC_{25}) treatment, and further incubated for 48 hrs. PC cells were harvested, washed in PBS, and stained using Annexin V and PI. Bar with scatter dot plot demonstrates early apoptosis and necrosis induction in combination treatment compared with ISOX and 5FU alone treatment, and error bars in scatter dot plot represent s.d. (**C**). Scatter plot eliciting the percentage of cells undergoing early, late, and necrosis upon drug treatment (**D**).

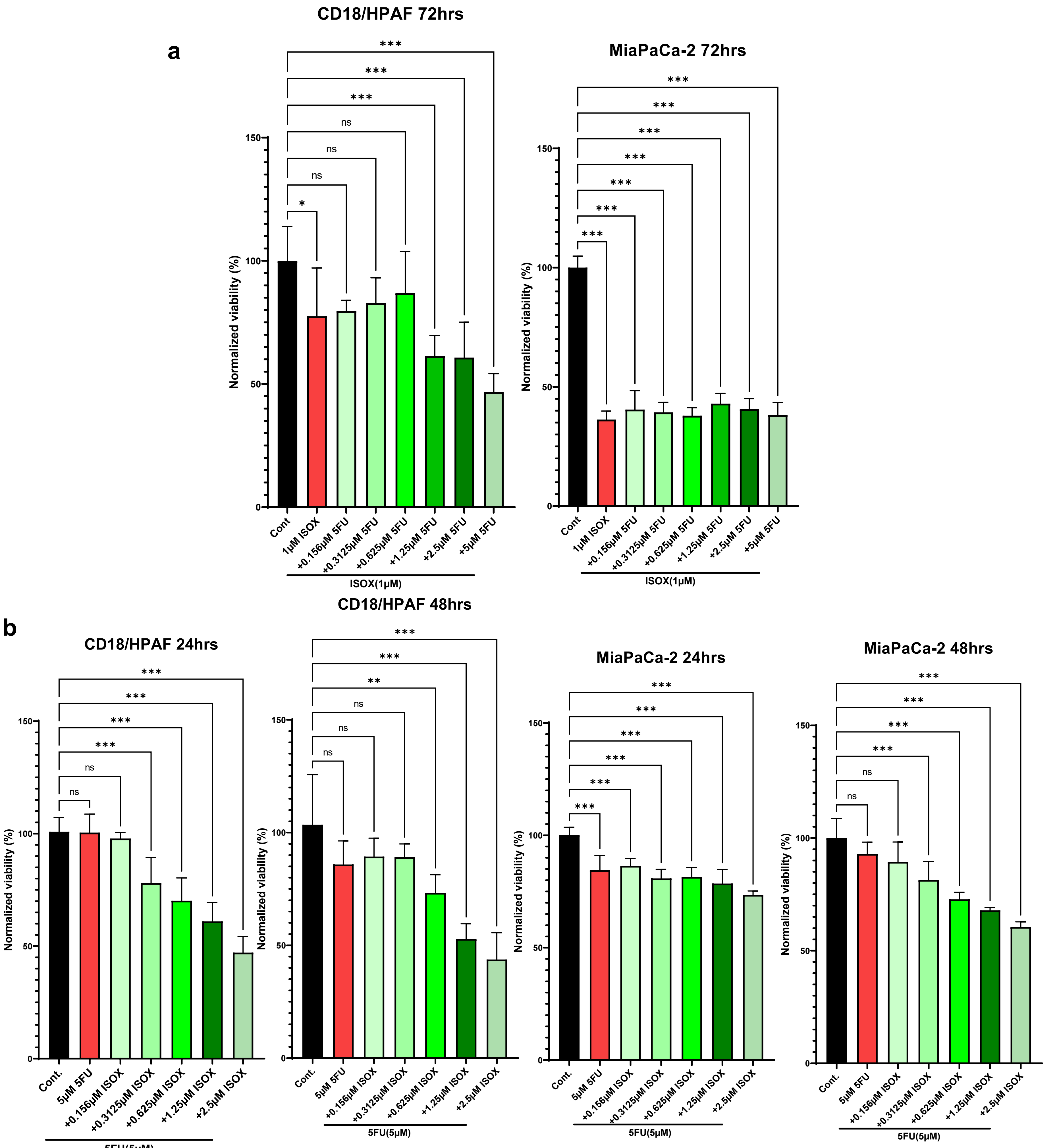

APA: P<0.12(ns), p<0.033(8), p<0.002(**), p<0.001(***)

Supplementary Figure 7: Dose-dependent treatment of ISOX and 5FU decreases the viability of PC cells. (A and B) PC cells at a density of 3000 cells/ well were seeded in 96 well plates and treated with ISOX and 5FU alone and in combination for indicated time points. The graph bars show PC cells' viability (CD18/HPAF and MiaPaCa2) upon exposure of ISOX and 5FU (dose-dependent) alone and in combination. Error bars in the bar graphs represents s.d.

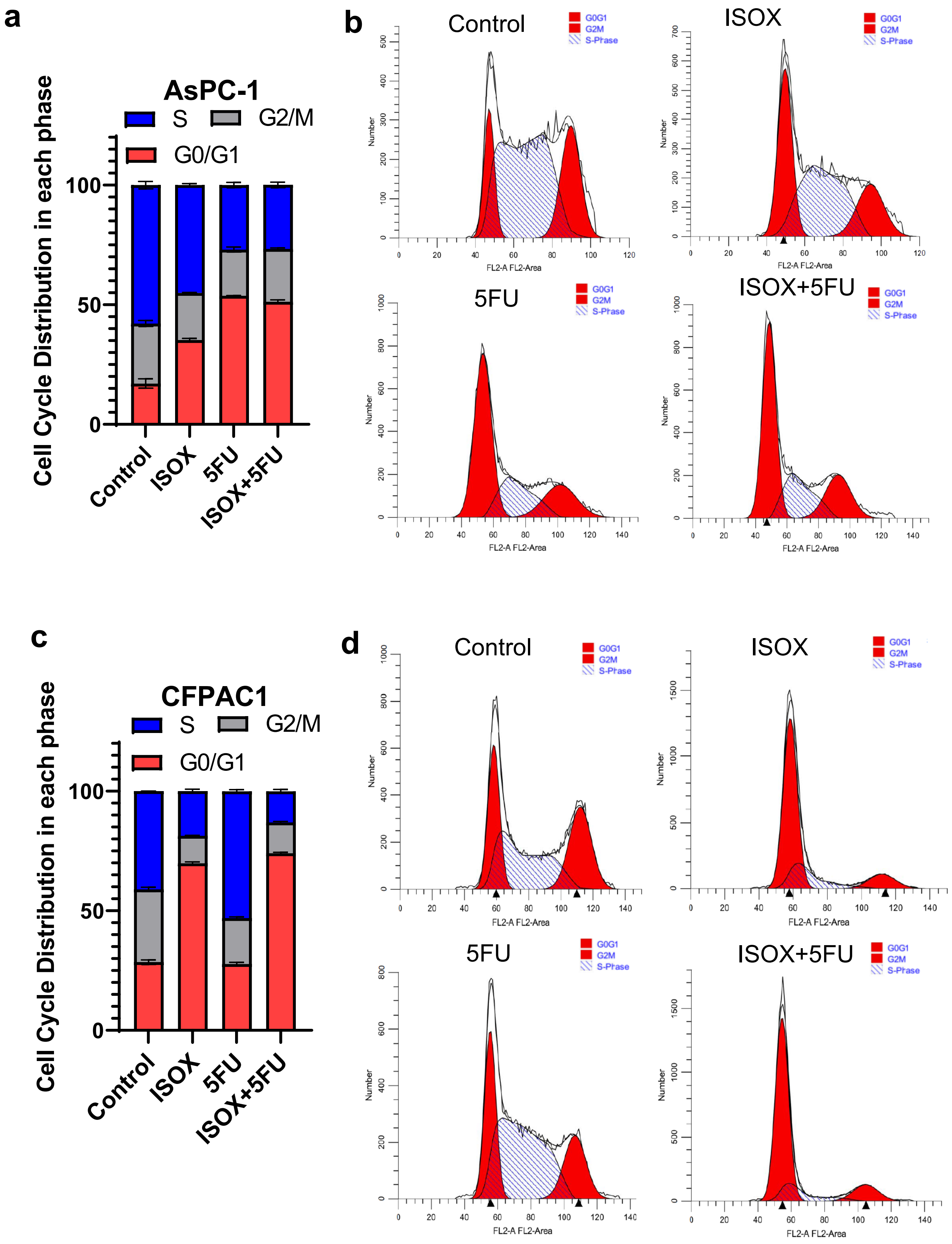

Supplementary Figure 8: Individual and combinatorial effects of ISOX and 5FU on cell cycle phases in multiple PC cells. (**A** and **C**). The stacked bar graph represents the accumulation of cells in each cell cycle phases upon ISOX and 5FU treatment in AsPC-1 and CFPAC1 PC cells, error bars represent s.d. (**B** and **D**). Flow cytometry-based population histogram for AsPC-1 and CFPAC1 PC cells treated with ISOX and /or 5FU.

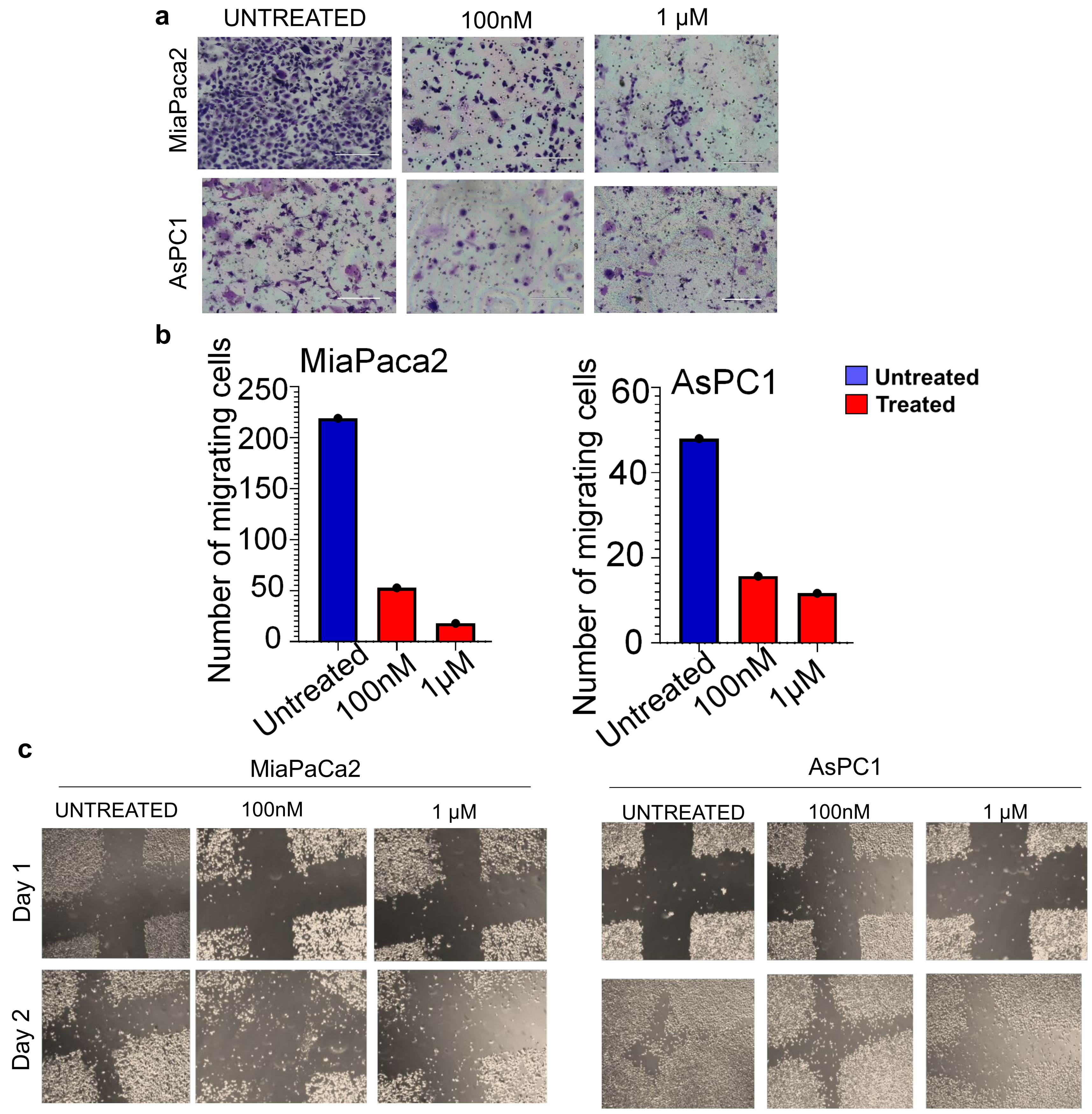

Supplementary Figure 9: ISOX treatment affects the migration and wound-healing ability of PC cells. **A** and **B** Representative transwell images showing the dose-dependent (100 nM and 1 uM concentrations) treatment effects of ISOX preventing migration of PC cells (MiaPaCa-2 and AsPC1) across matrigel barrier. The bar in the images represents 100 µm (**A**). Bar graphs quantitating the number of cells migrated under dose-dependent (100 nM and 1 uM concentrations) treatment of ISOX in MiaPaCa-2 and AsPC1 PC cells (**B**). **C**. Representative light microscopic images showing wound closure effects of 100 nM and 1 uM concentrations of ISOX relative to untreated controls in MiaPaCa-2 and AsPC1 PC cells. Images were taken at 100X magnification (objective lens (10X) x eyepieces (10X)).

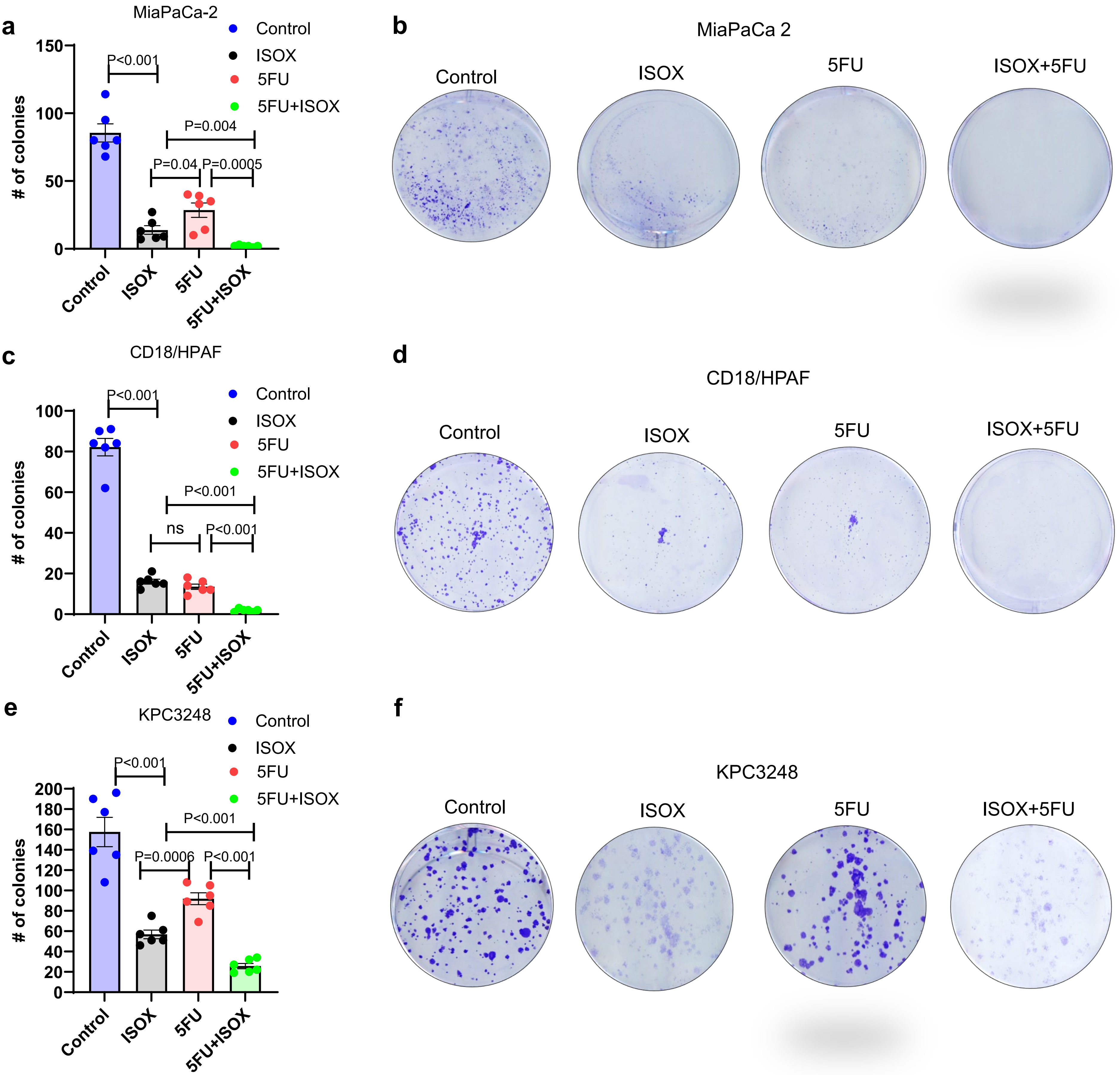

Supplementary Figure 10: ISOX and 5FU suppress colony growth of human and mouse PC cells. Both human and mouse PC cells (250 cells/well) were seeded in a 10% complete DMEM medium and treated with ISOX and 5FU after 24 hrs. After 48 hours of drug treatment, cells were allowed to grow and form colonies for 10-14 days. Cells were fixed, stained with crystal violet, and counted. Quantification of the substantial number of colonies formed against ±drug treatment in MiaPaCa2 (**A**), CD18/HPAF (**C**), and KPC3248 (**E**) were plotted as bar and dot plots. Error bars represent s.d. Representative light microscopic images of colonies formed against ISOX, 5FU, combination, and no treatment were presented: MiaPaCa2 (**B**), CD18/HPAF (**D**), and KPC3248 (**F**).

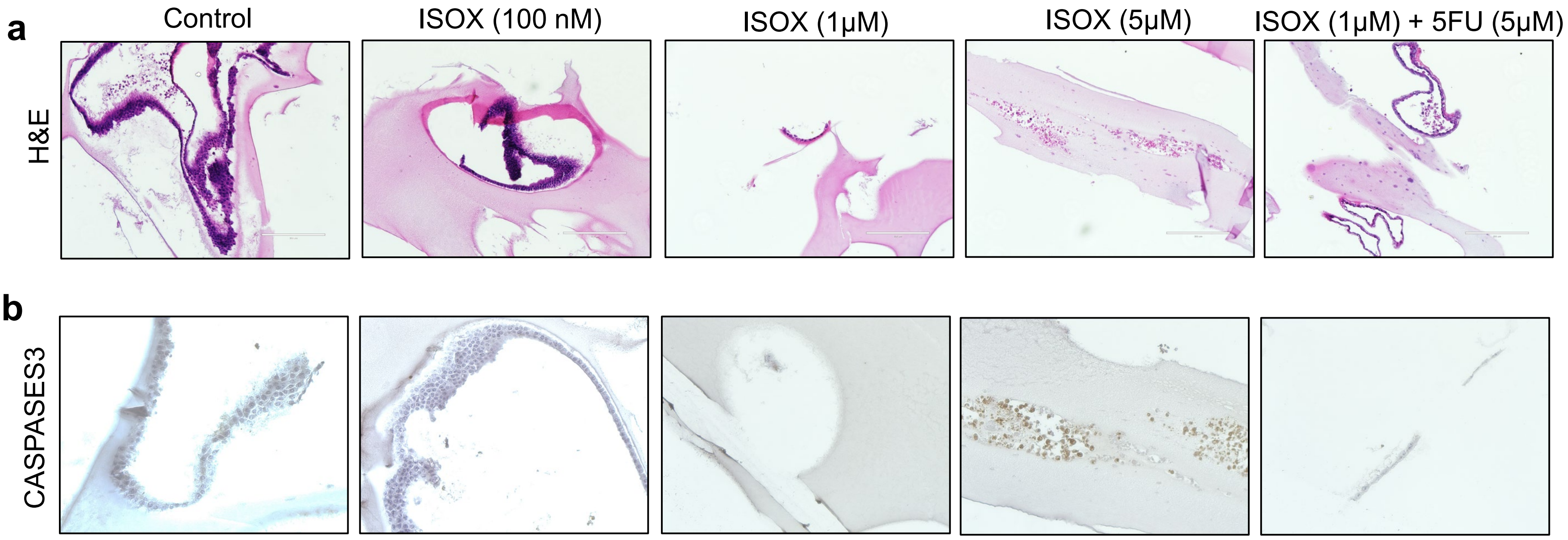

Supplementary Figure 11: Histological and biochemical changes on human pancreatic cancer patient-derived tumoroids treated with ISOX and 5FU as single and combination therapy. Panel of microscopic images of hematoxylin and eosin staining of PDAC-tumoroid treated with various dose of ISOX (100nM, 1 μM, and 5 μM) as a single and combined with 5FU, the magnification bar denotes 200 μm. (**A**) and caspase 3 (Magnification at 400X (objective lens (40X) x eyepieces (10X)) (**B**) immunohistochemical staining images illustrating the single and combinatorial effects of ISOX and 5FU in human PC tumoroids.

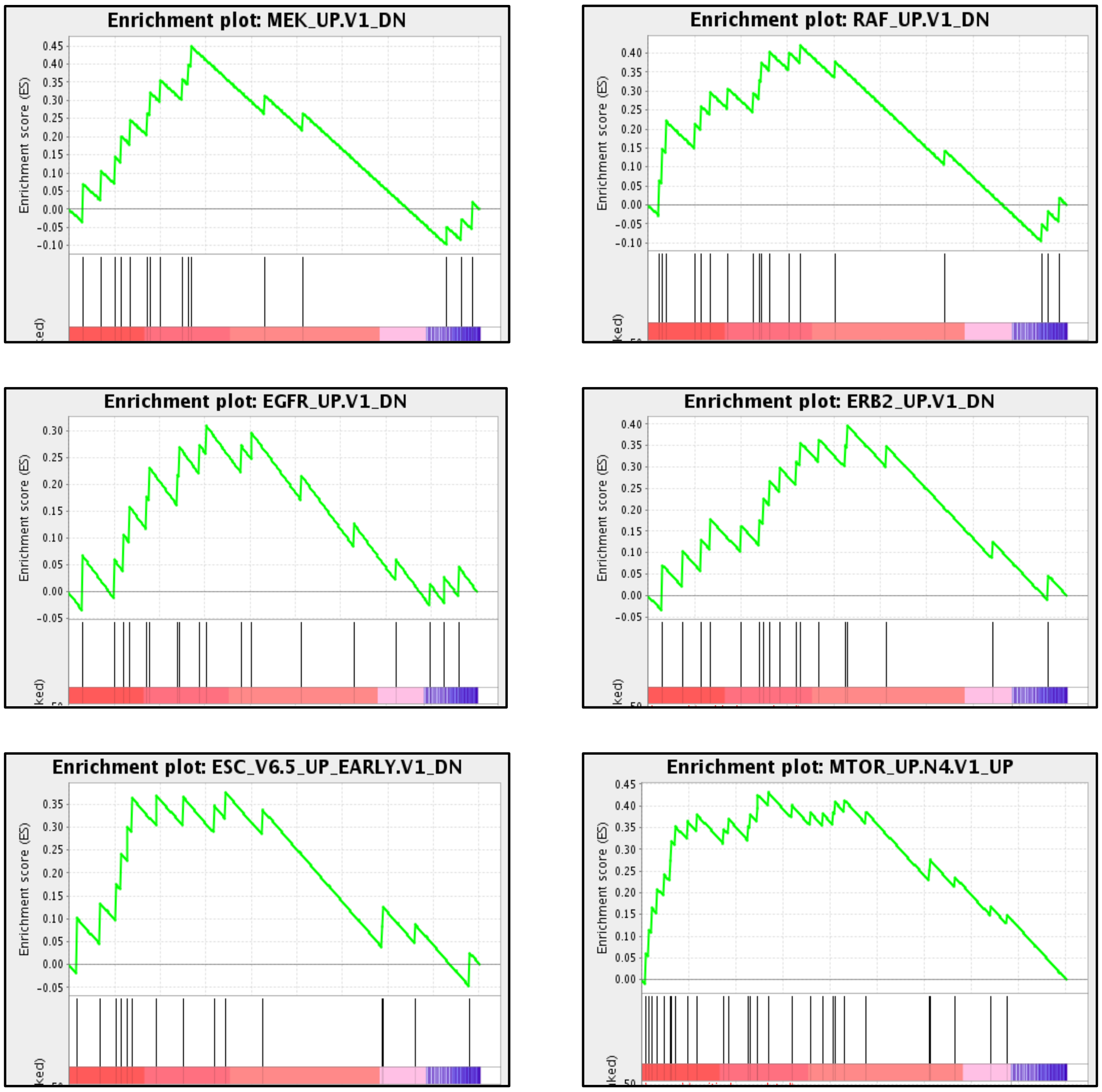

Supplementary Figure 12: Gene set enrichment analysis of ISOX-related pathways/proteins Gene set enrichment comparing the MEK, RAF, EGFR, ERB2, embryonic stem cell, and mTOR pathway-related genes between control (red) and ISOX (blue) treatment in PC cells.

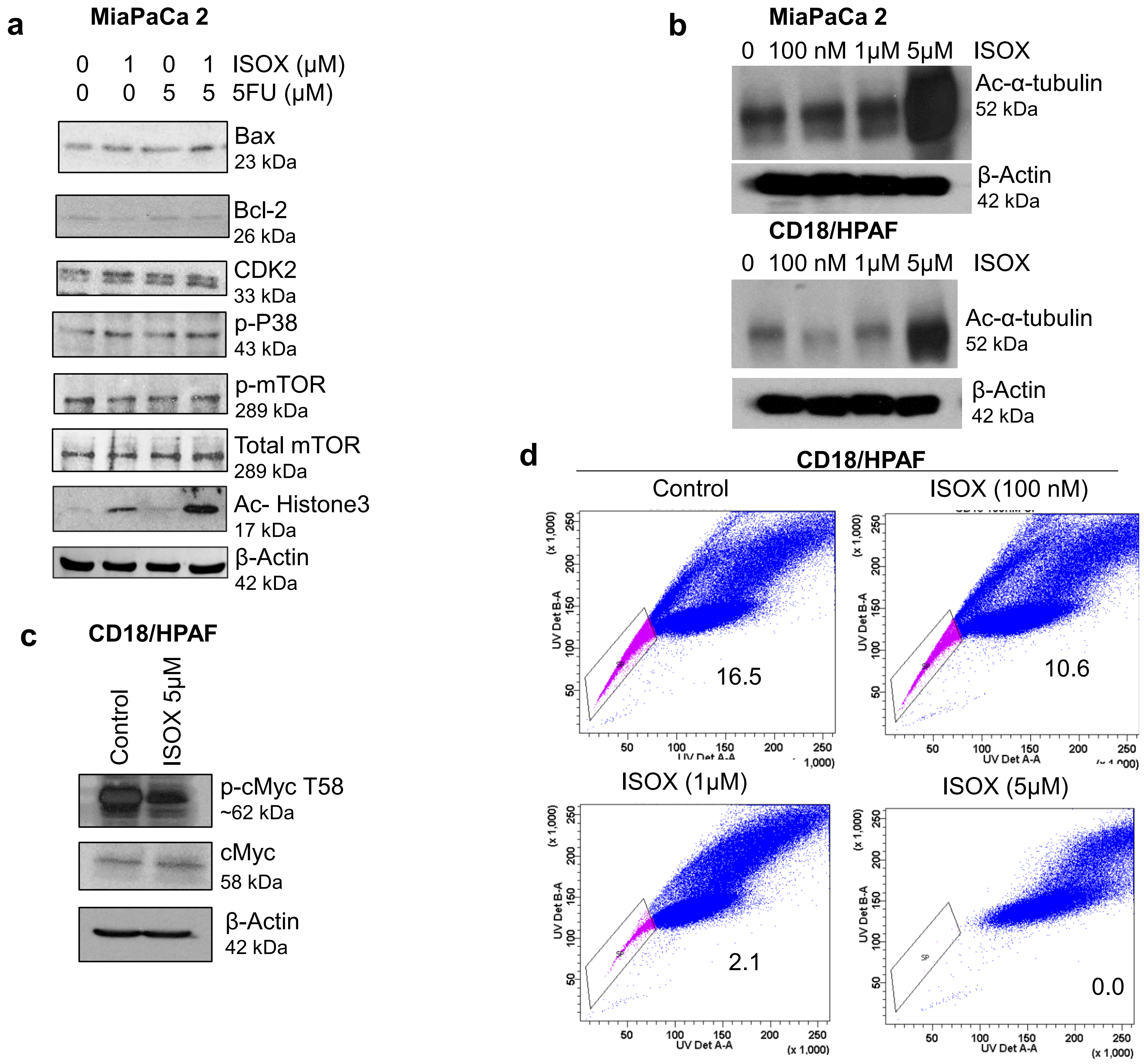

Supplementary Figure 13: ISOX treatment in PC cells affects pro-apoptotic, epigenetic, and oncogenic signaling, reducing cancer stemness. (**A -C)**. Western blot analysis showing the effect of combinatorial treatment of ISOX and 5-FU on apoptosis-related proteins (Bax, Bcl-2), cell cycle-related protein (CDK2), oncogenic signaling protein (phospho 38, phospho mTOR, phospho cMyc), epigenetic marker (acetylated histone 3) and post-translation modification of acetylated-α-tubulin in CD18/HPAF and MiaPaCa-2 cells. **(D)** Dose-dependent treatment of ISOX affecting side populations detected using flow cytometry-based Hoechst dye analysis.

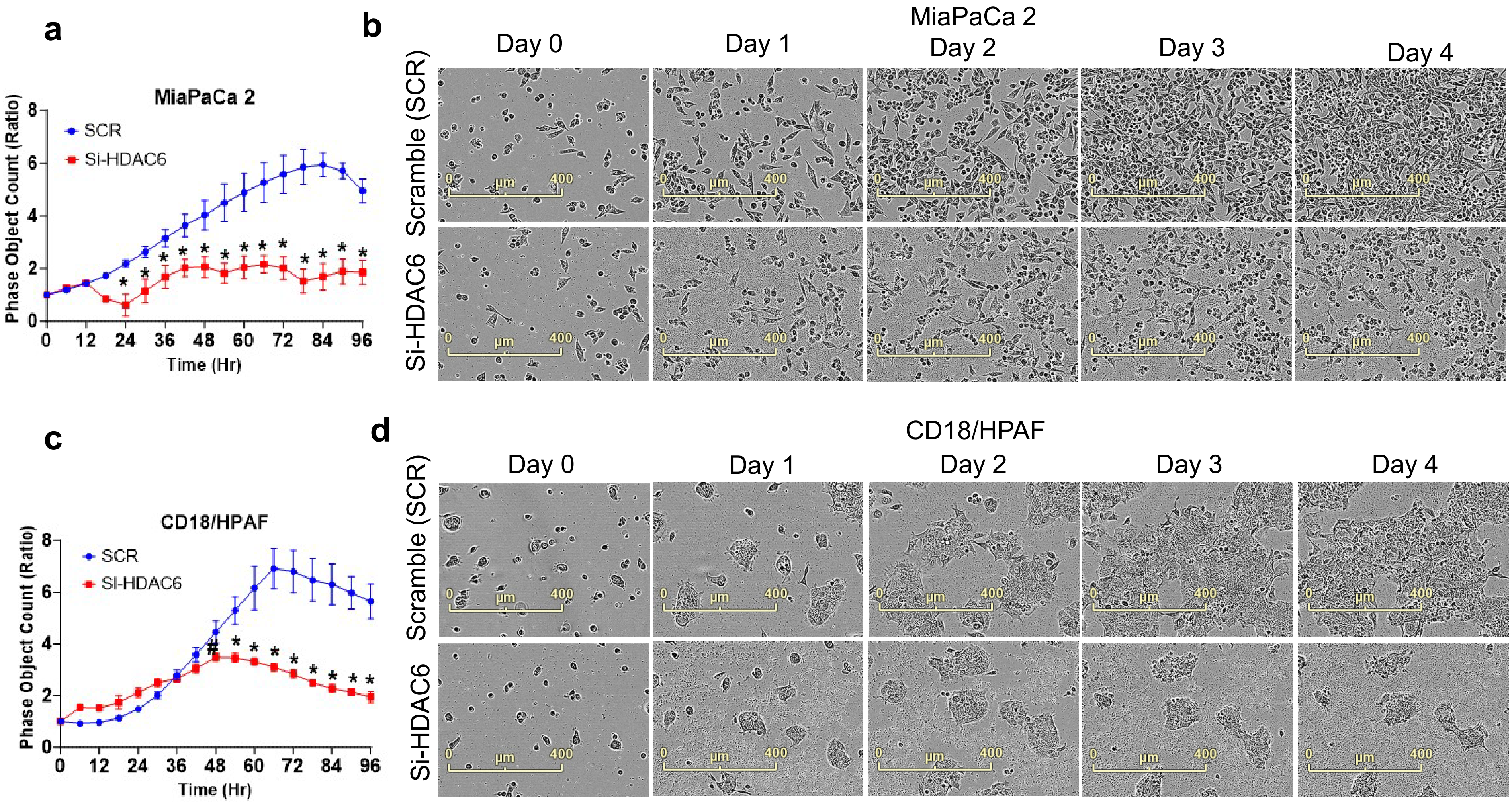

Supplementary Figure 14: Genetic inhibition of HDAC6 affects the viability/growth of PC cells. PC cells were treated with siRNA specific for HDAC6 and scramble vector and followed up to 72 hrs after transfection. The cells were followed for growth response in real-time using an Incucyte live imaging system for up to 96 hours from cell seeding. The line graph shows a significant decrease in proliferation ability and growth of MiaPaCa2 (**A**) and CD18/HPAF (**C**) PC cells. Representative micrographs of cell's response against HDAC6-siRNA and scramble vector control upon MiaPaCa2 (**B**) and CD18/HPAF (**D**) PC cells. The magnification bars in the representative images represents 400 μm. Error bars in the line graph represent s.d.

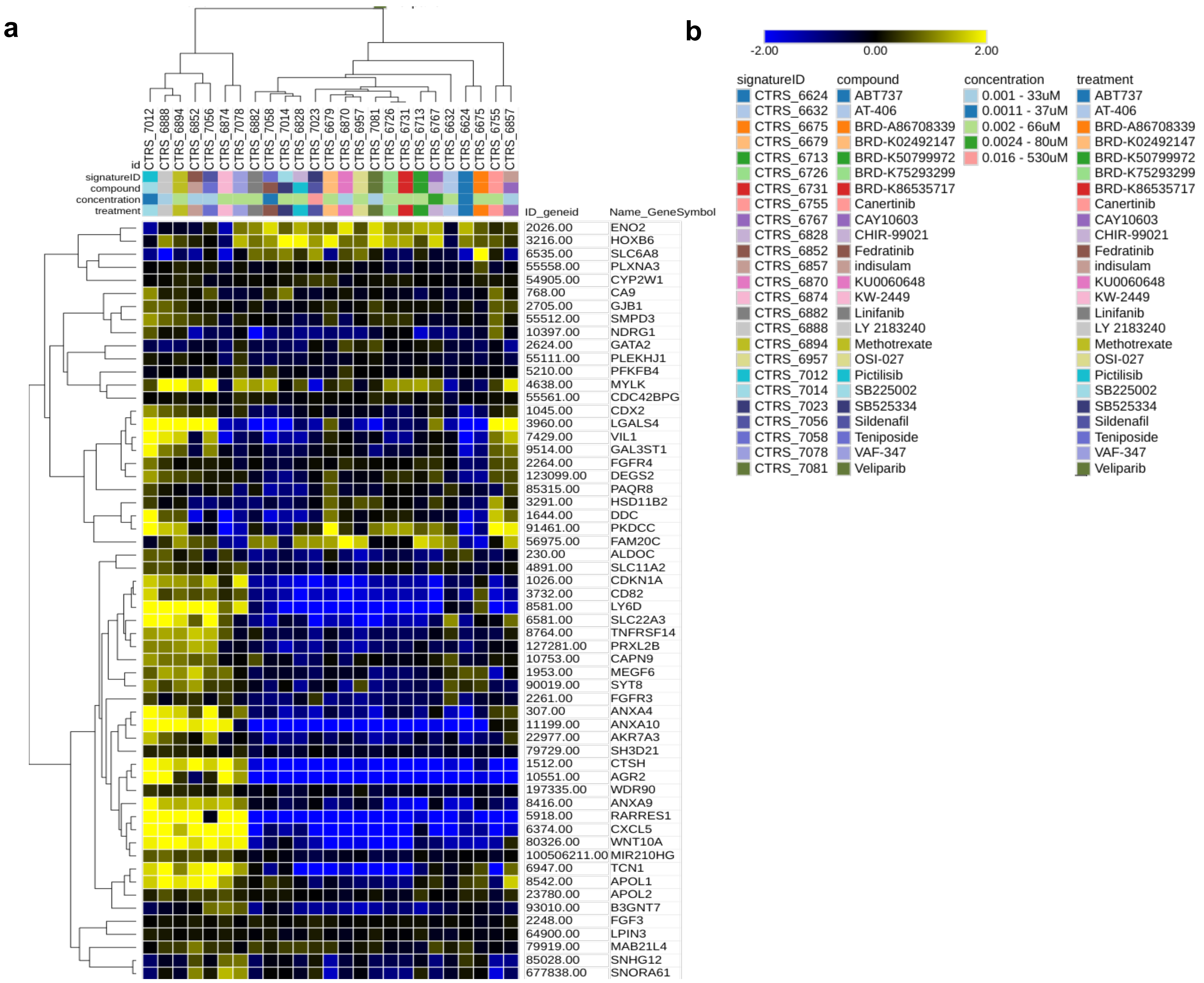

Supplementary Figure 15: Heat map representation of ISOX gene signature in conjunction with the other related therapeutics. Genes that responded to different compounds was provided as a heat map. Genes that are directly or indirectly associated to MYC pathway in cancer were identified (**A**). Signature IDs of S15A and respective compound name/drug name is briefly elaborated (**B**).

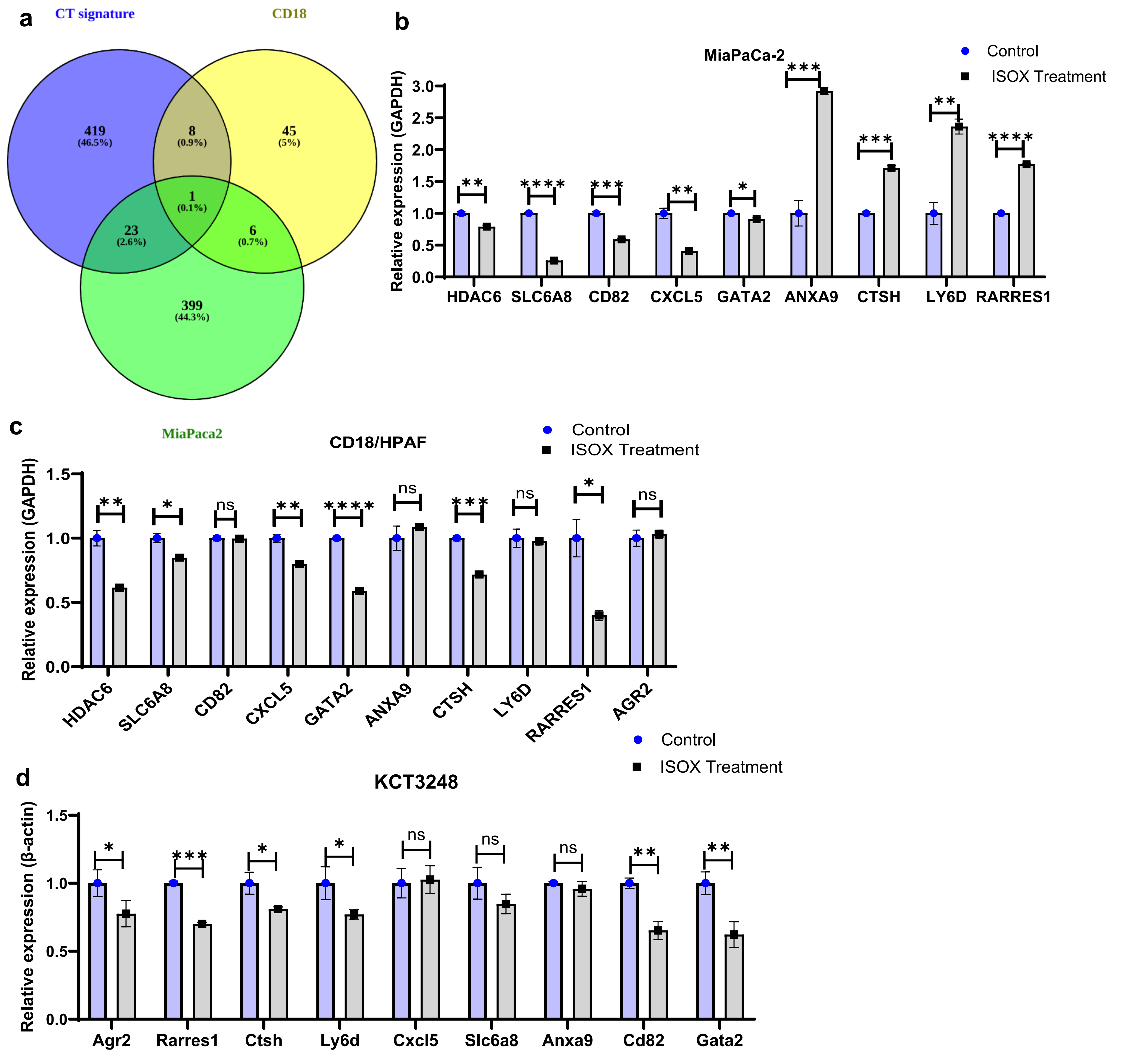

Supplementary Figure 16: Consensus between gene signatures of different transcriptomic data sets identifies commonly downregulated genes by ISOX. The Venn diagram compares the consensus between CT signature and genes downregulated in CD18/HPAF and MiaPaCa2 cells by ISOX treatment (**A**). The analysis identified 9 commonly affected genes in all three settings, and GATA2 is the most common gene predicted to be downregulated in all datasets. B-D Validation of differentially downregulated genes in response to ISOX and other HDAC6. The bar graph represents qPCR analysis of commonly affected 9 genes in human (MiaPaCa2 (**B**) and CD18/HPAF (**C**)) and mouse (KPC3248 (**D**)) PC cells. GATA2 is downregulated in both human and mouse PC cells. Error bars in the bar graphs represent s.d.

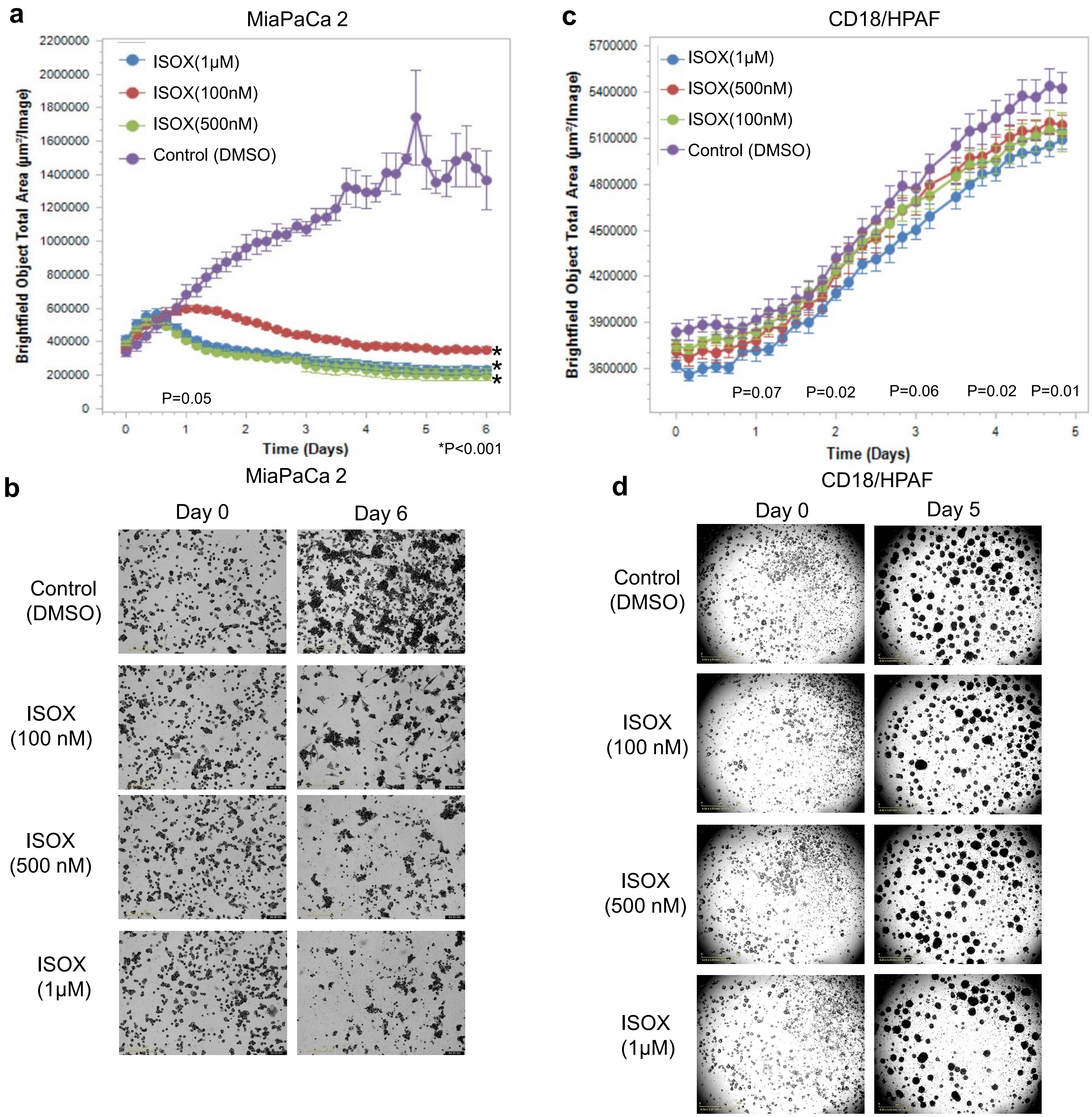

Supplementary Figure 17: Effect of ISOX on MiaPaca-2 and CD18/HPAF spheroids growth. The line graph of MiaPaCa2 (**A**) and CD18/HPAF (**C**) shows the changes in the spheroid growth as monitored using an incucyte live imaging system, and data analyzed using incucyte essence software and plotted as total area (μm2/image) to each treatment condition. The experiment was performed in sextuples for 5-6 days, and DMSO was used as vehicle control. Brightfield images of MiaPaCa2 (The magnification bar in the images represents 400 μm) (**B**) and CD18/HPAF (The magnification bar in the images represents 800 μm) (**D**) spheroids at 0 and 6 days of treatment with ISOX at 100nM, 500nM, and 1μM, captured on Incucyte live imaging system SX3. Error bars in the line graph represent s.d.

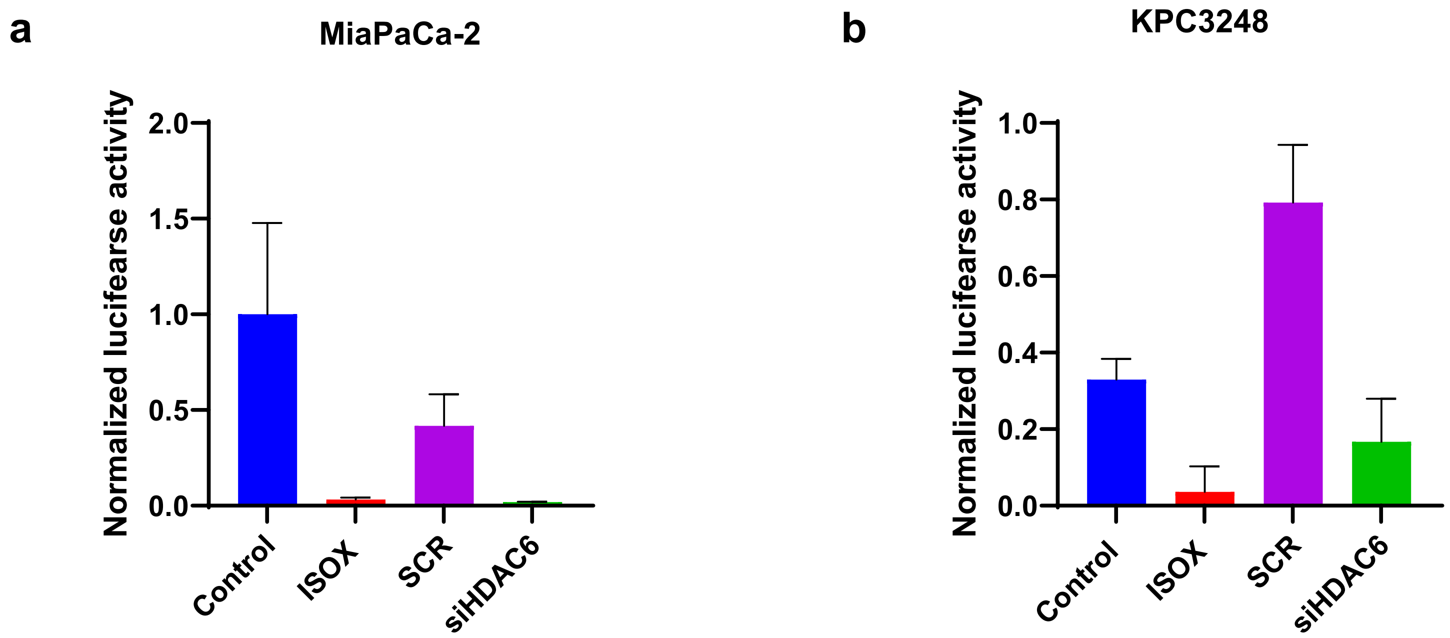

Supplementary Figure 18: ISOX affecting WNT-β-catenin signaling. Wnt/β-catenin pathway activity in MiaPaCa-2 and KCT3248 cells was measured 24 hours post-treatment with ISOX (500nM). The experiment used a TCF/LEF reporter kit and followed standard manufacturer instructions. The assay was performed in quadruplicates. The luciferase activity was normalized for untreated cells and negative vector control. The bar graph demonstrates a decrease in luciferase activity upon ISOX treatment in human MiaPaCa2 (**A**) and murine (KPC3248) (**B**) PC cells. HDAC6-specific siRNA also exhibited similar results in controlling WNT- β-catenin signaling in PC cells (**A** and **B**). Error bars in the bar graph represent standard error of the mean (s.e.m).

Supplementary Figure 19: Uncropped scans of the most important blots in Figure 1 and 2

Figure 1g

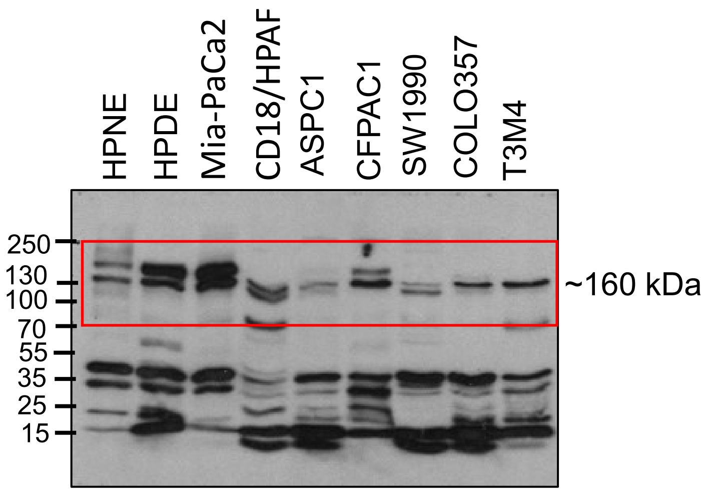

HDAC 6

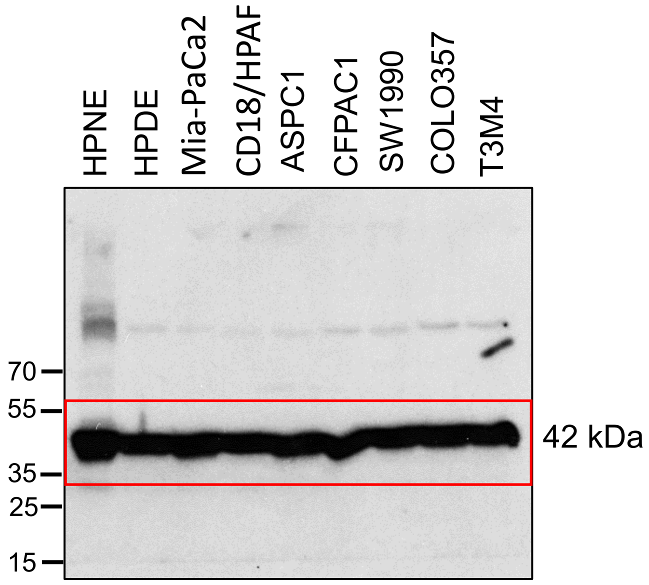

β-actin

Figure 2c

CD18/HPAF

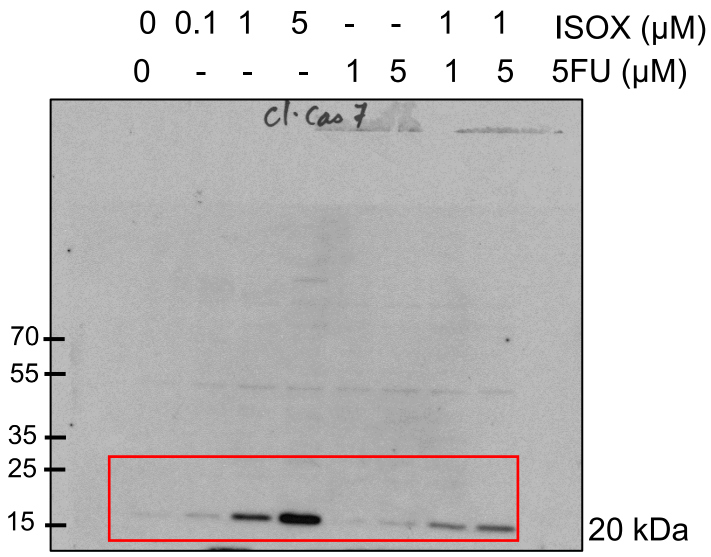

Cl.Caspase7

CD18/HPAF

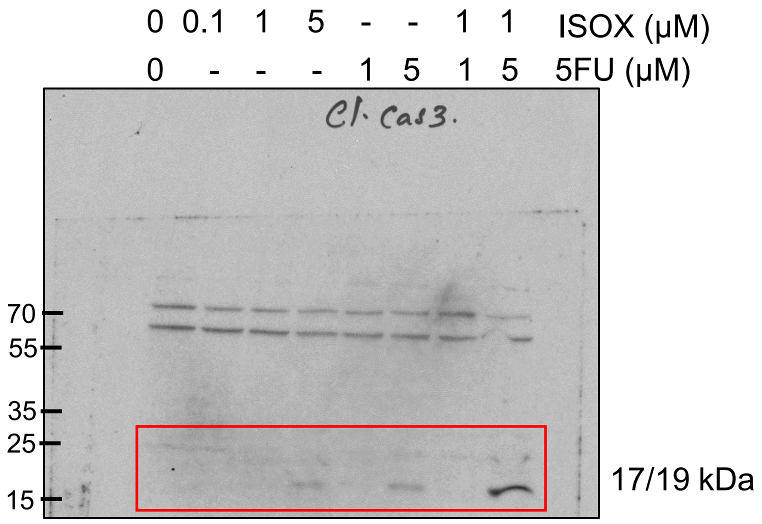

Cl. Caspase3

CD18/HPAF

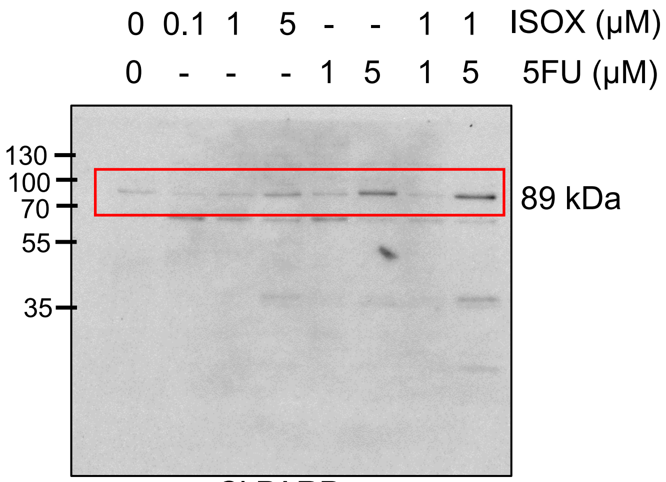

Cl.PARP

CD18/HPAF

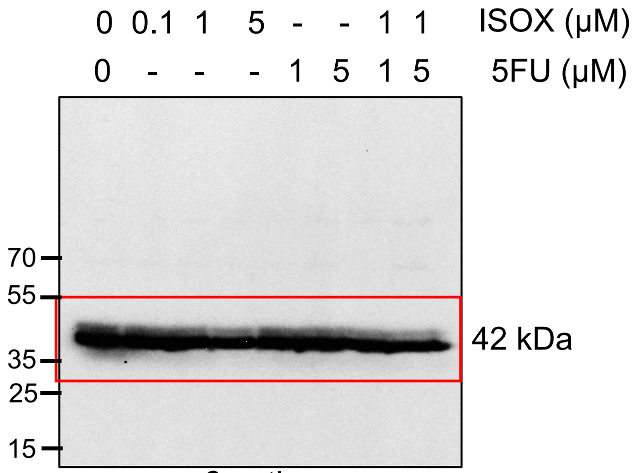

β-actin

Supplementary Figure 20: Uncropped scans of the most important blots in Figure 2

Figure 2c

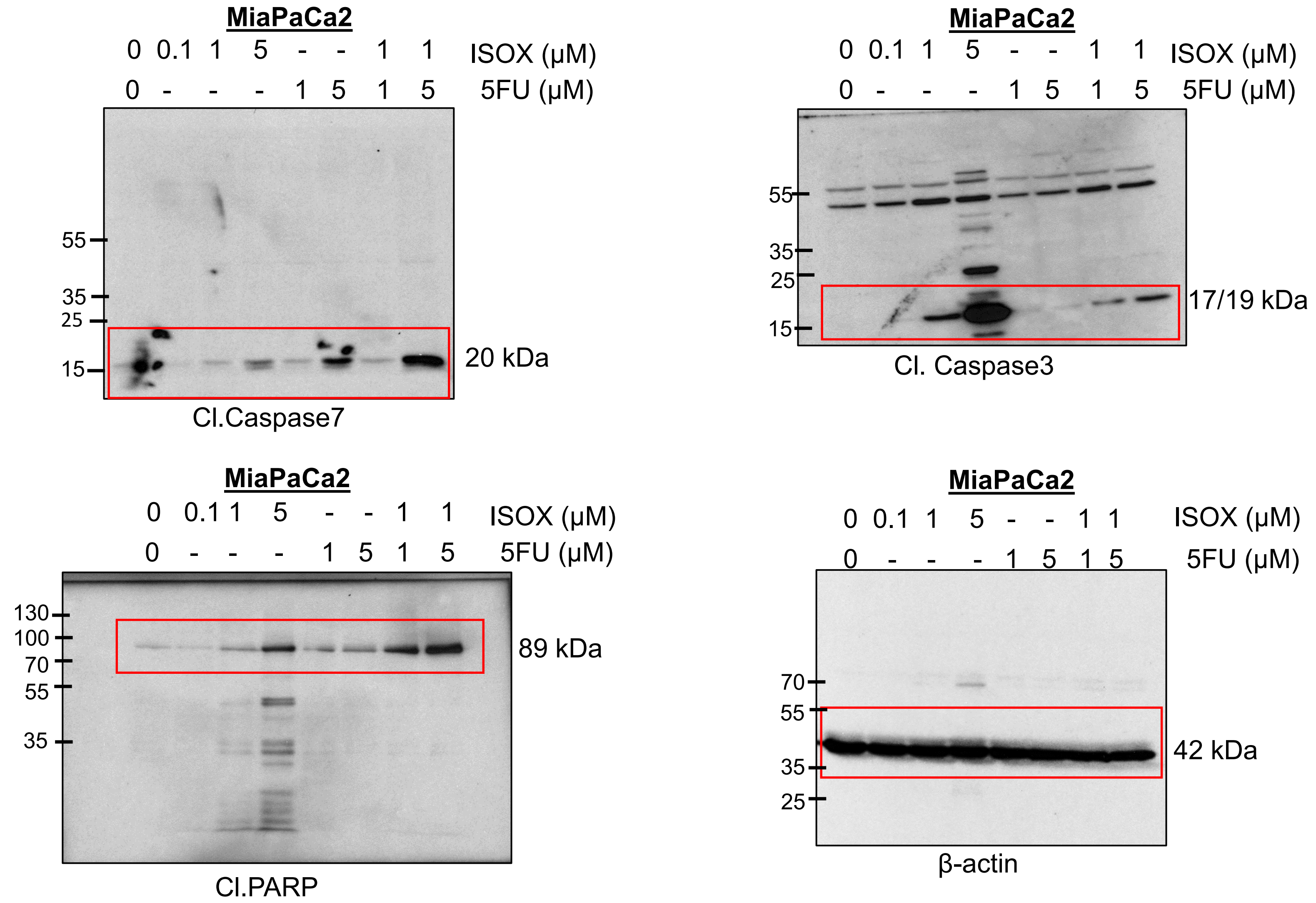

Figure 2d

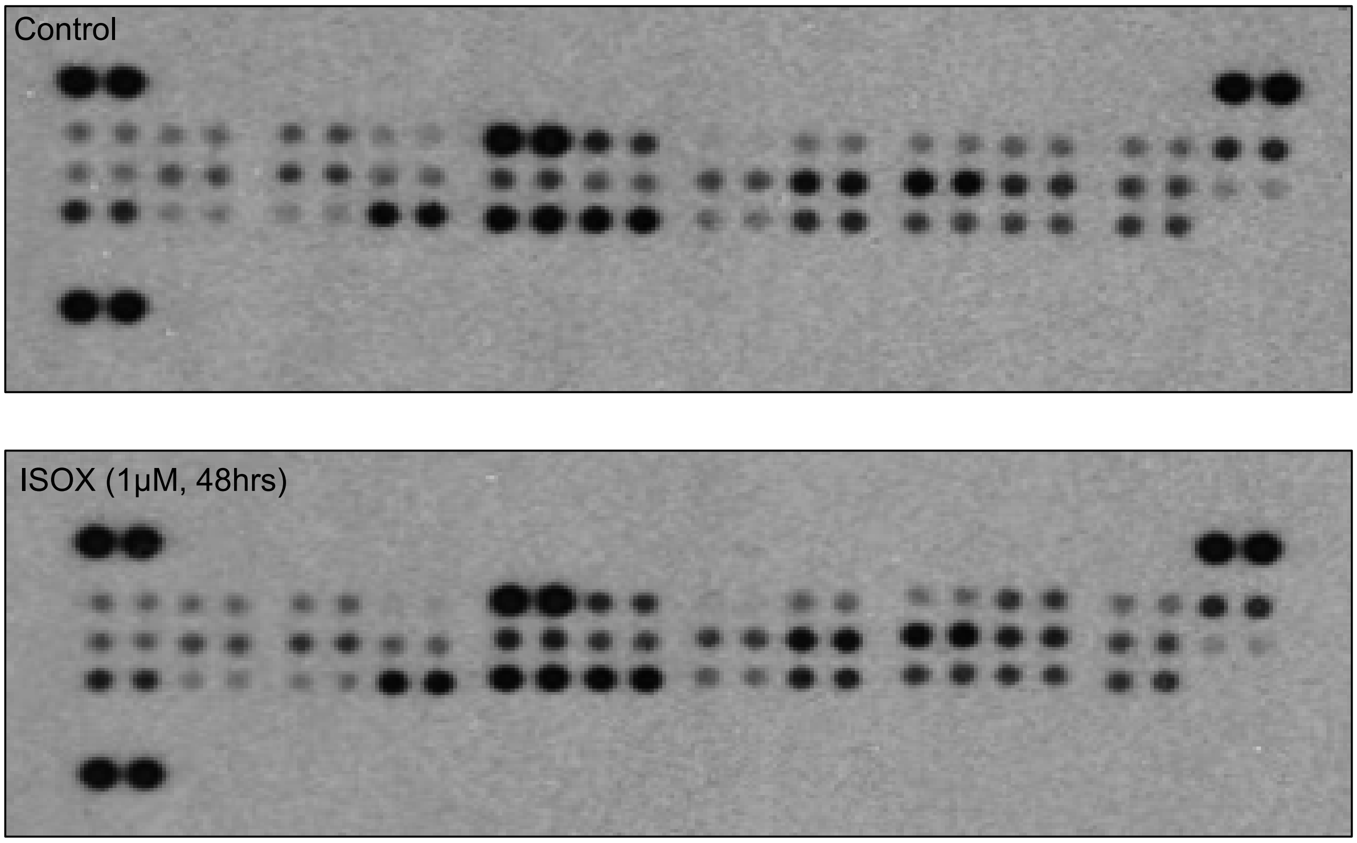

Supplementary Figure 21: Uncropped scans of the most important blots in Figure 5

Figure 5d

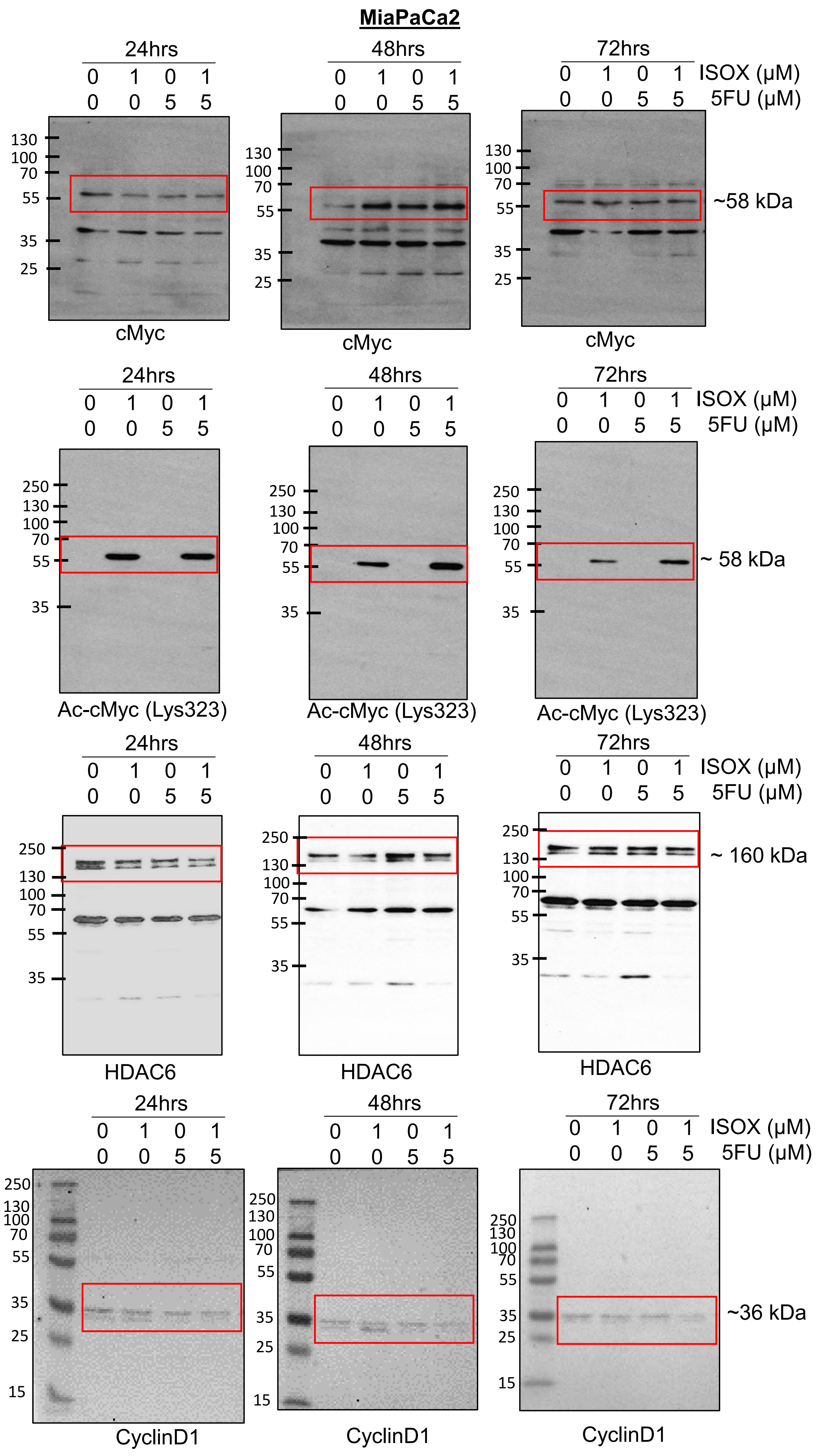

Supplementary Figure 22: Uncropped scans of the most important blots in Figure 5

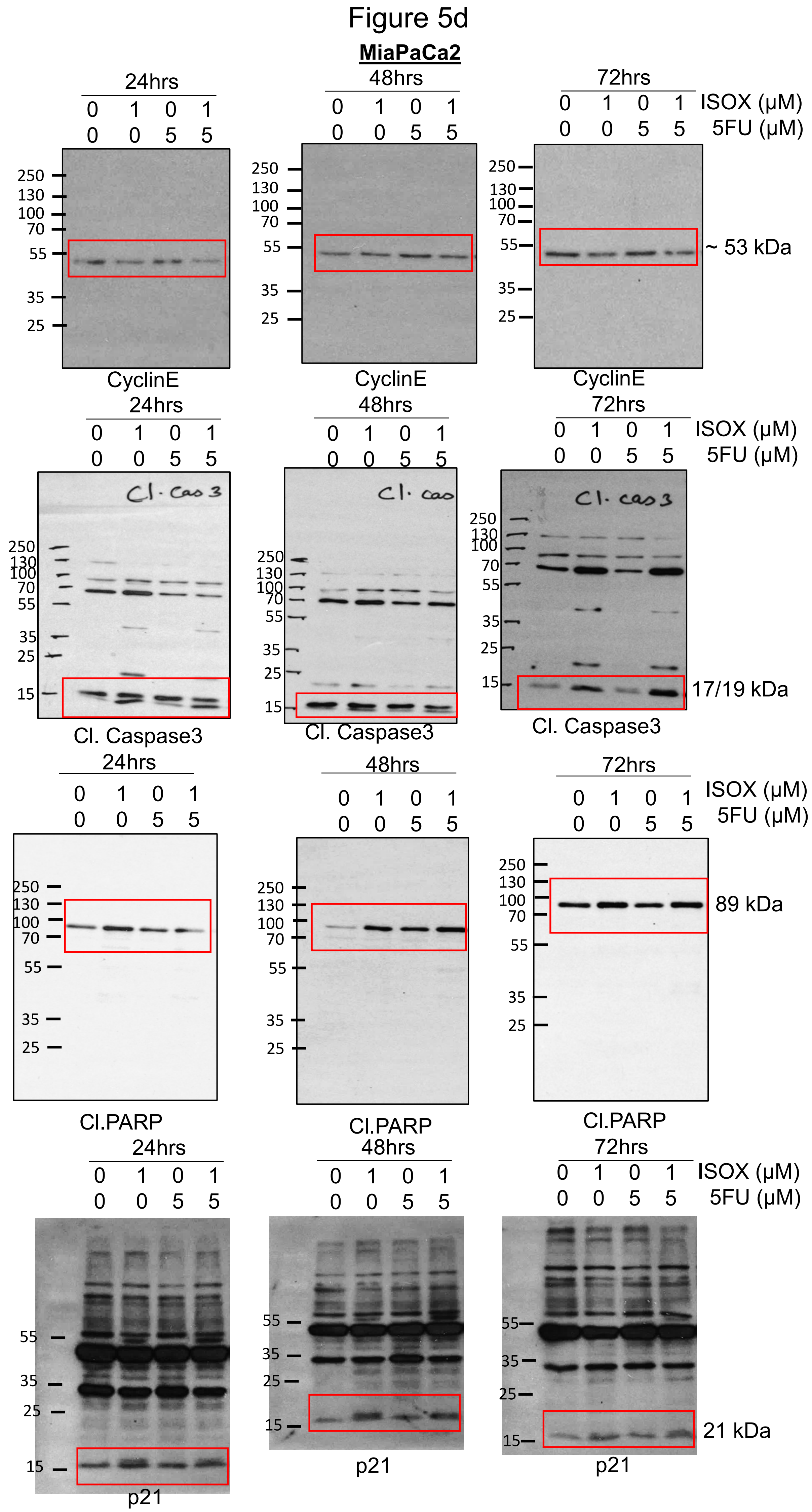

Supplementary Figure 23: Uncropped scans of the most important blots in Figure 5

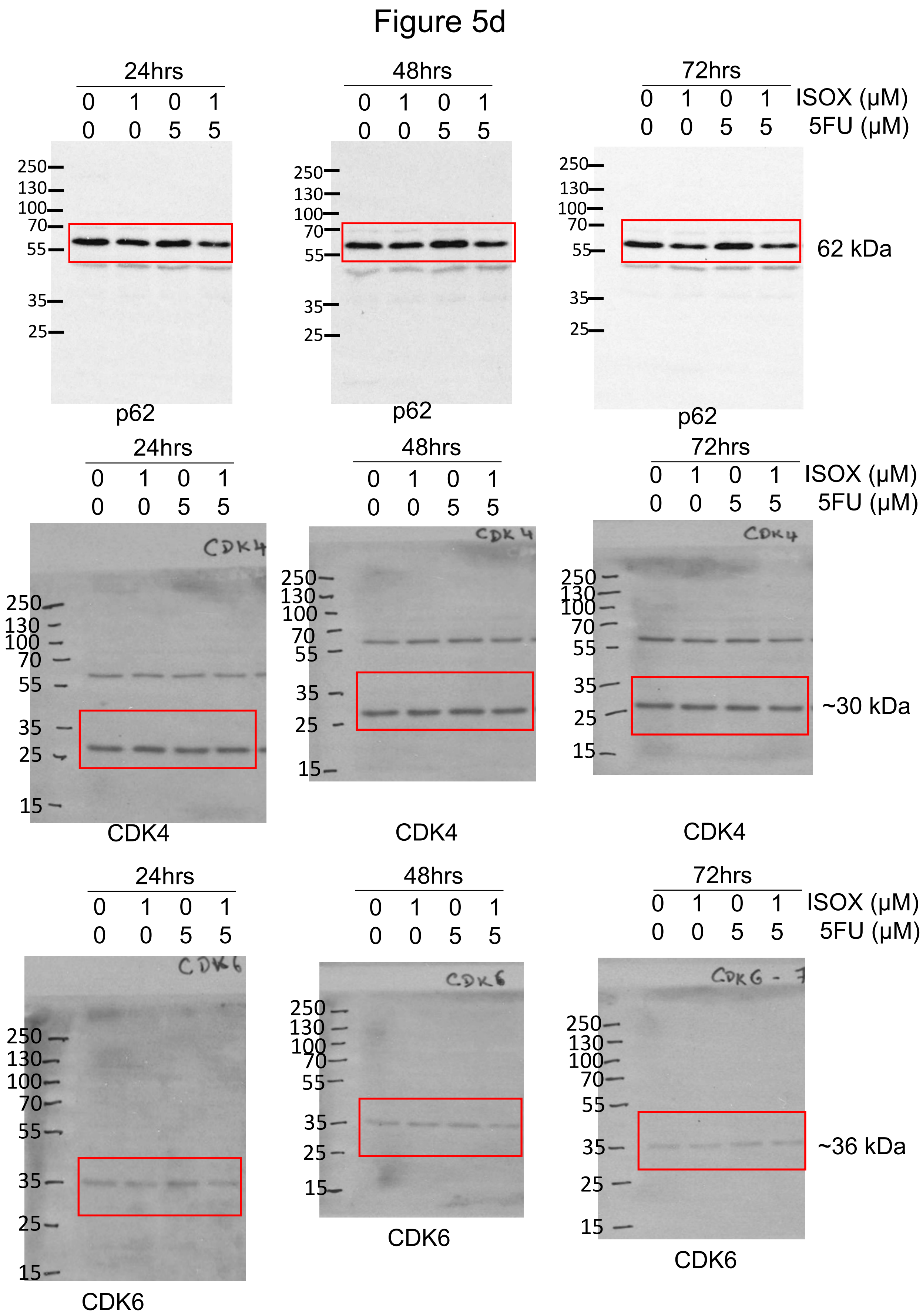

Supplementary Figure 24: Uncropped scans of the most important blots in Figure 5

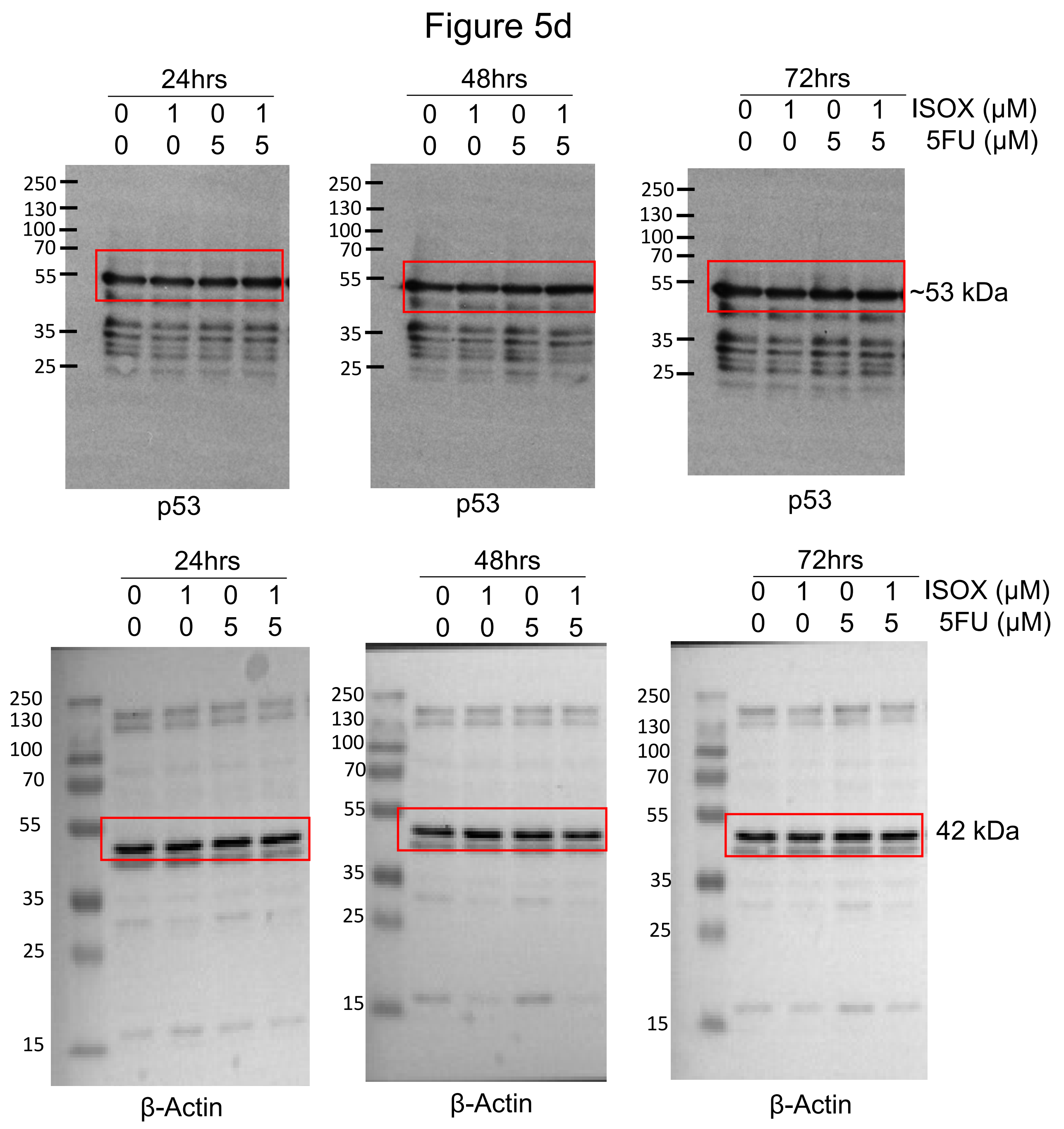

Supplementary Figure 25: Uncropped scans of the most important blots in Figure 5

Figure 5e

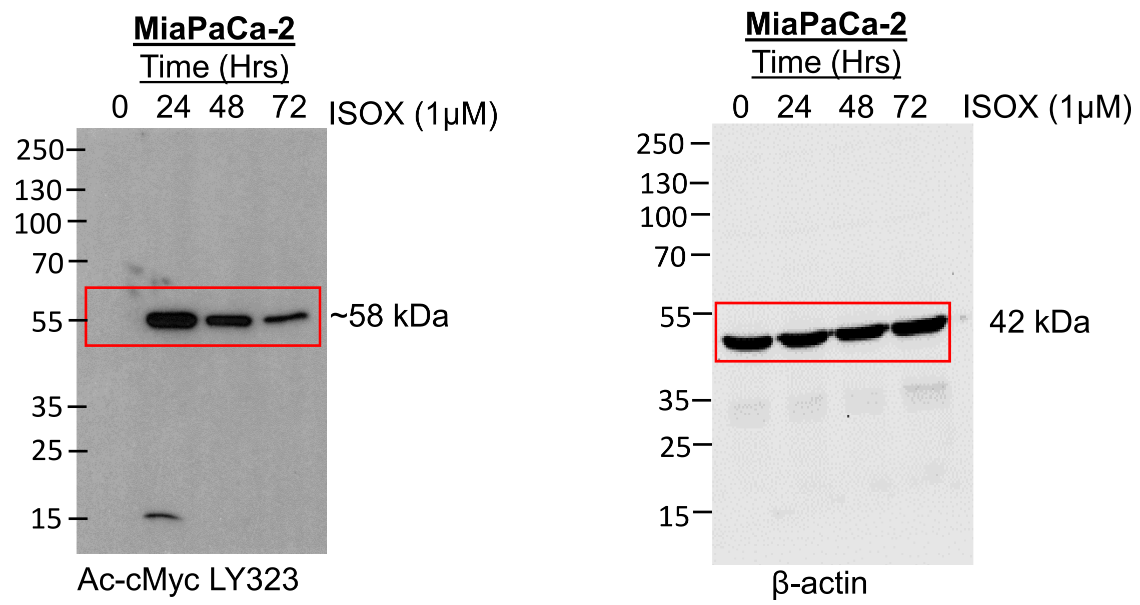

Figure 5f

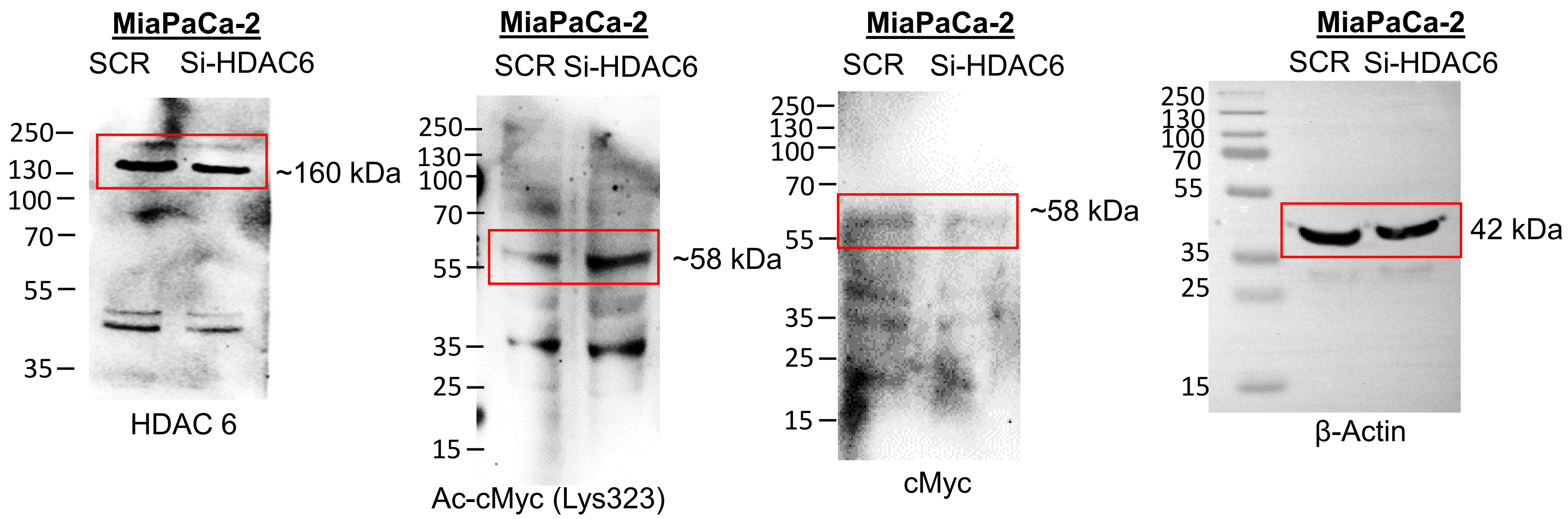

Figure 5i

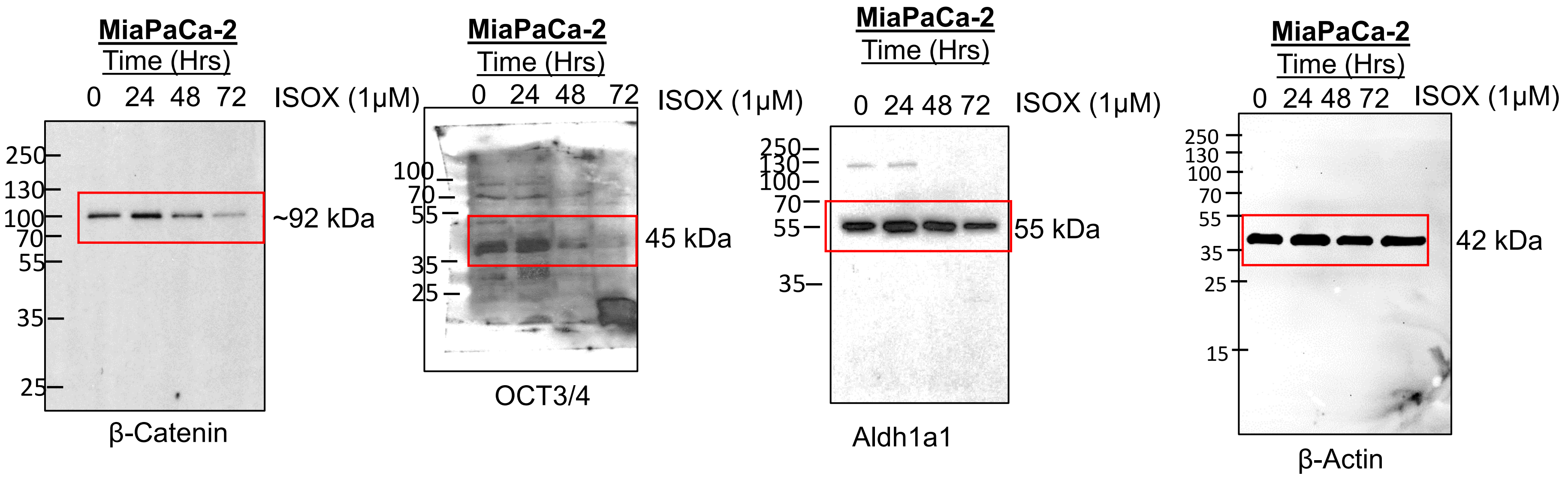

Supplementary Figure 26: Uncropped scans of the most important blots in Figure 5

Figure 5j

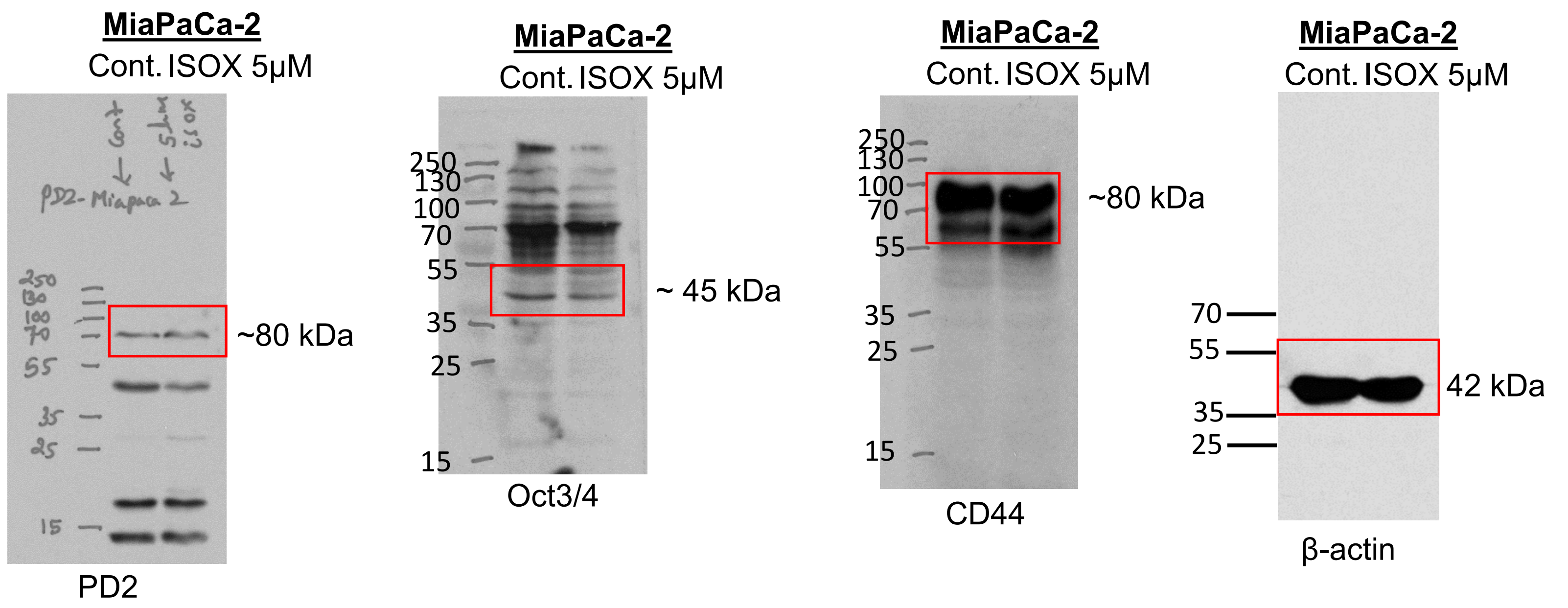

Figure 5k

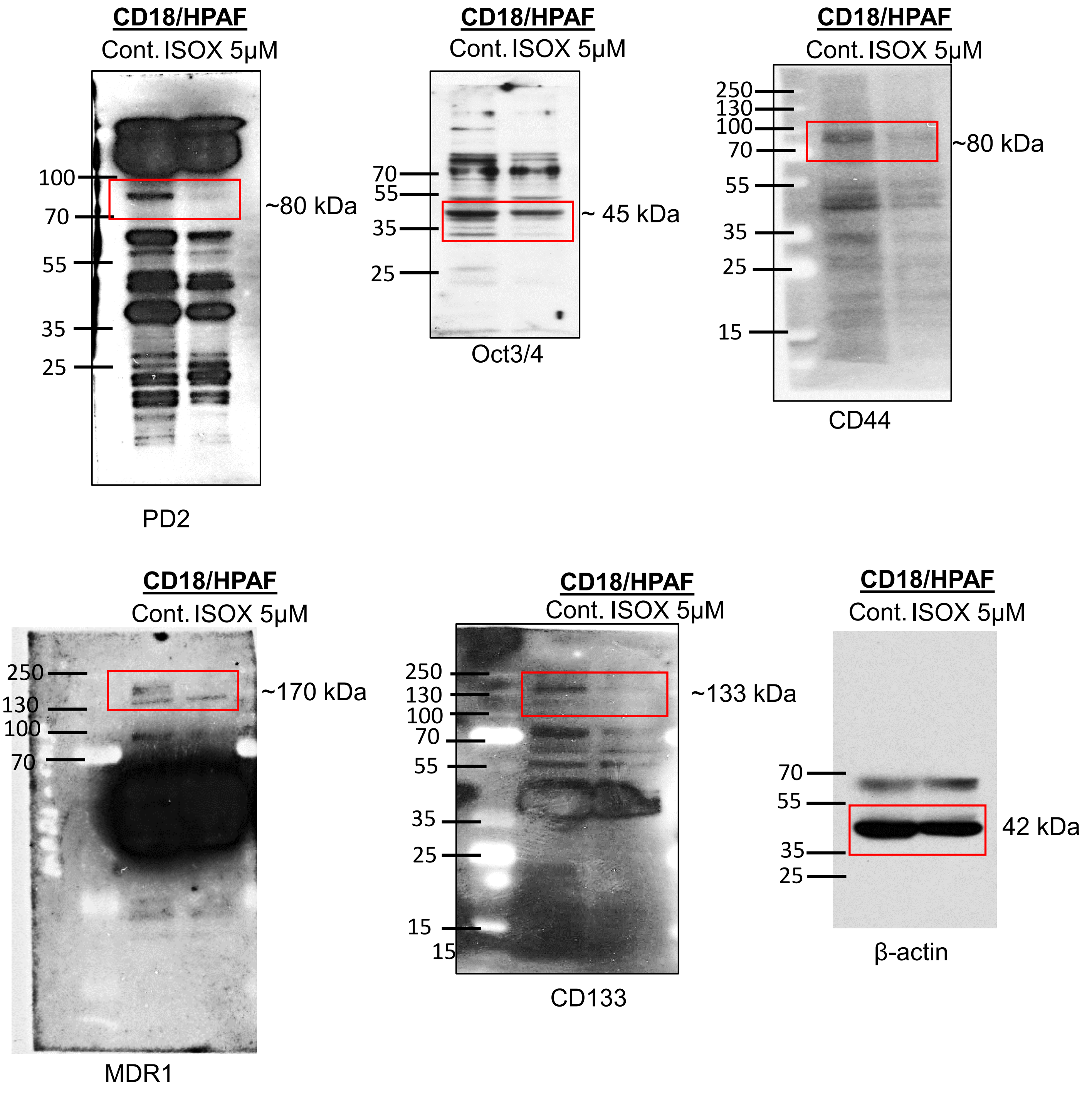

Supplementary Figure 27: Uncropped scans of the most important blots in Supplementary Figure 5e and f

Supplementary Figure 5e

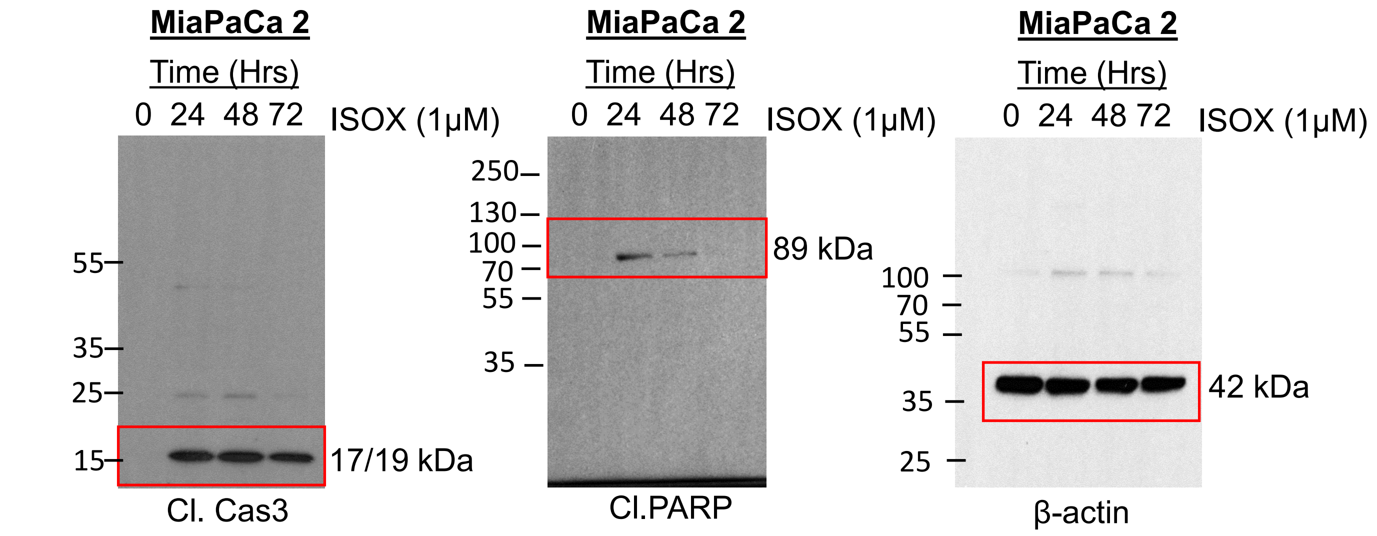

Supplementary Figure 5f

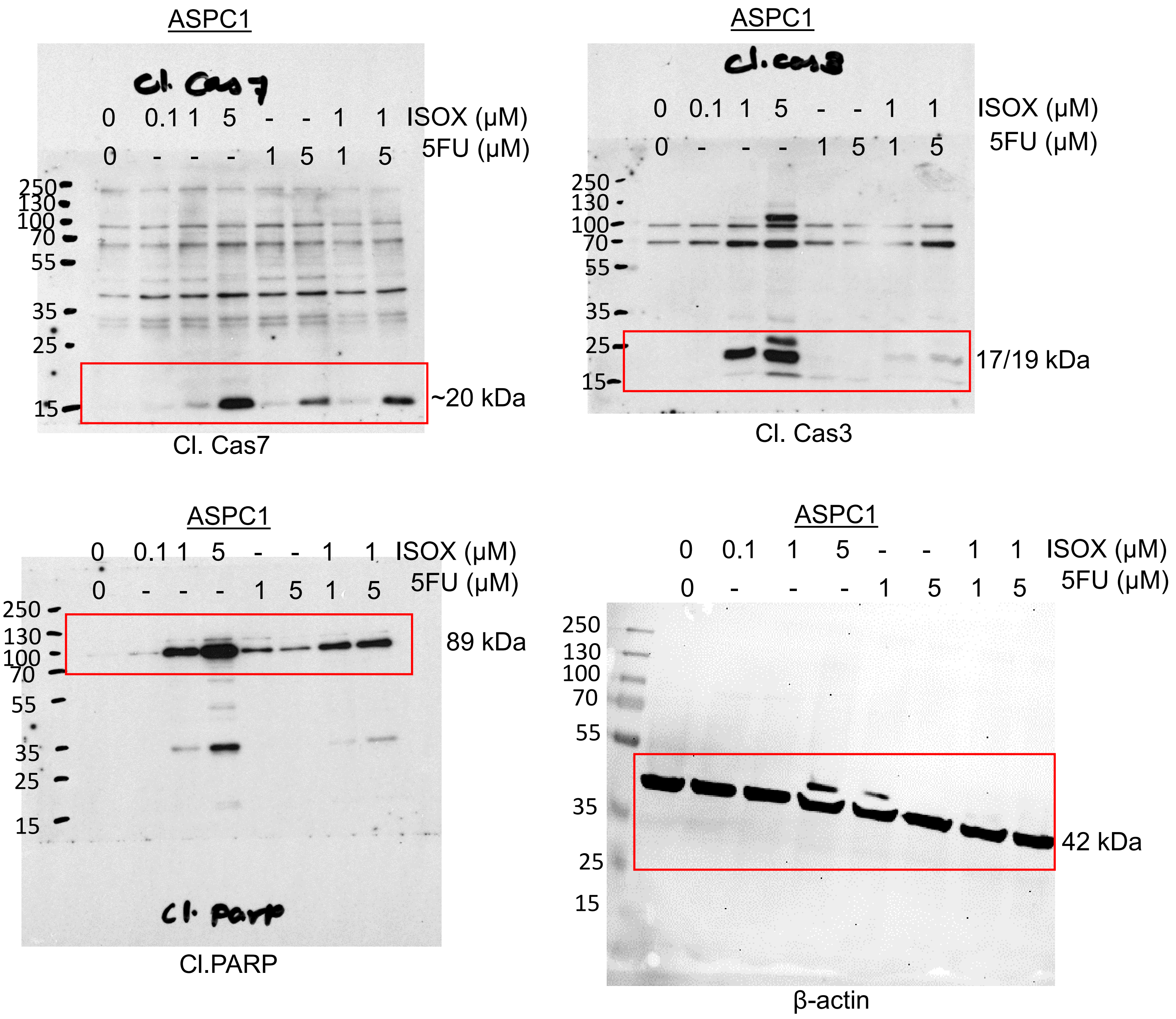

Supplementary Figure 28: Uncropped scans of the most important blots in Supplementary Figure 5f

Supplementary Figure 5f

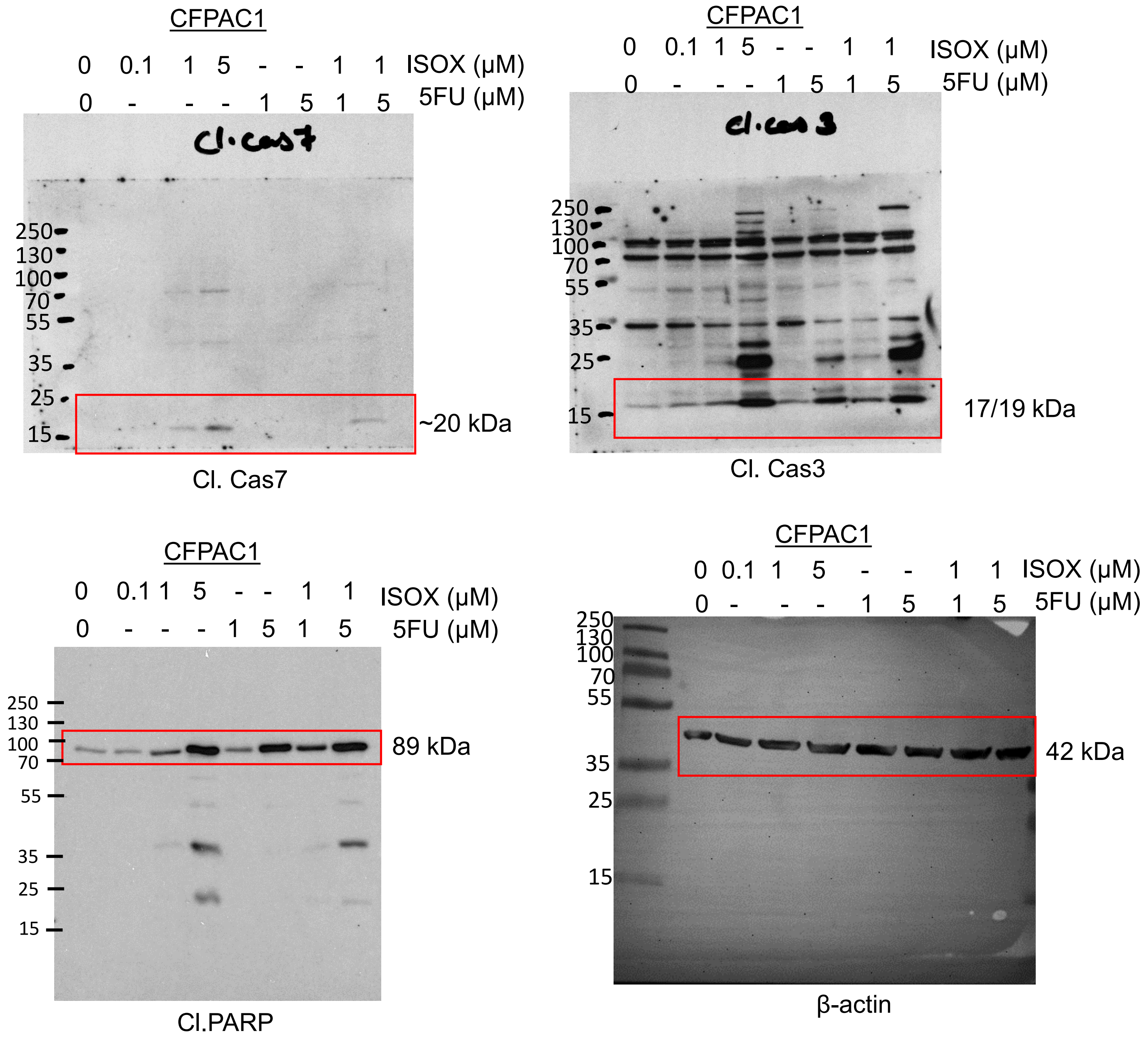

Supplementary Figure 29: Uncropped scans of the most important blots in Supplementary Figure 13

Supplementary Figure 13a

MiaPaCa 2

0 1 0 1 ISOX (μM)
0 0 5 5 5FU (μM)

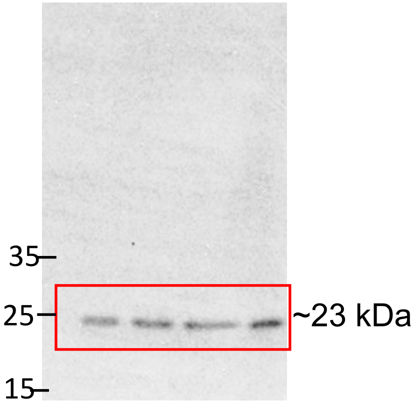

Bax

MiaPaCa 2

0 1 0 1 ISOX (μM)
0 0 5 5 5FU (μM)

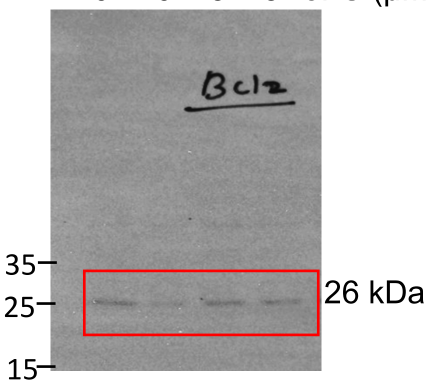

Bcl-2

MiaPaCa 2

0 1 0 1 ISOX (μM)
0 0 5 5 5FU (μM)

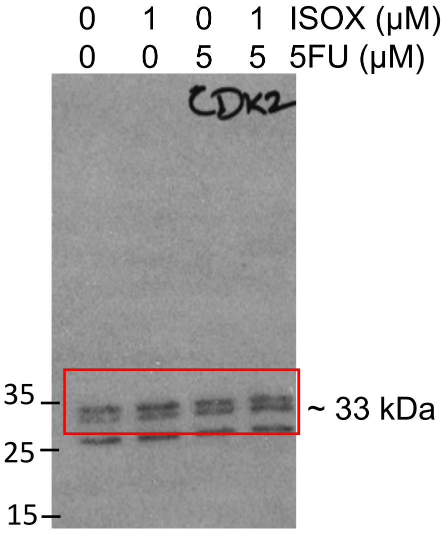

CDK2

MiaPaCa 2

0 1 0 1 ISOX (μM)
0 0 5 5 5FU (μM)

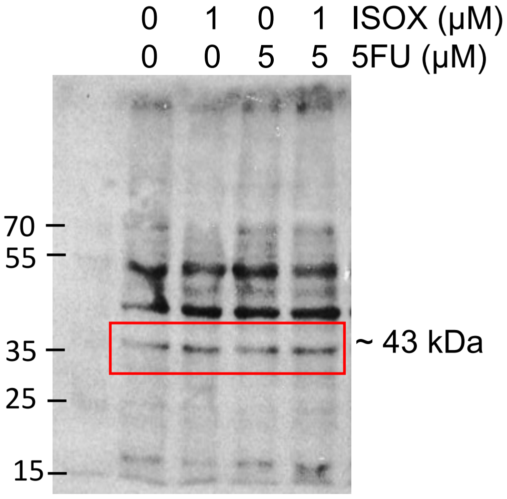

pP38

MiaPaCa 2

0 1 0 1 ISOX (μM)
0 0 5 5 5FU (μM)

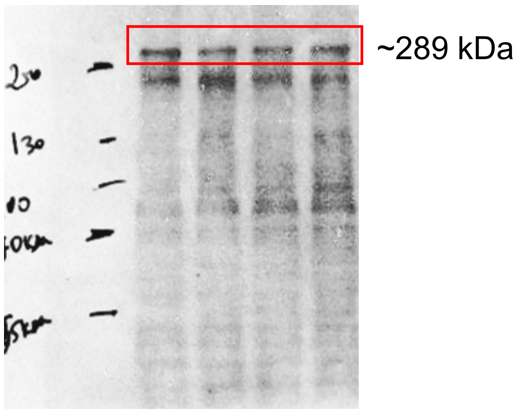

pmTOR

MiaPaCa 2

0 1 0 1 ISOX (μM)
0 0 5 5 5FU (μM)

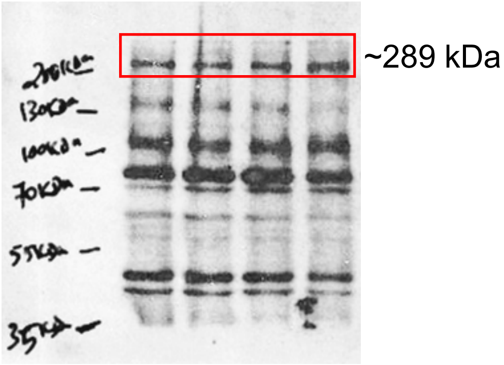

mTOR

MiaPaCa 2

0 1 0 1 ISOX (μM)
0 0 5 5 5FU (μM)

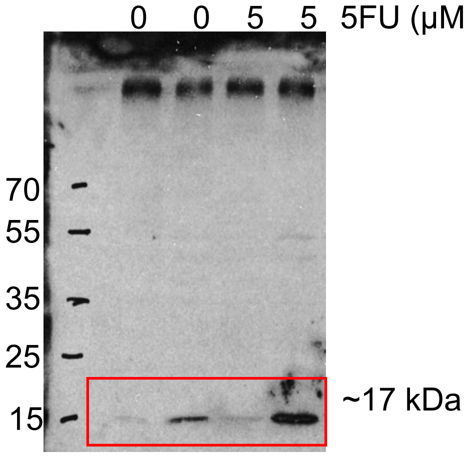

AC-Histone H3

MiaPaCa 2

0 1 0 1 ISOX (μM)
0 0 5 5 5FU (μM)

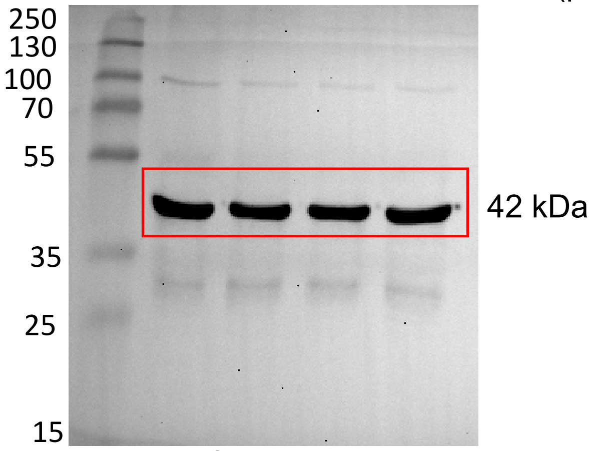

β-actin

Supplementary Figure 30: Uncropped scans of the most important blots in Supplementary Figure 13

Supplementary Figure 13b

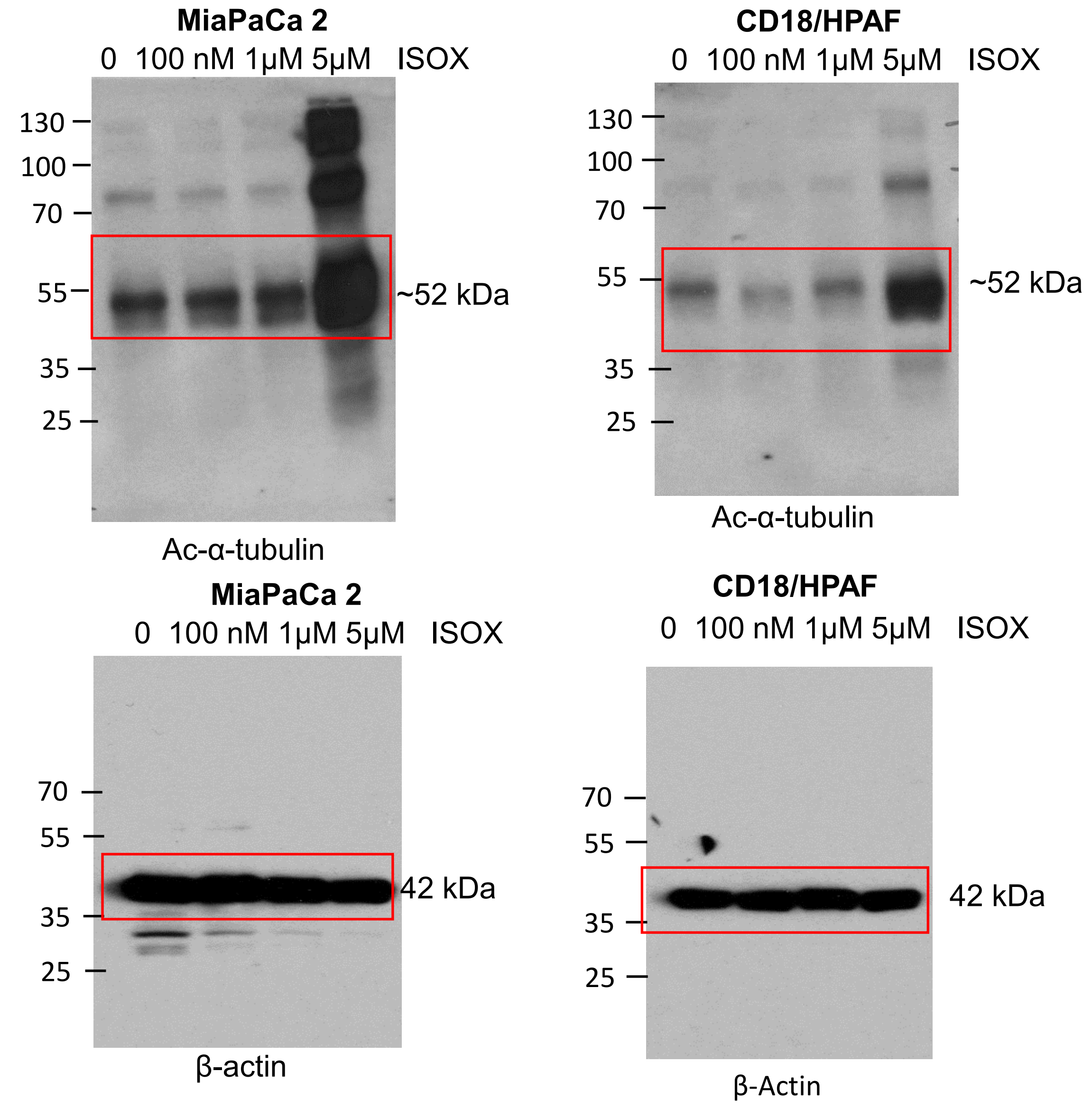

Supplementary Figure 13c

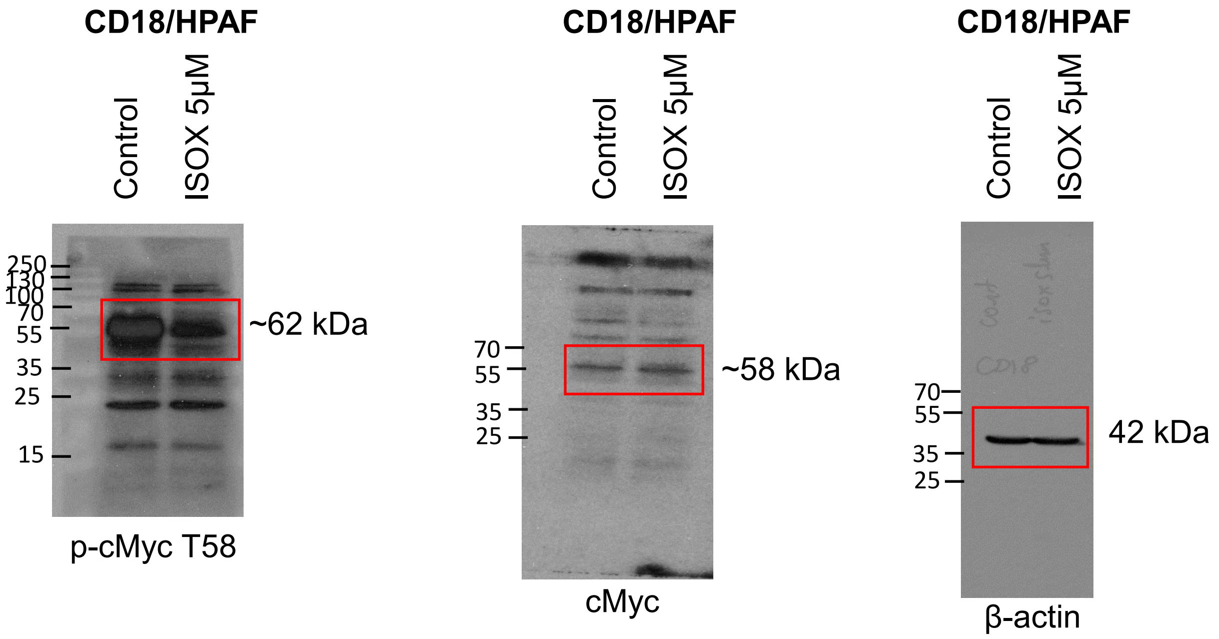

Supplement: Supplementary file 2 — Supplementary information [file 41698_2024_562_MOESM2_ESM.pdf]
